# Chronic obstructive pulmonary disease across three decades: trends, inequalities, and projections from the Global Burden of Disease Study 2021

**DOI:** 10.3389/fmed.2025.1564878

**Published:** 2025-03-24

**Authors:** Yan Wang, Ruiyang Han, Xiao Ding, Wenjia Feng, Runguo Gao, Anning Ma

**Affiliations:** ^1^School of Public Health, Shandong Second Medical University, Weifang, China; ^2^Institute of Public Health Crisis Management, Shandong Second Medical University, Weifang, China

**Keywords:** chronic obstructive pulmonary disease, Global Burden of Disease, decomposition analysis, inequality analysis, predictive analysis

## Abstract

**Objective:**

To assess the global burden of chronic obstructive pulmonary disease (COPD) and cross-country inequalities from 1990 to 2021 and project changes until 2045.

**Methods:**

Data on prevalence, mortality, and disability-adjusted life-years (DALYs) for COPD were extracted from the Global Burden of Disease Study 2021 (https://vizhub.healthdata.org/gbd-results/). Trends were analyzed globally, regionally, and nationally, considering population growth, aging, and epidemiological changes. Inequalities were quantified using the World Health Organization’s health equity framework. Future projections were estimated to 2045.

**Results:**

From 1990 to 2021, global age-standardized rates of COPD prevalence, mortality, and DALYs declined annually by −0.04, −1.75%, and −1.71%, respectively. However, absolute cases, deaths, and DALYs increased by 112.23, 49.06, and 40.23%, driven by population growth and aging. Men consistently showed higher age-standardized rates. East Asia reported the highest absolute cases and deaths, while South Asia had the largest DALYs. High-income North America and Oceania had the highest age-standardized rates, while Australasia and Eastern Europe saw the steepest declines in prevalence and mortality, respectively. Disparities in COPD burden across sociodemographic index levels widened over time. By 2045, absolute numbers of COPD cases, deaths, and DALYs are projected to rise despite declining age-standardized rates.

**Conclusion:**

While global age-standardized rates of COPD prevalence, mortality, and DALYs have declined, the absolute burden has increased due to demographic shifts. Persistent disparities in COPD burden, particularly in low- and middle-sociodemographic index regions, underscore the need for targeted prevention and management strategies.

## Introduction

Chronic obstructive pulmonary disease (COPD) is a preventable and treatable respiratory condition characterized by persistent airflow limitation due to airway and lung parenchymal abnormalities that are not fully reversible (1). It is a leading global respiratory disease, with major risk factors including tobacco use and occupational exposure to dust (2, 3). COPD is linked to systemic comorbidities such as renal dysfunction, hormonal imbalances, nutritional deficiencies, muscular dystrophy, osteoporosis, and anemia (4).

By 2021, COPD was the third leading cause of death globally, affecting approximately 213 million people and imposing a profound burden on healthcare systems and economies. Over half of COPD cases and 68% of COPD-related deaths and DALYs occurred in LMICs (5, 6), where limited access to diagnostics and treatment exacerbates health inequities. The disease contributes to systemic comorbidities, accelerates functional decline in aging populations, and reduces workforce productivity, costing an estimated $4.326 trillion globally between 2020 and 2050 (7, 8). Despite its significant societal impact, COPD remains underdiagnosed and incurable, underscoring the urgent need for equitable prevention strategies (5).

This study leverages data from the Global Burden of Disease (GBD) 2021 to comprehensively evaluate global, regional, and national COPD trends and disparities. It features a cross-country inequality assessment based on the World Health Organization’s health equity framework and includes projections of the global COPD burden through 2045.

## Materials and methods

### Study data

The GBD 2021 study employed standardized epidemiological methods to evaluate health losses from 369 diseases, injuries, and impairments, along with 87 risk factors, across 204 countries and territories, disaggregated by age and sex. Spatio-temporal Gaussian process regression smoothed incomplete datasets across age, time, and geography. Cross-country biases due to varying case definitions and study designs were adjusted using meta-regression with Bayesian priors, regularization, and trimming (MR-BRT).

This study extracted prevalence, mortality, and disability-adjusted life-years (DALYs) estimates for COPD from GBD 2021, with corresponding 95% uncertainty intervals (UI), standardized per 100,000 population (9). The sociodemographic index (SDI)—a composite metric of income, education, and fertility—was used to assess countries’ sociodemographic development, grouping them into five quintiles: low, low-middle, middle, high-middle, and high.

### Descriptive analysis

To comprehensively assess the COPD burden, a descriptive analysis was conducted at global, regional, and national levels. Global case numbers, crude rates, and age-standardized rates (ASRs) for COPD prevalence, mortality, and DALYs were examined by sex (male, female, and both sexes) from 1990 to 2021. Comparisons of case counts and ASRs between 1990 and 2021 were performed globally, regionally, nationally, and across the five SDI quintiles (10).

### Trend analysis

Temporal trend analysis is essential in epidemiology for guiding disease prevention strategies. This study assessed global, regional, and national COPD trends using the estimated annual percentage change (EAPC) as a primary metric. The EAPC of age-standardized rates (ASRs) was calculated using a linear regression model: $\ln\left(ASR\right)=\alpha +\beta \times \left(\mathrm{calendar year}\right);\mathrm{EAPC}=\left(\exp\left(\beta \right)-1\right)\times 100\%$ . The 95% confidence interval (CI) was derived from the model. An upward trend was indicated if both the EAPC estimate and its lower CI exceeded 0, while a downward trend was confirmed if both the EAPC estimate and its upper CI were below 0. If neither condition was met, the ASR was considered stable (11).

Joinpoint regression analysis further explored local COPD trends using the Joinpoint Trend Analysis software (2022). The method segmented the trend into multiple subperiods using inflection points. Annual percentage change (APC) and its 95% CI were calculated for each segment. Monte Carlo permutation with 4,499 random datasets estimated the average annual percentage change (AAPC). Multiple comparisons were corrected using the Bonferroni method. An increasing trend was identified if both APC/AAPC and its lower CI exceeded 0, while a decreasing trend was established if both values and the upper CI were below 0. Otherwise, the trend was considered stable (12).

To analyze age, period, and birth cohort effects, an age-period-cohort (APC) model was applied. This model assessed COPD risk through three temporal dimensions: (1) Age effect: Changes due to aging; (2) Period effect: Time-specific factors affecting the entire population; (3) Cohort effect: Shared risks among individuals born during the same timeframe. Due to multicollinearity among these variables, the intrinsic estimator (IE) method, based on principal component regression, was used for efficient estimation (13). The model structure is: $\ln\left(Y_{i,j,k}\right)=\mu +\alpha_{i}+\beta_{j}+\gamma_{k}+\varepsilon_{i,j,k}$ , where $Y_{i,j,k}$ the prevalence or mortality in the (*i*, *j*, *k*) group; μ is the intercept of the model; $\alpha_{i}$ , $\beta_{j}$ and $\gamma_{k}$ denoted the age, period, and birth cohort effect; $\varepsilon_{i,j,k}$ denoted the residual of the model.

Data were organized into 5-year age groups (<5 to 95+), 5-year intervals (1992–1996 to 2017–2021), and corresponding birth cohorts (1897–1901 to 1987–1991). The model’s estimated coefficients were exponentiated to determine the relative risks (RRs) for COPD prevalence and mortality (14).

### Decomposition analysis

To explore the drivers of changes in COPD-related DALYs from 1990 to 2021, we performed decomposition analyses focusing on two key factors: sex-specific differences (female and male) and the contributions of population growth, aging, and epidemiological changes. This approach disaggregated DALYs into components reflecting population size, age structure, and age-standardized DALY rates, which capture epidemiological dynamics (15). The calculation of DALYs at each location was expressed as: ${\mathrm{DALYs}}_{ay,py,ey}=\overset{n}{\underset{i=1}{\sum }}\left(a_{i,y}*p_{y}*e_{i,y}\right)$ . In this equation, ${\mathrm{DALYs}}_{ay,py,ey}$ referred to DALYs determined by age structure, population size, and DALY rate in year *y*; $a_{i,y}$ denoted the proportion of population for the age category *i* of the *n* age categories in year *y*; $p_{y}$ denoted the total population in year *y*; and $e_{i,y}$ denoted DALYs rate for the age category *i* in year *y*. The contribution of each factor was determined by isolating the effect of one factor while keeping the others constant.

### Cross-country inequality analysis

Monitoring health inequalities is essential for guiding policies and interventions aimed at reducing disparities in health outcomes. This study employed two commonly used metrics—absolute and relative inequality measures—to assess COPD-related health disparities across countries: the slope index of inequality (SII) and the concentration index (CI) (16). The SII was estimated by regressing national DALY rates against a relative position scale adjusted for sociodemographic trends. The CI was calculated from the Lorenz concentration curve, representing the cumulative proportion of DALYs against the cumulative population distribution ranked by SDI (17).

### Predictive analysis

To project COPD burden through 2045, we used the Bayesian age-period-cohort (BAPC) model with integrated nested Laplace approximation (INLA). This method was selected for three key advantages: (1) it avoids convergence challenges common in traditional MCMC sampling by directly approximating posterior distributions, (2) it robustly handles sparse data through hierarchical smoothing, and (3) it provides uncertainty intervals that account for demographic variability. These features make it particularly suitable for modeling COPD trends across heterogeneous populations with varying data quality (18).

## Results

### Descriptive analysis of COPD burden at global, regional, and national levels

From 1990 to 2021, global age-standardized rates (ASRs) of COPD prevalence, mortality, and DALYs declined significantly, yet absolute burdens surged due to population growth and aging. Men consistently bore a higher burden than women across all metrics (Supplementary Figure S1). Notable geographic disparities emerged: East Asia had the highest absolute cases (52.1 million) and deaths (1.32 million), while South Asia carried the largest DALYs burden (28.0 million). High-income North America and Oceania exhibited the most severe age-standardized prevalence (3298.9 per 100,000) and mortality rates (118.2 per 100,000), respectively (Tables 1–3).

**Table 1:** The ASR of prevalence of COPD in 1990 and 2021 for both sexes by SDI quintiles and by GBD regions, with EAPC from 1990 to 2021.

Location	1990	2021	EAPC (95% CI) 1990–2021
	ASR (95% UI)	ASR (95% UI)	
Global	2550.02 (2318.34–2806.32)	2512.86 (2293.93–2748.52)	−0.04 (−0.08 to −0.01)
High SDI	2602.27 (2359.28–2847.55)	2527.66 (2364.4–2701.8)	−0.04 (−0.1 to 0.02)
High-middle SDI	2523.42 (2287.88–2794.38)	2385.34 (2149.82–2648.74)	−0.22 (−0.25 to −0.19)
Middle SDI	2459.97 (2217.17–2717.88)	2463.19 (2208.84–2735.96)	−0.01 (−0.03 to 0.02)
Low-middle SDI	2621.01 (2378.69–2867.91)	2726.76 (2478.22–2,979)	0.14 (0.11–0.16)
Low SDI	2190.49 (1969.32–2409.53)	2337.52 (2119.39–2567.85)	0.21 (0.19–0.23)
Andean Latin America	1528.35 (1337.41–1749)	1658.68 (1461.64–1874.88)	0.32 (0.24–0.39)
Australasia	2056.21 (1810.47–2273.71)	1580.64 (1411.47–1793.95)	−0.86 (−0.95 to −0.77)
Caribbean	1581.11 (1399.43–1778.73)	1888.04 (1704.73–2076.77)	0.58 (0.51–0.64)
Central Asia	2301.81 (2066.36–2561.49)	2258.77 (2029.8–2508.18)	−0.05 (−0.11 to 0)
Central Europe	2344.47 (2109.49–2625.76)	2427.25 (2209.94–2672.32)	0.11 (0.1–0.13)
Central Latin America	2002.24 (1813.17–2211.32)	2221.43 (1999.23–2453.11)	0.27 (0.2–0.34)
Central sub-Saharan Africa	1751.34 (1555.48–1970.08)	1930.5 (1703.4–2184.23)	0.28 (0.26–0.29)
East Asia	2755.89 (2497.5–3025.18)	2484.43 (2224.88–2771.41)	−0.35 (−0.39 to −0.31)
Eastern Europe	2400.13 (2117.82–2699.58)	2125.62 (1896.41–2381.92)	−0.49 (−0.53 to −0.46)
Eastern sub-Saharan Africa	1581.68 (1390.72–1766.61)	1658.85 (1469.54–1852.14)	0.05 (−0.01 to 0.1)
High-income Asia Pacific	1748.52 (1527.77–1984.46)	1527.27 (1344.7–1724.48)	−0.31 (−0.4 to −0.22)
High-income North America	3115.6 (2822.58–3396.17)	3298.88 (3132.14–3451.88)	0.27 (0.14–0.39)
North Africa and Middle East	2103.45 (1880.95–2,353)	2512.32 (2248.38–2803.61)	0.55 (0.53–0.58)
Oceania	2646.62 (2408.24–2901.91)	2492.77 (2275.04–2748.51)	−0.22 (−0.22 to −0.21)
South Asia	2930.58 (2663.32–3198.92)	3019.1 (2729.72–3298.67)	0.12 (0.09–0.15)
Southeast Asia	2111.48 (1884.17–2362.84)	2136.77 (1904.59–2396.24)	−0.03 (−0.05 to −0.01)
Southern Latin America	1555.44 (1361.95–1758.56)	1482.66 (1320.77–1654.03)	−0.12 (−0.2 to −0.05)
Southern sub-Saharan Africa	2091.36 (1859.12–2340.65)	2127.98 (1882.89–2384.97)	0.01 (−0.02 to 0.04)
Tropical Latin America	2566.46 (2265.03–2867.24)	2546.83 (2252.36–2847.04)	−0.16 (−0.2 to −0.11)
Western Europe	2681.23 (2441.77–2941.19)	2590.99 (2375.15–2818.23)	−0.09 (−0.12 to −0.06)
Western sub-Saharan Africa	1562.6 (1365.07–1745.27)	1749.91 (1540.55–1959.17)	−0.04 (−0.08 to −0.01)

**Table 2:** The ASR of deaths of COPD in 1990 and 2021 for both sexes by SDI quintiles and by GBD regions, with EAPC from 1990 to 2021.

Location	1990	2021	EAPC (95% CI) 1990–2021
	ASR (95% UI)	ASR (95% UI)	
Global	71.92 (64.47–77.53)	45.22 (40.61–49.7)	−1.75 (−1.84 to −1.65)
High SDI	25.53 (23.71–26.5)	19.44 (17.26–20.66)	−0.97 (−1.04 to −0.9)
High-middle SDI	79.53 (70.61–86.61)	35.91 (30.78–40.69)	−3.15 (−3.39 to −2.91)
Middle SDI	123.89 (109.19–134.8)	57.45 (49.59–65.43)	−2.85 (−2.98 to −2.72)
Low-middle SDI	92.07 (74.17–107.46)	84.76 (75.8–93.78)	−0.12 (−0.23 to −0.02)
Low SDI	77.67 (61.91–91.44)	70.7 (63.35–79.76)	−0.11 (−0.27 to 0.05)
Andean Latin America	19.44 (16.88–21.8)	13.4 (10.93–16.29)	−0.88 (−0.98 to −0.77)
Australasia	29.42 (27.56–30.92)	18.89 (16.66–20.42)	−1.57 (−1.79 to −1.36)
Caribbean	18.48 (16.1–20.5)	19.85 (17.47–22.49)	0.14 (0–0.29)
Central Asia	36.33 (34.02–38.45)	21.27 (19.12–23.46)	−2.09 (−2.4 to −1.79)
Central Europe	30.65 (29.4–31.73)	16.03 (14.7–17.22)	−1.89 (−2.06 to −1.73)
Central Latin America	35.73 (33.58–36.9)	26.92 (23.7–29.64)	−1.03 (−1.15 to −0.92)
Central sub-Saharan Africa	52.33 (36.88–72.56)	42.82 (29.63–65.02)	−0.73 (−0.77 to −0.69)
East Asia	225.27 (194.01–250.33)	72.2 (59.32–85.26)	−4.2 (−4.42 to −3.98)
Eastern Europe	35.77 (34.15–36.74)	11.72 (10.79–12.62)	−4.43 (−4.77 to −4.09)
Eastern sub-Saharan Africa	42.29 (32.1–48.71)	29.91 (24.41–35.14)	−1.31 (−1.38 to −1.24)
High-income Asia Pacific	14.17 (12.76–15.08)	6.68 (5.7–7.37)	−2.53 (−2.74 to −2.33)
High-income North America	26.28 (24.3–27.29)	29.89 (26.2–31.63)	0.35 (0.15–0.55)
North Africa and Middle East	36.17 (28.39–41.23)	26.37 (23.19–29.31)	−0.87 (−1.03 to −0.72)
Oceania	144.27 (113.03–180.83)	118.21 (96.32–144.59)	−0.7 (−0.73 to −0.67)
South Asia	114.54 (89.16–136.08)	101.63 (90.55–114.34)	−0.22 (−0.36 to −0.08)
Southeast Asia	60.18 (48.55–68.27)	43.14 (38.24–48.54)	−1.18 (−1.3 to −1.06)
Southern Latin America	26.92 (25.28–28.4)	22.53 (20.53–24.07)	−0.43 (−0.71 to −0.14)
Southern sub-Saharan Africa	37.4 (32.85–44.78)	34.78 (31.93–37.34)	−0.39 (−0.75 to −0.03)
Tropical Latin America	44.64 (41.13–46.88)	26.32 (23.44–27.99)	−2.1 (−2.35 to −1.85)
Western Europe	24.66 (22.93–25.6)	17.82 (15.74–18.92)	−1 (−1.08 to −0.92)
Western sub-Saharan Africa	28.53 (22.44–33.71)	22.07 (19.45–25.05)	−0.65 (−0.74 to −0.56)

**Table 3:** The ASR of DALYs of COPD in 1990 and 2021 for both sexes by SDI quintiles and by GBD regions, with EAPC from 1990 to 2021.

Location	1990	2021	EAPC (95% CI) 1990–2021
	ASR (95% UI)	ASR (95% UI)	
Global	1492.64 (1342.46–1609.3)	940.66 (871.48–1014.59)	−1.71 (−1.79 to −1.63)
High SDI	589.8 (557.84–616.39)	471.22 (437.45–498.84)	−0.76 (−0.8 to −0.71)
High-middle SDI	1511.32 (1365.74–1635.67)	691.14 (621.83–772.74)	−3.06 (−3.25 to −2.87)
Middle SDI	2332.91 (2063.49–2546.31)	1076.67 (963.62–1201.24)	−2.83 (−2.94 to 2.72)
Low-middle SDI	1963.19 (1602.24–2252.75)	1707.9 (1558.88–1865.11)	−0.38 (−0.44 to −0.32)
Low SDI	1673.81 (1373.37–1936.99)	1457.94 (1318.76–1617.05)	−0.38 (−0.46 to −0.3)
Andean Latin America	375.29 (331.38–416.67)	261.42 (222.3–306.62)	−0.95 (−1.03 to −0.88)
Australasia	610.45 (577.57–639.32)	376.2 (343.09–400.39)	−1.71 (−1.89 to −1.52)
Caribbean	396.98 (342.42–442.74)	439.53 (388.88–499.3)	0.25 (0.12–0.38)
Central Asia	811.63 (767.26–853.69)	498.6 (452.01–546.1)	−2.07 (−2.36 to −1.78)
Central Europe	679.3 (652.23–705.63)	413.12 (382.55–444.56)	−1.45 (−1.59 to −1.31)
Central Latin America	660.88 (632.68–684.66)	512.35 (462.65–560.08)	−0.99 (−1.1 to −0.88)
Central sub-Saharan Africa	1150.66 (851.36–1503.88)	975.91 (720.5–1344.93)	−0.61 (−0.64 to −0.57)
East Asia	3760.9 (3272.11–4173.78)	1217.69 (1043.87–1422.79)	−4.12 (−4.31 to −3.94)
Eastern Europe	788.92 (757.69–815.47)	324.42 (301.04–350.1)	−3.7 (−4.01 to −3.38)
Eastern sub-Saharan Africa	959.7 (762.97–1088.96)	713.24 (593–816.07)	−1.15 (−1.22 to −1.09)
High-income Asia Pacific	311.78 (286.99–335.85)	182.14 (162.64–202.95)	−1.69 (−1.79 to −1.59)
High-income North America	703.28 (663.83–742)	736.05 (685.79–776.49)	0.13 (−0.02 to 0.28)
North Africa and Middle East	794.61 (655.01–882.01)	599.18 (546.07–658.22)	−0.87 (−0.97 to −0.77)
Oceania	2919.73 (2293.23–3660.43)	2351.49 (1931.26–2854.06)	−0.75 (−0.8 to −0.7)
South Asia	2424.19 (1918.36–2829.14)	2049.22 (1862.71–2268.73)	−0.47 (−0.55 to −0.39)
Southeast Asia	1255.6 (1042.88–1403.69)	914.73 (822.27–1016.35)	−1.13 (−1.22 to −1.05)
Southern Latin America	554.35 (527.28–583.08)	436.03 (405.72–461.73)	−0.66 (−0.87 to −0.45)
Southern sub-Saharan Africa	896.69 (806.97–1006.53)	864.13 (800.37–929.92)	−0.23 (−0.54 to 0.08)
Tropical Latin America	880.79 (834.58–919.82)	554.73 (513.83–585.11)	−1.92 (−2.16 to −1.68)
Western Europe	520.26 (495.21–542.9)	390.81 (361.46–415.88)	−0.88 (−0.96 to −0.81)
Western sub-Saharan Africa	681.87 (558.27–780.37)	567.27 (510.78–631.22)	−0.46 (−0.54 to −0.39)

**Figure 1:**
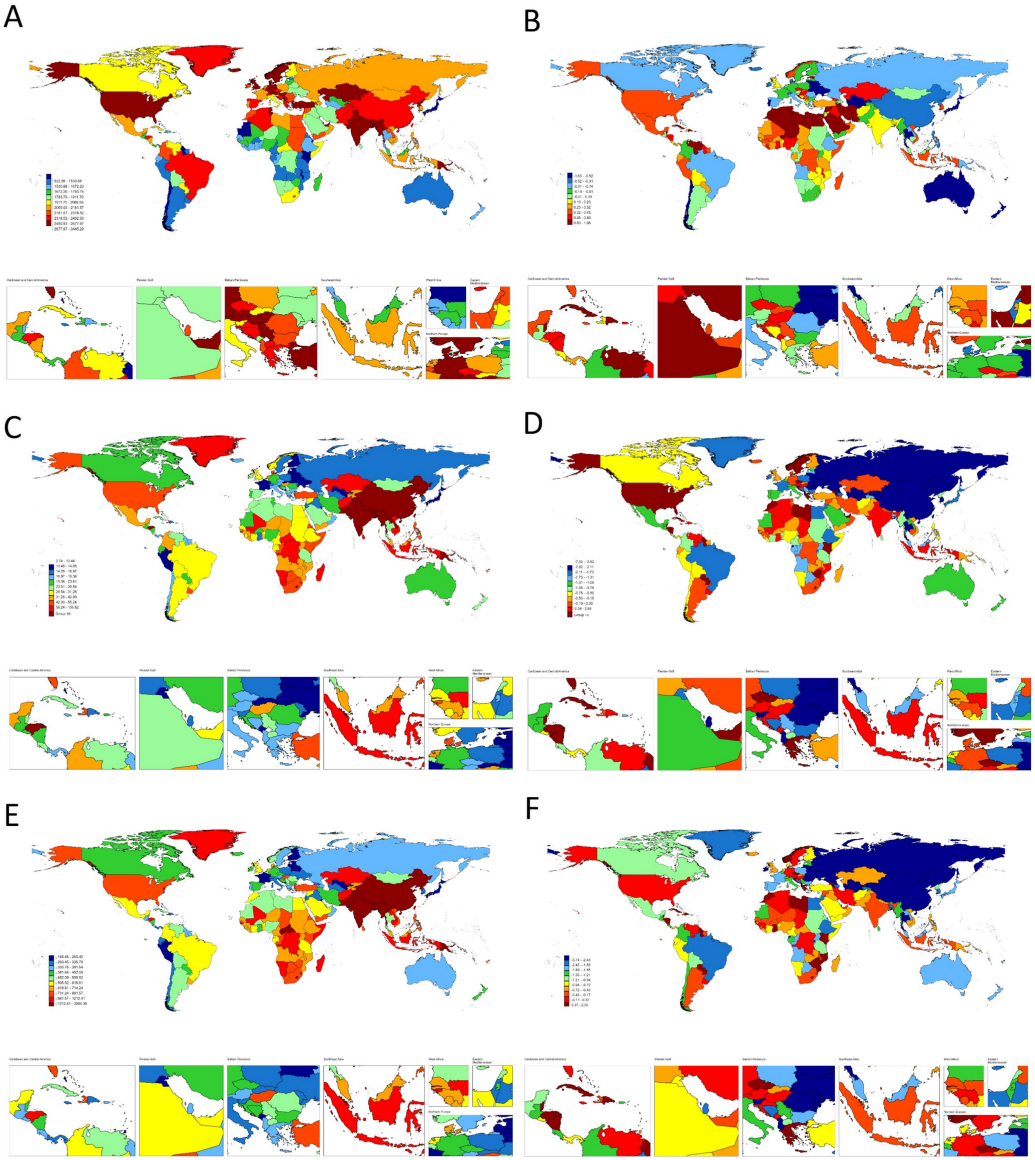
**(A)** The ASR of prevalence in 2021. **(B)** The trend in ASR of prevalence (EAPC) from 1990 to 2021. **(C)** The ASR of deaths in 2021. **(D)** The trend in ASR of deaths (EAPC) from 1990 to 2021. **(E)** The ASR of DALYs in 2021. **(F)** The trend in ASR of DALYs (EAPC) from 1990 to 2021; of COPD worldwide. ASR, age-standardized rate; EAPC, estimated annual percentage change; DALYs, disability-adjusted life-years; COPD, chronic obstructive pulmonary disease.

At the national level, China and India dominated absolute burdens, whereas the United States and Papua New Guinea showed extreme age-standardized rates (Figure 1 and Supplementary Tables S1–S3). Socioeconomic inequalities persisted: while middle SDI regions accounted for the largest case numbers, low-middle SDI areas suffered the highest ASRs (prevalence: 2726.8; mortality: 84.8 per 100,000). Full data are detailed in Tables 1–3; Supplementary Tables S1–S3.

### Local trends in COPD burden using joinpoint regression analysis

**Figure 2:**
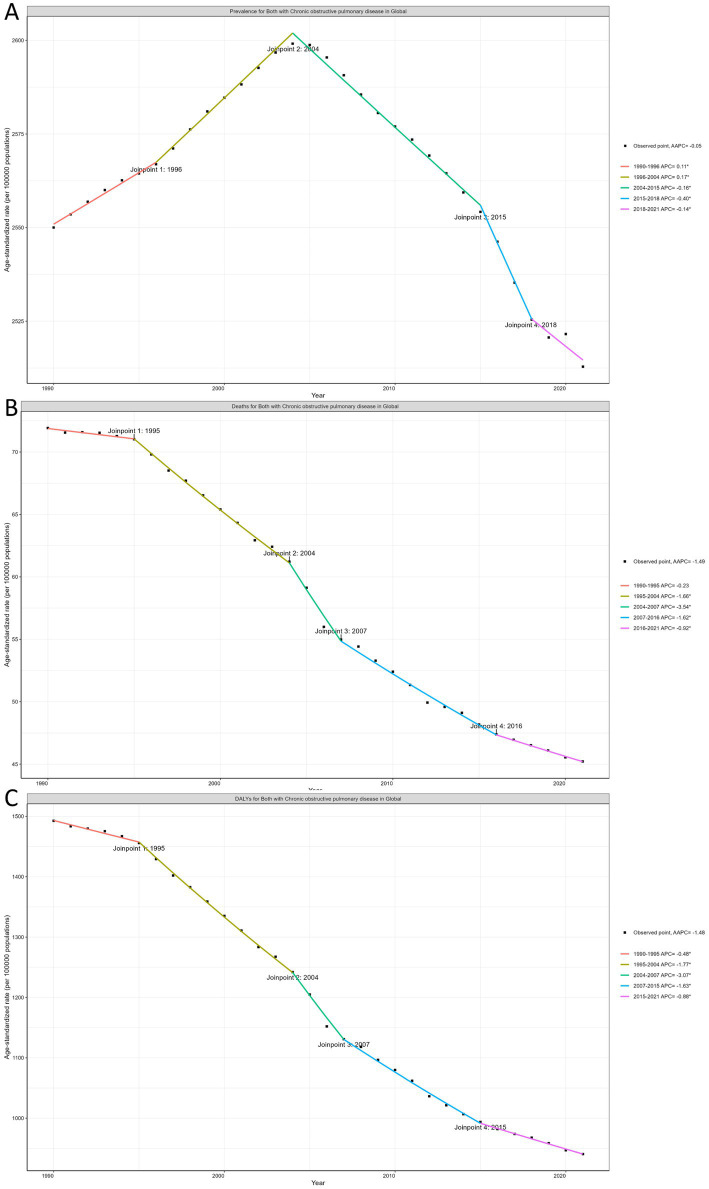
**(A)** The joinpoint regression analysis on the ASR of prevalence. **(B)** The joinpoint regression analysis on the ASR of mortality. **(C)** The joinpoint regression analysis on the ASR of DALYs; of COPD globally. ASR, age-standardized rate; DALYs, disability-adjusted life-years; COPD, chronic obstructive pulmonary disease.

Globally, ASRs of prevalence, mortality, and DALYs associated with COPD have shown a significant overall decline, although distinct patterns are evident across specific time periods. For age-standardized prevalence rates, the joinpoint regression identified 2004 as a critical inflection point, with age-standardized prevalence rates shifting from an upward trend (1990–2004) to a sustained decline (2004–2021). Age-standardized mortality and DALYs rates exhibited similar downward trends across five distinct periods. The smallest reduction was noted between 1990 and 1995, while the most significant decline occurred from 2004 to 2007 (Figure 2).

### Age-period-cohort analysis on COPD prevalence and prevalence

**Figure 3:**
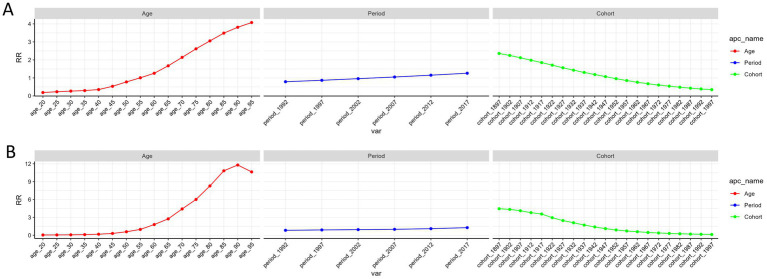
The effects of age, period, and birth cohort on the relative risk of COPD prevalence **(A)** and mortality **(B)**. COPD, chronic obstructive pulmonary disease.

The age-period-cohort analysis results for COPD prevalence and mortality are illustrated in Figure 3, with sex-specific subgroup analyses provided in Supplementary Figure S2. After adjusting for period and birth cohort effects, age emerged as a significant determinant of COPD prevalence and mortality risks. Both trends showed a linear upward trajectory, with the highest prevalence risk observed in the 95–99 age group, reflecting a 4.1-fold increase in relative risk, and the highest mortality risk seen in the 90–94 age group, corresponding to an 11.8-fold increase in relative risk (Table 4). Notably, women displayed a higher relative risk of prevalence compared to men in the 20–44 and 90–95 age groups, as well as a higher relative risk of mortality in the 20–39 and 95–99 age groups (Supplementary Tables S4, S5).

**Table 4:** RRs of COPD prevalence and mortality for both sexes due to age, period, and birth cohort effects.

Factor	Prevalence		Deaths	
	RR (95% CI)	*p*	RR (95% CI)	*p*
Age (years)				
20 to 24	0.1888 (0.1887–0.1889)	<0.001	0.0472 (0.0465–0.0479)	<0.001
25 to 29	0.2324 (0.2323–0.2325)	<0.001	0.0558 (0.0552–0.0565)	<0.001
30 to 34	0.2685 (0.2684–0.2686)	<0.001	0.0763 (0.0756–0.0771)	<0.001
35 to 39	0.296 (0.2959–0.2961)	<0.001	0.1068 (0.1059–0.1077)	<0.001
40 to 44	0.3534 (0.3533–0.3536)	<0.001	0.1782 (0.177–0.1794)	<0.001
45 to 49	0.534 (0.5338–0.5342)	<0.001	0.3045 (0.3029–0.3062)	<0.001
50 to 54	0.777 (0.7768–0.7772)	0.05	0.5893 (0.5868–0.5919)	<0.001
55 to 59	1.0066 (1.0064–1.0069)	<0.001	0.9809 (0.9773–0.9844)	<0.001
60 to 64	1.2579 (1.2576–1.2582)	<0.001	1.8005 (1.7953–1.8057)	<0.001
65 to 69	1.6706 (1.6702–1.671)	<0.001	2.7511 (2.7446–2.7577)	<0.001
70 to 74	2.1391 (2.1386–2.1397)	<0.001	4.4203 (4.4114–4.4291)	<0.001
75 to 79	2.6144 (2.6137–2.615)	<0.001	6.012 (6.0005–6.0235)	<0.001
80 to 84	3.0542 (3.0533–3.0551)	<0.001	8.2858 (8.2684–8.3033)	<0.001
85 to 89	3.4858 (3.4846–3.487)	<0.001	10.6433 (10.5941–10.6928)	<0.001
90 to 94	3.8114 (3.8097–3.8131)	<0.001	10.8277 (10.8001–10.8554)	<0.001
95 to 99	4.0739 (4.071–4.0768)	<0.001	11.801 (11.7625–11.8396)	<0.001
Period				
1992 to 1996	0.7884 (0.7882–0.7885)	<0.001	0.8338 (0.8321–0.8354)	<0.001
1997 to 2001	0.8678 (0.8677–0.868)	<0.001	0.8939 (0.8926–0.8953)	<0.001
2002 to 2006	0.9585 (0.9583–0.9586)	<0.001	0.9475 (0.9465–0.9486)	<0.001
2007 to 2011	1.0505 (1.0503–1.0507)	<0.001	1.0003 (0.9992–1.0014)	0.615
2012 to 2016	1.1516 (1.1514–1.1517)	<0.001	1.1075 (1.1059–1.1091)	<0.001
2017 to 2021	1.2605 (1.2603–1.2607)	<0.001	1.2782 (1.2757–1.2806)	<0.001
Birth cohort				
1897 to 1901	2.3567 (2.3506–2.3627)	<0.001	4.4566 (4.401–4.5128)	<0.001
1902 to 1906	2.2472 (2.2445–2.2499)	<0.001	4.3467 (4.3196–4.3739)	<0.001
1907 to 1911	2.1193 (2.1177–2.1209)	<0.001	4.1215 (4.1036–4.1395)	<0.001
1912 to 1916	1.9821 (1.981–1.9833)	<0.001	3.7938 (3.7803–3.8072)	<0.001
1917 to 1921	1.8508 (1.8499–1.8517)	<0.001	3.5698 (3.5588–3.5807)	<0.001
1922 to 1926	1.709 (1.7083–1.7097)	<0.001	2.9506 (2.9423–2.9589)	<0.001
1927 to 1931	1.5619 (1.5613–1.5625)	<0.001	2.4692 (2.4624–2.4761)	<0.001
1932 to 1936	1.4304 (1.4299–1.4309)	<0.001	2.1015 (2.0953–2.1077)	<0.001
1937 to 1941	1.3038 (1.3034–1.3043)	<0.001	1.7148 (1.7092–1.7205)	<0.001
1942 to 1946	1.188 (1.1877–1.1884)	<0.001	1.3981 (1.3928–1.4034)	<0.001
1947 to 1951	1.0715 (1.0711–1.0718)	<0.001	1.1141 (1.1093–1.1189)	<0.001
1952 to 1956	0.9573 (0.957–0.9576)	<0.001	0.8909 (0.8865–0.8954)	<0.001
1957 to 1961	0.8546 (0.8543–0.8548)	<0.001	0.7192 (0.7151–0.7233)	<0.001
1962 to 1966	0.7593 (0.759–0.7595)	<0.001	0.5932 (0.5893–0.597)	<0.001
1967 to 1971	0.675 (0.6748–0.6752)	<0.001	0.4732 (0.4697–0.4767)	<0.001
1972 to 1976	0.6038 (0.6035–0.604)	<0.001	0.383 (0.3797–0.3864)	<0.001
1977 to 1981	0.542 (0.5418–0.5423)	<0.001	0.3078 (0.3044–0.3112)	<0.001
1982 to 1986	0.4835 (0.4833–0.4838)	<0.001	0.2461 (0.2427–0.2495)	<0.001
1987 to 1991	0.4307 (0.4304–0.4309)	<0.001	0.2 (0.1965–0.2036)	<0.001
1992 to 1996	0.3852 (0.3849–0.3855)	<0.001	0.1632 (0.1592–0.1672)	<0.001
1997 to 2001	0.3483 (0.3478–0.3487)	<0.001	0.1328 (0.1272–0.1386)	<0.001

Analysis controlling for age and birth cohort effects indicated that the period effect had a modest influence on COPD prevalence and mortality. Between 1992 and 2021, the period effect exhibited a relatively stable but slightly increasing trend, with relative risks rising 1.26-fold for prevalence and 1.27-fold for mortality, peaking in 2017 (Table 4). Further sex-specific analysis revealed that women experienced a higher prevalence risk than men in 1992, 2012, and 2017, and a higher mortality risk in 1992 and 1997 (Supplementary Tables S4, S5).

After adjusting for age and period effects, birth cohort effects were found to significantly impact COPD prevalence and mortality. Earlier birth cohorts exhibited a higher relative risk compared to more recent cohorts, with a progressive decline in risk observed from the 1897–1901 cohort to the 2017–2021 cohort (Table 4). Additionally, women born between 1962 and 1997 showed a slightly elevated risk of COPD prevalence compared to men. Similarly, women from the 1932–2001 birth cohorts had a marginally higher mortality risk than their male counterparts (Supplementary Tables S4, S5).

### Decomposition analysis on COPD DALYs

**Figure 4:**
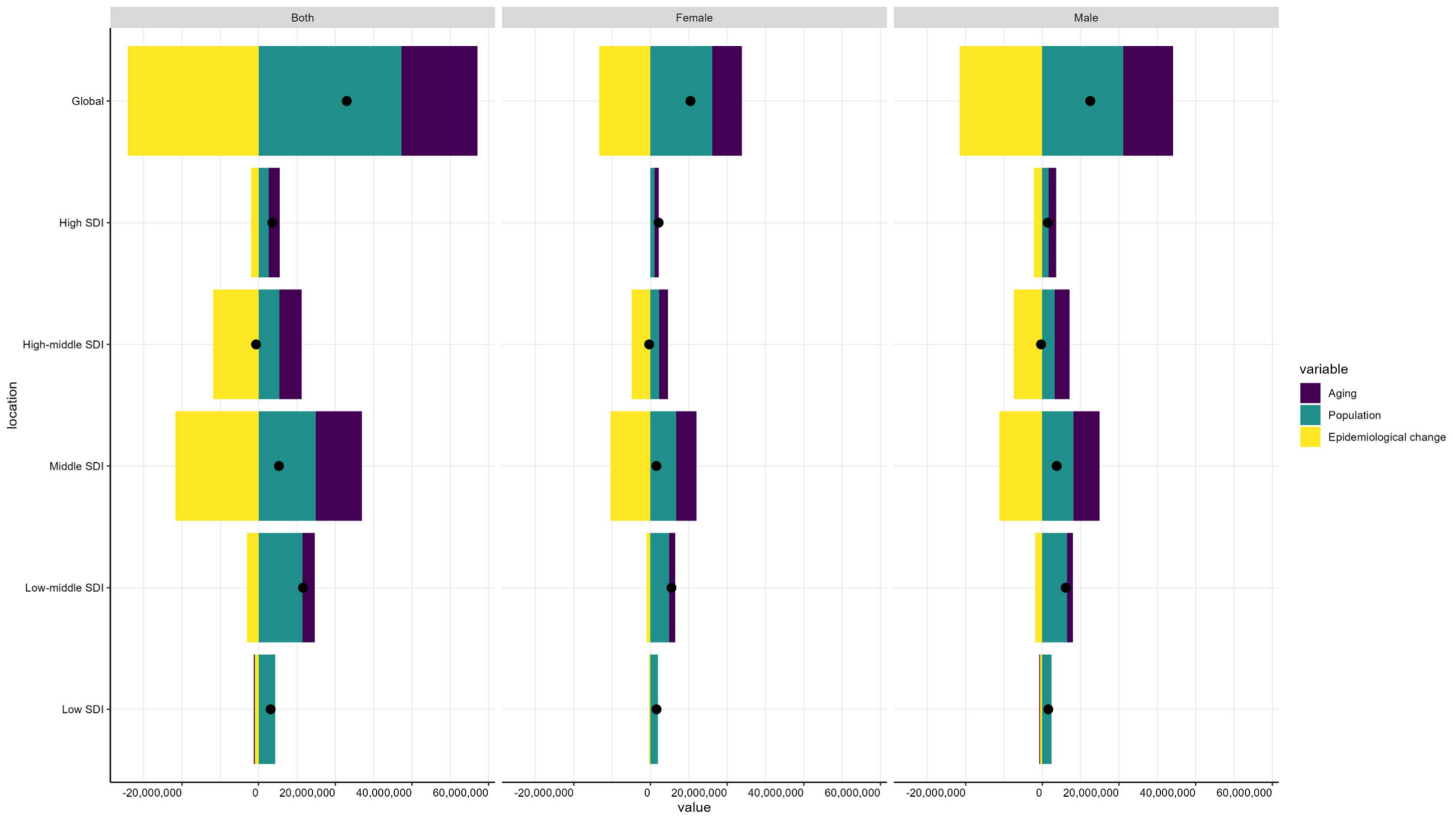
Changes in DALYs of COPD according to aging, population growth and epidemiological change from 1990 to 2021 at global level by SDI quintile and by sexes. The black dot denotes the overall value of the change resulting from all three components. For each component, the magnitude of a positive value suggests a corresponding increase in COPD DALYs attributed to the component; the magnitude of a negative value suggests a corresponding decrease in COPD DALYs attributed to the component. DALYs, disability-adjusted life-years; COPD, chronic obstructive pulmonary disease; SDI, sociodemographic index.

Over the past 32 years, the global burden of DALYs associated with COPD has risen markedly. The only reduction occurred in high-middle SDI quintiles, while the most substantial increase was observed in low-middle SDI quintiles (Figure 4). Population growth emerged as the primary driver of this global increase, accounting for 163.31% of the change, with aging contributing an additional 86.34%. In contrast, epidemiological change counteracted this trend, resulting in a 148.65% decline in global DALYs. In the low-middle SDI quintile, DALYs experienced the most significant rise, with 98.88% of the increase attributed to population growth, 27.45% to aging, and 26.33% to reductions in epidemiological improvements. For the high-middle SDI quintile, epidemiological change had the most profound effect, causing a dramatic 1794.99% decline, although both aging and population growth contributed to an overall increase in DALYs. Other SDI regions also experienced rising DALYs due to COPD, with population growth consistently identified as the most influential factor. This impact was particularly pronounced in the middle SDI quintile, where population growth led to a 280.39% increase in DALYs (Supplementary Table S6).

Sex-specific analyses reveal that aging, population growth, and epidemiological changes have distinct effects on the number of DALYs for COPD. Globally, men bear a higher burden of DALYs than women. For males, aging and population growth contributed more significantly to the increase in DALYs, while reductions driven by epidemiological change were also more pronounced in men compared to women. Across all SDI quintiles, except for the High-middle SDI group, population growth emerged as the primary factor driving increases in DALYs, while epidemiological improvements were the most significant contributors to DALYs reductions. Importantly, the impact of population growth and aging on DALYs was consistently greater for men than for women in all SDI quintiles, except in the Middle SDI group, where the contributions of these factors were more balanced between genders.

Meanwhile, since the jointpoint regression analysis showed that the global age-standardized incidence rate of COPD showed a significant turnaround in 2004, the decomposition analysis was conducted with two time periods, 1990–2004 and 2004–2021, and it was found that the global age-standardized incidence rate of COPD from 1990–2004 showed an upward trend, with the main drivers being population growth (141.45%) and aging (62.6%), and a significant decrease in ASR from 2004–2021, mainly attributed to epidemiologic improvement (−145.7%). In high SDI regions, population aging was the main driver from 1990–2004; after 2004, epidemiologic changes significantly reduced the burden of COPD; in low SDI regions, population growth and epidemiologic changes led to an increase in the burden of COPD in 1990–2004; and after 2004, despite the contribution of epidemiologic changes, population growth remained the main driver, see Supplementary Figure S3.

### Cross-country inequality analysis

**Figure 5:**
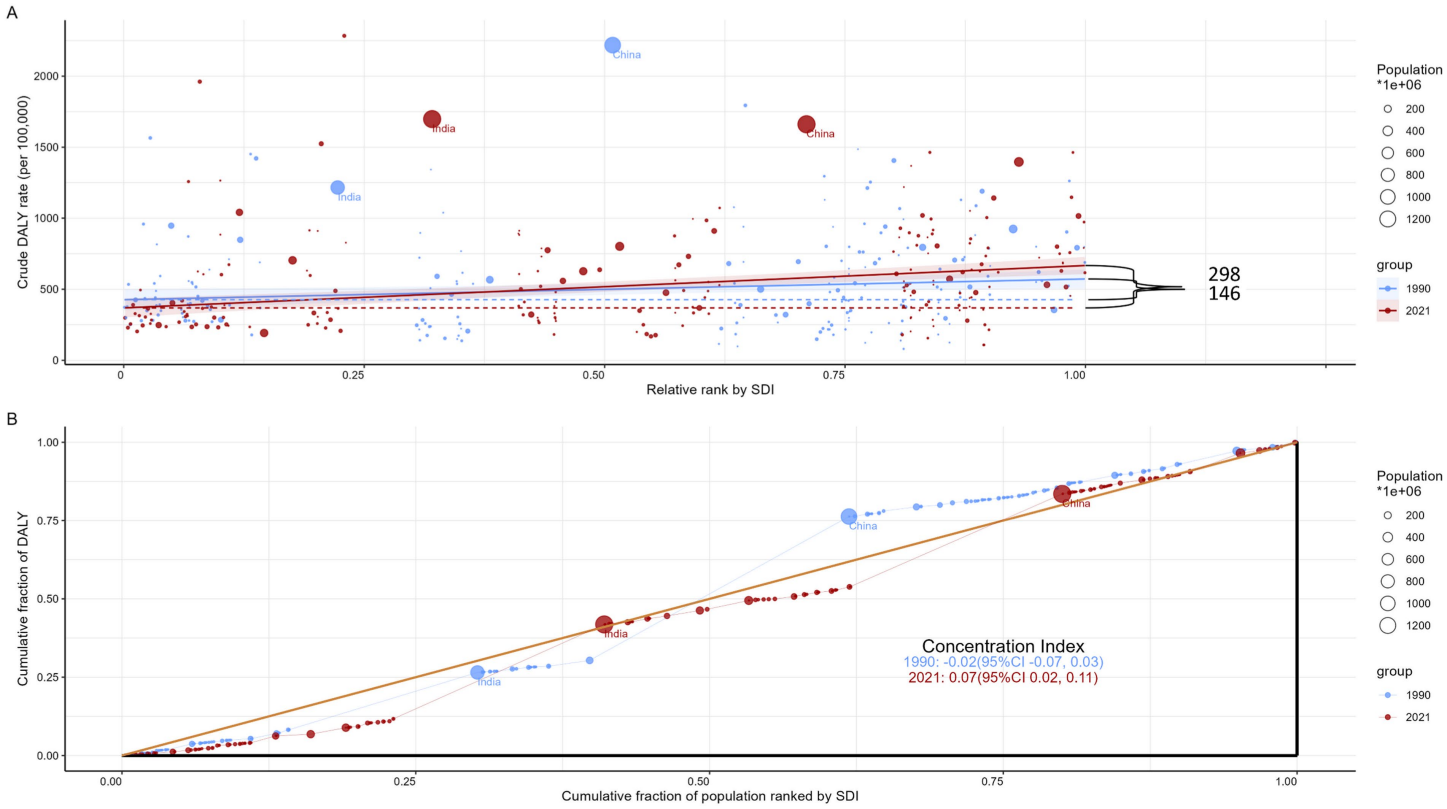
SDI-related health inequality regression **(A)** and concentration **(B)** curves for the DALYs of COPD worldwide, 1990 and 2021. SDI, sociodemographic index; DALYs, disability-adjusted life-years; COPD, chronic obstructive pulmonary disease.

This study highlights significant absolute and relative inequalities in the burden of COPD across SDI levels, which have become more pronounced over time (Figure 5). Notably, DALYs are predominantly concentrated in countries with higher socio-demographic development. According to the inequality index, the disparity in DALYs per 100,000 people between countries with the highest and lowest SDI levels was 146.12 (95% CI: 22.64–269.61) in 1990, widening to 298.25 (95% CI: 182.28–414.21) by 2021. Additionally, the concentration index, which measures relative inequality, shifted from −0.02 (95% CI: −0.07–0.03) in 1990 to 0.07 (95% CI: 0.02–0.11) in 2021. This change indicates a persistent yet slightly improved imbalance in the distribution of the COPD burden among countries with varying SDI levels.

### Predictive analysis on COPD burden to 2045

**Figure 6:**
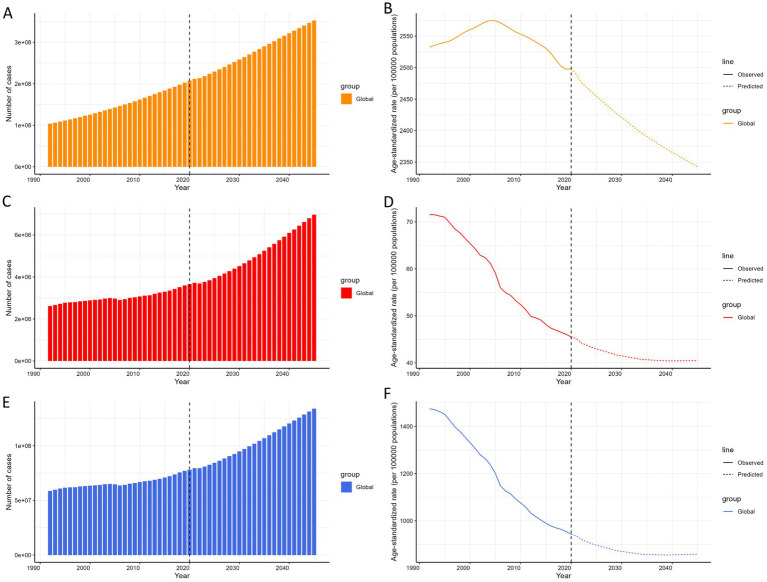
**(A)** The predicted case number of prevalence to 2045. **(B)** The predicted ASR of prevalence from 2045. **(C)** The predicted case number of mortality to 2045. **(D)** The predicted ASR of mortality to 2045. **(E)** The predicted case number of DALYs to 2045. **(F)** The predicted ASR of DALYs to 2045; of COPD globally. ASR, age-standardized rate; DALYs, disability-adjusted life-years; COPD, chronic obstructive pulmonary disease.

The projected case numbers and ASRs for COPD prevalence, mortality, and DALYs through 2045 are presented in Figure 6. Globally, the number of cases for prevalence, mortality, and DALYs is predicted to continue increasing, while the ASRs for prevalence, mortality, and DALYs are expected to decline annually over the same period. Detailed projections for the case numbers and ASRs of prevalence, mortality, and DALYs are provided in Supplementary Table S7.

## Discussion

This study offers the most up-to-date insights into the global, regional, and national burden of COPD, analyzing prevalence, mortality, and DALYs from 1990 to 2021. It integrates comprehensive analyses, including trends, decomposition, inequality, and future projections. Although ASRs for prevalence, mortality, and DALYs have generally declined, the absolute numbers of cases, deaths, and DALYs have consistently increased over time. Decomposition analyses indicate that population growth and aging have been the primary drivers of the rising COPD burden, while improvements in epidemiological factors have contributed to its decline. Inequality analyses reveal that SDI-related disparities have widened, with a growing concentration of the COPD burden in higher SDI countries. Projections suggest that although ASRs for prevalence, mortality, and DALYs will continue to decline, the absolute number of cases will keep rising. This highlights the ongoing and escalating challenge of managing COPD globally in the coming decades.

In 2021, global COPD burden included 213 million prevalent cases, 3.71 million deaths, and 79.78 million DALYs. East Asia had the highest number of prevalent cases and deaths, while South Asia had the largest number of DALYs. High-income North America exhibited the highest age-standardized prevalence rates, and Oceania had the highest age-standardized mortality and DALYs rates. Notably, regions with the most significant increases in COPD burden also warrant attention. For example, the Caribbean saw the largest rise in age-standardized prevalence and DALYs, while High-income North America experienced the largest increase in age-standardized mortality.

At the national level, variations in the COPD burden and trends emphasize the need for flexible, context-specific health policies. China and India, the two most populous countries, have the highest absolute numbers of prevalent cases, deaths, and DALYs. While their ASRs are not the highest globally, their large populations contribute to a persistently high COPD burden. In China, the significant COPD burden is primarily driven by severe air pollution linked to rapid urbanization and industrialization (19). Additionally, the country’s high smoking prevalence exacerbates the issue. China had 308 million adult smokers as of 2018, with 50% of adult males affected. Furthermore, more than 700 million people in China are exposed to secondhand smoke (20).

In economically underdeveloped countries such as India, indoor air pollution from the use of solid fuels is a major contributor to the COPD burden (13). Due to limited access to cleaner cooking fuels, firewood, dung, and biomass are commonly used as primary energy sources, exposing a significant portion of the population to harmful indoor air pollution (21). Women, in particular, are disproportionately affected due to their longer exposure times during household activities (22). This exposure has contributed to a higher increase in COPD mortality rates among women than men (23).

In contrast, the United States, as a highly developed country, exhibits the highest age-standardized prevalence rates of COPD. This is largely due to rapid population aging, which has led to an increase in the age-specific prevalence of COPD (24). The U.S. also benefits from advanced chronic disease management and extensive screening programs (25), which likely contribute to its higher reported prevalence rates compared to other nations. Despite these advances, the high prevalence of COPD in the U.S. highlights the ongoing challenge of managing chronic diseases in aging populations. On the other hand, Papua New Guinea, with its limited healthcare resources, faces considerable challenges in the timely diagnosis and treatment of COPD (26). The widespread use of solid fuels for cooking, combined with high levels of outdoor air pollution, exacerbates respiratory conditions in the region (27). Strengthening public health initiatives, improving access to healthcare, and raising awareness about COPD in vulnerable regions are essential for reducing the disease burden and improving health outcomes.

Age, period, and birth cohort effects are key frameworks for understanding how individuals and societies evolve over time (28). The relative risk of COPD prevalence and mortality increases with age, peaking in the 95–99 age group. Notably, the relative risk surpasses 1 in the 60–64 age group for both males and females, highlighting the critical need for targeted prevention, management, and treatment interventions for individuals aged 60 and older. Moreover, for males aged 55–59, the relative risk also exceeds 1, underlining the importance of advancing COPD health management measures for men. In addition, factors such as smoking, air pollution, and occupational exposures—many of which begin to affect individuals early in life—highlight the importance of identifying precursor conditions to COPD. These conditions offer critical opportunities for prevention, early diagnosis, and timely treatment (29).

The period effect shows that relative risks for both prevalence and mortality have risen over time, reflecting changes in medical technology, economic conditions, and cultural factors. Advances in medical diagnostics, such as spirometry, lung diffusion tests, chest radiographs, and arterial blood gas tests, have also contributed to the observed trends by improving the ability to diagnose and track the disease (30). Regarding birth cohort effects, the relative risk for COPD prevalence and mortality decreases in successive cohorts, suggesting that individuals born later are at a lower risk compared to earlier cohorts. This trend may reflect the cumulative effect of risk factors, such as smoking and air pollution, which were more prevalent in earlier generations. In contrast, later cohorts have benefited from improved access to health education, smoking cessation programs, and advances in healthcare, which have helped reduce overall COPD risk (10). This cohort-based decline in risk underscores the importance of continuing public health efforts and improving healthcare accessibility to mitigate COPD risk in future generations.

The observed decline in ASR post-2004 likely reflects cumulative public health efforts. For instance, 168 countries adopted the WHO FCTC by 2008, reducing smoking rates by 2.5% globally between 2000–2015 (31). Concurrently, China’s air pollution mitigation policies lowered PM2.5 levels by 30% in key industrial regions from 2005–2015 (32), directly impacting COPD risk. Improved diagnostic consistency through GOLD guideline updates (2001, 2006) may also have reduced misclassification bias in later years (33).

By quantifying inequalities in the burden of COPD across countries with different SDI gradients, this study clarifies the distribution patterns of COPD burden and identifies countries that require enhanced prevention and control measures (10). While it is often assumed that higher SDI levels correlate with greater access to healthcare and more effective healthcare systems—leading to a reduced disease burden—our findings reveal that COPD burden is concentrated in high-SDI countries. Moreover, health inequalities linked to socio-economic disparities have increased over time, echoing conclusions from previous studies (34). In the early years, the burden was more evenly distributed, but it has increasingly concentrated in high-income nations. However, as economies and populations grow, developing countries, particularly middle-income ones, are expected to bear an increasing share of the burden (35). Countries with lower SDI levels remain more vulnerable to COPD, as indicated by previous research (5).

The unequal concentration of COPD in high-SDI countries is primarily attributed to two factors: (1) an aging population, which is inevitable in higher-SDI countries (36), and (2) the incurable nature of COPD, which results in higher prevalence rates despite the advanced diagnostic and therapeutic capacities in these nations (37). In contrast, low- and middle-SDI countries face underdiagnosis and underreporting issues, largely due to limited awareness among local healthcare providers, which leads to an underestimation of actual COPD prevalence (38). To address health inequalities, targeted policies should be tailored to the specific needs of countries at different SDI levels. These disparities call for context-specific interventions:

- High-SDI regions should prioritize smoking cessation programs and air quality regulations to mitigate aging-related risks (39).
- Low/middle-SDI regions require scaled-up access to spirometry, affordable bronchodilators, and clean cooking technologies to reduce biomass fuel exposure (40).
- Global partnerships must address diagnostic gaps; for example, task-shifting spirometry to community health workers in rural India could improve early detection (23).

Furthermore, strengthening primary care infrastructure in LMICs is critical. In Papua New Guinea, where 80% of COPD cases remain undiagnosed, integrating respiratory screening into existing HIV/TB programs could leverage underutilized resources (41). Similarly, China’s “Healthy Aging 2030” initiative provides a model for COPD management in aging populations through telemedicine and community rehabilitation (42).

Projections indicate that by 2045, the ASRs for COPD prevalence, mortality, and DALYs will decline annually, while the absolute number of cases will continue to rise. Decomposition analysis attributes the increase in COPD cases over the past 32 years to aging and population growth. Similarly, demographic changes, including ongoing population growth and accelerated aging, are expected to drive future rises in COPD cases. The United Nations projects that the proportion of people aged 65 or older will increase from one in eleven in 2019 to one in six by 2050 (43). To address these shifts, countries must enhance healthcare systems with a focus on aging populations and equitable resource allocation. Tackling the challenges posed by aging and population growth is crucial to effectively mitigating the global burden of COPD.

Several limitations of this study should be acknowledged. First, in less developed countries, lower-quality healthcare systems often result in misdiagnosis, underdiagnosis, and incomplete documentation, leading to an underestimation of COPD cases in the GBD. Second, the raw data in the GBD are derived from multiple countries, leading to variability in data quality. Differences in COPD diagnostic criteria may result in disease misclassification, complicating cross-population comparisons. While data cleaning, calibration, and advanced statistical modeling techniques have been employed to mitigate these issues, reliance on modeled data remains a limitation (44–46). Despite these limitations, the study’s strengths lie in its comprehensive analyses, which significantly advance our understanding of COPD epidemiology and provide a solid foundation for global public health policies and resource allocation.

In conclusion, COPD remains a significant global public health challenge, with considerable variation in prevalence, mortality, and DALYs across countries. Although age-standardized rates of COPD have declined from 1990 to 2021, the rise in absolute cases highlights the impact of population growth and aging, which are expected to drive further increases in the coming decades. Countries with higher SDI scores are bearing a share of the burden, and inequalities between nations are widening. These findings emphasize the persistent challenges in COPD management, particularly the growing number of cases and the unequal distribution of its burden. To address these issues, it is critical to develop targeted health policies, optimize resource allocation, and implement adaptive strategies tailored to the needs of individual countries. Such measures will help reduce health disparities and improve global COPD management.

## Data Availability

Publicly available datasets were analyzed in this study. This data can be found at: https://vizhub.healthdata.org/gbd-results/.
