# Supplementary material for: Chronic obstructive pulmonary disease across three decades: trends, inequalities, and projections from the Global Burden of Disease Study 2021
Source: Front Med (Lausanne). 2025 Mar 24;12:1564878. doi: 10.3389/fmed.2025.1564878 (PMC11973060; doi:10.3389/fmed.2025.1564878)

Figure S1 Comparison of global ASRs of prevalence, mortality, and DALYs from 1990 to 2021 by sexes.


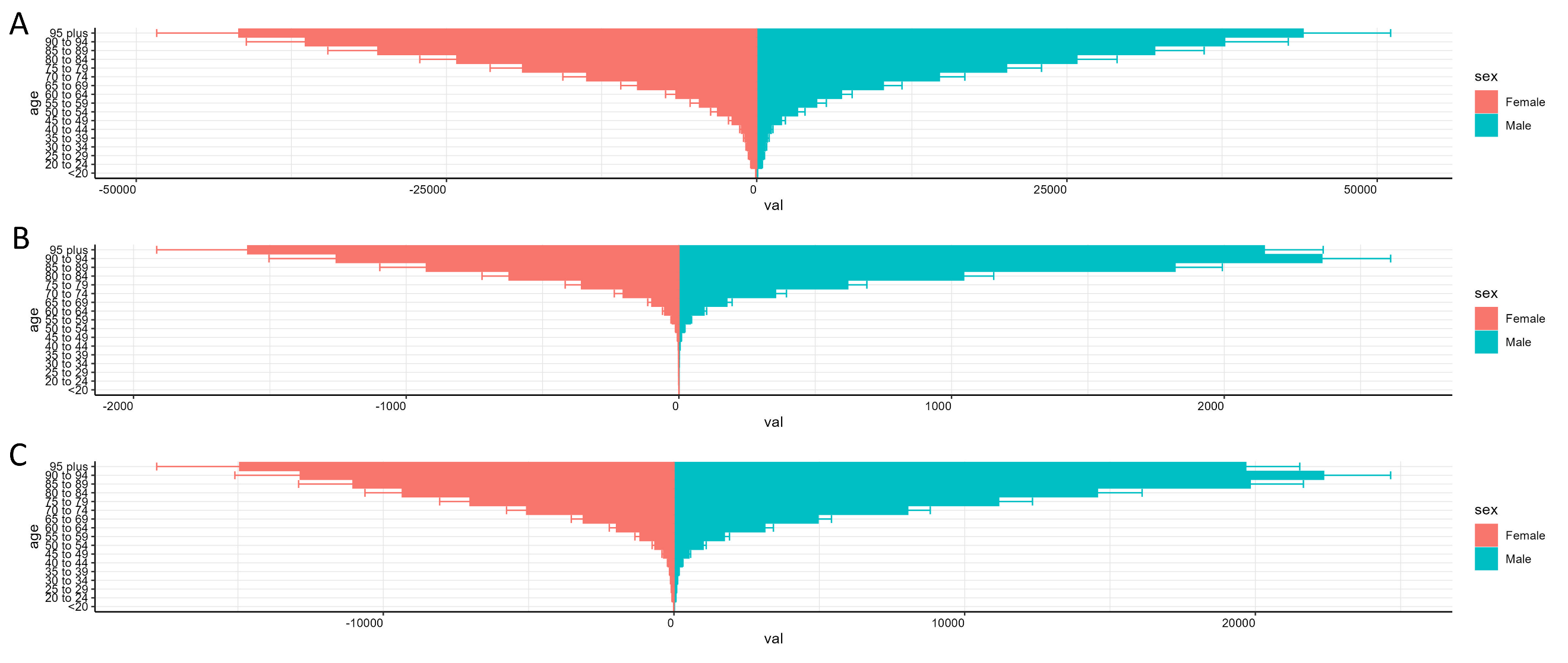


Figure S2 The effects of age, period, and birth cohort on the relative risk of COPD prevalence (A) and death (B) by sexes.


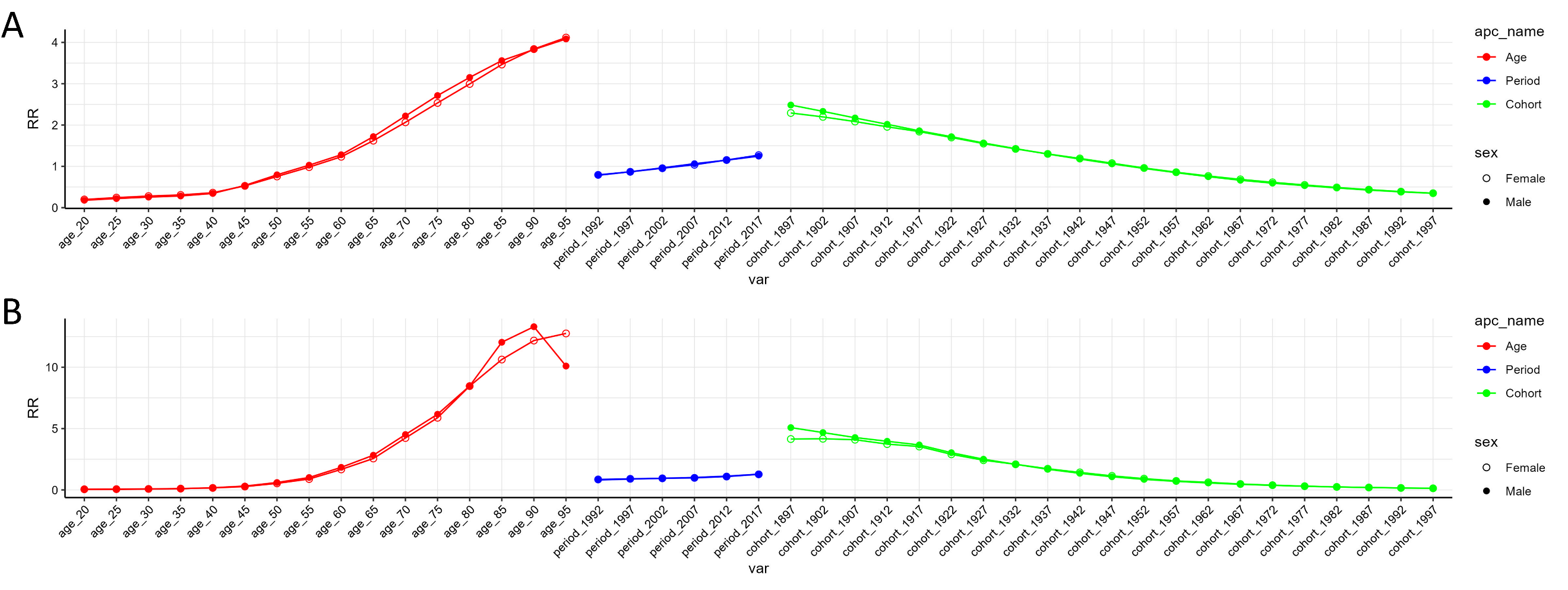


Figure S3 Changes in incidence of COPD according to aging, population growth and epidemiological change from (A)1990 to 2004; (B) 2004 to 2021 at global level by SDI quintile and by sexes.


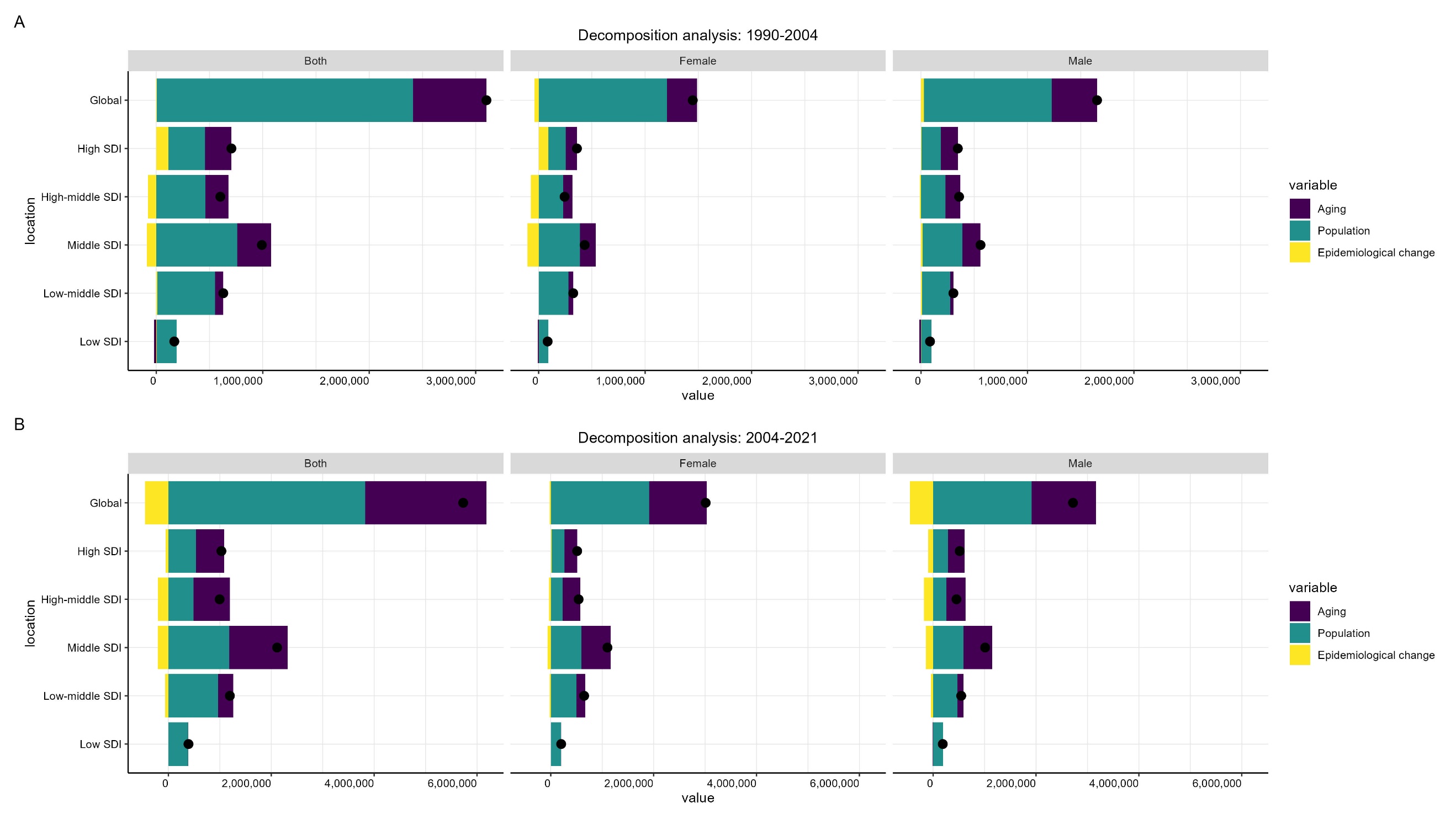

Supplement: Supplementary file 1 [file Supplementary_file_1.docx]
