# Supplementary material for: Chronic obstructive pulmonary disease across three decades: trends, inequalities, and projections from the Global Burden of Disease Study 2021
Source: Front Med (Lausanne). 2025 Mar 24;12:1564878. doi: 10.3389/fmed.2025.1564878 (PMC11973060; doi:10.3389/fmed.2025.1564878)
Supplement: Supplementary file 2 [file Table_1.docx]

Table S1 The case number and ASR of prevalence of COPD in 1990 and 2021 for both sexes by 204 countries and territories, with EAPC from 1990 to 2021.

| Location | 1990 | | 2021 | | EAPC(95%CI)  1990-2021 |
| --- | --- | --- | --- | --- | --- |
|  | Number(95%UI) | ASR(95%UI) | Number(95%UI) | ASR(95%UI) |  |
| Afghanistan | 160618.02(143485.29~179650.99) | 2385.47(2138.28~2655.11) | 293963.54(265452.59~325421.03) | 2590.12(2315.15~2913.83) | 0.17(0.14 ~ 0.19) |
| Albania | 48672.21(42818.52~55089.75) | 2319.36(2036.62~2638.44) | 100990.98(87982.82~115685.67) | 2389.58(2092.08~2724.78) | 0.14(0.06 ~ 0.23) |
| Algeria | 282043.58(250585.02~318828.4) | 2184.97(1917.62~2492.49) | 928242.61(819663.15~1038356.83) | 2643.64(2331.69~2954.44) | 0.62(0.57 ~ 0.68) |
| American Samoa | 546.5(494.63~608.29) | 2275.87(2017.61~2565.99) | 895.91(795.93~1007.4) | 1975.32(1744.82~2226.52) | -0.52(-0.54 ~ -0.5) |
| Andorra | 1621.77(1457.82~1799.55) | 2863.66(2569.41~3171.15) | 4031.03(3596.29~4534.58) | 2655.23(2387.68~2982.29) | -0.28(-0.31 ~ -0.26) |
| Angola | 77618.48(68356.88~87200.53) | 1766.25(1567.65~2007.82) | 216940(189977.89~243494.28) | 1672.11(1444.04~1899.12) | -0.3(-0.34 ~ -0.25) |
| Antigua and Barbuda | 558.5(476.31~641.41) | 1011.32(867.97~1165.68) | 1356.46(1163.96~1556.63) | 1325.13(1134.42~1515.79) | 0.87(0.82 ~ 0.92) |
| Argentina | 508302.61(439231.76~575673.32) | 1603.99(1386.47~1818.58) | 892476.05(798704.41~999970.62) | 1601.3(1436.49~1793.4) | 0.05(-0.04 ~ 0.14) |
| Armenia | 64153.87(57734.22~72485.96) | 2390.25(2132.91~2696.99) | 100139(89041.89~113173.59) | 2370.28(2110.39~2680.12) | -0.01(-0.04 ~ 0.02) |
| Australia | 405539.26(356046.41~448131.9) | 2076.5(1826.07~2292.52) | 706604.79(624380.41~818768.81) | 1562.13(1386.02~1789.86) | -0.92(-1.01 ~ -0.84) |
| Austria | 327755.87(297363.94~358871.82) | 2860.19(2600.49~3116.52) | 511218.58(457335.06~558680.5) | 2891.94(2593.55~3153.57) | 0(-0.12 ~ 0.13) |
| Azerbaijan | 113124.01(100888.27~127067.13) | 2205.22(1949.45~2500.73) | 216263.68(190401.06~245357.58) | 2246.65(1967.69~2555.5) | 0.03(-0.03 ~ 0.08) |
| Bahamas | 1816.35(1587.54~2086.32) | 1107.27(963.84~1280.03) | 5236.36(4613.77~5896.74) | 1320.88(1167.46~1488.52) | 0.58(0.52 ~ 0.64) |
| Bahrain | 5149.44(4639.74~5737.69) | 2462.86(2192.99~2778.89) | 23865.36(21039.3~27392.51) | 2547.63(2251.17~2882.69) | 0(-0.05 ~ 0.04) |
| Bangladesh | 1443424.06(1328408.45~1576970.32) | 2914.65(2671.74~3206.63) | 4020093.9(3629428.13~4477582.38) | 2956.2(2669.12~3284.81) | 0.11(0.01 ~ 0.21) |
| Barbados | 3032.59(2614.85~3500.33) | 1043.02(904.91~1211.5) | 6436.92(5548.59~7310.6) | 1305.44(1133.26~1483.27) | 0.72(0.68 ~ 0.76) |
| Belarus | 310190.62(278078.27~346601.28) | 2460.8(2209.37~2744.35) | 312912.34(275571.48~354243.67) | 2046.92(1814.29~2305.15) | -0.71(-0.77 ~ -0.65) |
| Belgium | 369719.05(332899.79~410612.95) | 2495.23(2254.28~2763.21) | 560791.94(504009.09~628348.68) | 2469.8(2225.42~2749.37) | -0.04(-0.09 ~ 0.02) |
| Belize | 1462.95(1288.14~1627.52) | 1454.85(1279.95~1642.28) | 5399.03(4748.11~6049.33) | 1746.82(1525.5~1967.8) | 0.66(0.64 ~ 0.68) |
| Benin | 37179.35(32985.96~41832.54) | 1626.94(1440.21~1848.18) | 105518.5(93284.74~117104.54) | 1705.2(1495.85~1929.75) | 0.15(0.1 ~ 0.2) |
| Bermuda | 800.27(693.88~907.44) | 1292.2(1115.61~1478.85) | 1999.87(1710.86~2289.64) | 1521.79(1309.73~1727.1) | 0.46(0.38 ~ 0.55) |
| Bhutan | 7502.51(6770.74~8133.51) | 2969.93(2681.69~3264.38) | 16900.7(15257.96~18836.41) | 2772.6(2481.15~3092.09) | -0.27(-0.29 ~ -0.25) |
| Bolivia (Plurinational State of) | 62269.39(54941.51~71235.26) | 1900.83(1672.36~2171.32) | 173920.29(152345.67~197808.88) | 1967.35(1724.58~2233.81) | 0.1(0.07 ~ 0.14) |
| Bosnia and Herzegovina | 101028.37(90095.89~114072.03) | 2520.04(2246.7~2839.35) | 154732.46(137494~174491.95) | 2584.7(2311.27~2896.99) | 0.15(0.12 ~ 0.17) |
| Botswana | 11969.81(10634.12~13455.09) | 1976.36(1749.2~2219.02) | 31547.64(27926~35450.47) | 2016.93(1774.24~2287.47) | 0.03(-0.05 ~ 0.11) |
| Brazil | 2314910.69(2065609.01~2578531.26) | 2583.2(2281.62~2885.54) | 6314694.44(5587560.53~7077063.89) | 2555.99(2259.52~2860.37) | -0.17(-0.21 ~ -0.12) |
| Brunei Darussalam | 2375.97(2125.13~2638.87) | 2272.34(2023.69~2530.72) | 5191.61(4569.51~5809.96) | 1662.05(1457.5~1879.28) | -1.15(-1.2 ~ -1.11) |
| Bulgaria | 289258.14(255738.08~322960.09) | 2475.06(2181.23~2745.96) | 334019.46(289512.28~380136.61) | 2453.21(2164.24~2774.09) | -0.03(-0.06 ~ 0) |
| Burkina Faso | 70797.85(62356.33~79422.38) | 1489.91(1304.23~1675.86) | 172987.34(153466.19~193704.52) | 1608.13(1425.7~1828.58) | 0.24(0.19 ~ 0.28) |
| Burundi | 45665.94(40617.23~51278.69) | 1715.01(1520.02~1945.59) | 109735.86(97673.97~121235.79) | 1878.33(1651.01~2110.58) | 0.15(0.09 ~ 0.22) |
| Cabo Verde | 2308.1(2026.92~2608.04) | 985.59(864.7~1112.81) | 4804.87(4201.15~5403.46) | 1001.29(872.55~1138.04) | 0.05(-0.06 ~ 0.15) |
| Cambodia | 103445.91(91844.8~115474.07) | 2167.17(1904.7~2447.99) | 272673.51(243081.35~308825.27) | 2230.67(1969.93~2529.52) | 0.07(0.03 ~ 0.1) |
| Cameroon | 77952.58(68249.68~87434.36) | 1512.12(1324.99~1725.19) | 239814.74(213803.75~269208.97) | 1574.02(1383.41~1800.29) | 0.08(0.02 ~ 0.14) |
| Canada | 676615.2(587085.07~773755.5) | 2076.16(1804.27~2371.24) | 1522991.43(1358067.57~1700360.2) | 2098.38(1880.9~2351.54) | -0.2(-0.49 ~ 0.09) |
| Central African Republic | 24219.28(21281.32~27224.54) | 1941.74(1708.22~2209.27) | 52024.05(45816.86~57955.14) | 2145.27(1877.74~2435.77) | 0.29(0.26 ~ 0.32) |
| Chad | 52713.03(46454.74~59152.96) | 1700.76(1485.88~1923.74) | 129102.43(114268.56~144157.95) | 1857.59(1625~2113.48) | 0.29(0.23 ~ 0.35) |
| Chile | 142702.07(124910.53~161754.36) | 1425.55(1247.9~1622.34) | 310100.26(269434.15~353782.85) | 1213.7(1060.34~1388.75) | -0.57(-0.6 ~ -0.54) |
| China | 23144968.76(20878168.63~25434630.64) | 2761.81(2498.94~3033.6) | 50588429.3(44975892.81~57116835.17) | 2499.35(2236.21~2793.29) | -0.33(-0.37 ~ -0.29) |
| Colombia | 408589.39(373300.54~452515.02) | 2273.38(2069.07~2538.12) | 1306282.89(1176060.55~1451413.03) | 2340.91(2106.1~2604.23) | -0.09(-0.17 ~ -0.02) |
| Comoros | 3373.17(2973.24~3774.56) | 1510.75(1332.76~1690.44) | 7791.46(6800.09~8782.79) | 1492.5(1299.98~1696.45) | -0.13(-0.19 ~ -0.06) |
| Congo | 18871.74(16658.56~21140.36) | 1637.74(1442.4~1857.02) | 49376.33(43371.41~55552.48) | 1716.96(1501.89~1954.86) | 0.09(0.03 ~ 0.16) |
| Cook Islands | 261.33(230.66~294.28) | 2082.01(1809.15~2349.32) | 447.73(378.99~512.68) | 1821.43(1557.93~2077.04) | -0.55(-0.59 ~ -0.51) |
| Costa Rica | 34485.91(30529.5~38954.39) | 1918.16(1683.24~2172.08) | 115582.72(100152.8~129868.6) | 2098.78(1814.84~2368.32) | 0.22(0.17 ~ 0.26) |
| Coted'Ivoire | 80831.29(71556.16~90415.92) | 1558.16(1377.48~1762.65) | 230232.07(204519.02~259208.93) | 1691.67(1489.89~1934.8) | 0.31(0.24 ~ 0.38) |
| Croatia | 131328.01(115133.7~149023.21) | 2238.98(1958.16~2529.54) | 222941.35(199359.8~246696.37) | 2553.27(2289.68~2833.69) | 0.5(0.47 ~ 0.52) |
| Cuba | 176802.75(155112.67~202467.26) | 1711.73(1497.19~1960.52) | 409597.99(365089.04~455411.12) | 2139.36(1919.69~2374.51) | 0.76(0.66 ~ 0.86) |
| Cyprus | 20880.32(18616.47~23832.21) | 2671.67(2387.23~3013.31) | 50059.59(44353.03~56084.21) | 2496.35(2221.78~2781.09) | -0.22(-0.25 ~ -0.19) |
| Czechia | 280445.78(243730.51~320222.55) | 2107.39(1836.26~2393.4) | 516786.48(462606.72~566688.1) | 2498.69(2264.56~2734.86) | 0.58(0.56 ~ 0.6) |
| Democratic People's Republic of Korea | 495175.71(455923.88~538707.91) | 3166.45(2930.4~3426.85) | 879586.61(791149.15~981320.44) | 2757.5(2487.07~3066.78) | -0.48(-0.53 ~ -0.43) |
| Democratic Republic of the Congo | 298041.76(261655.64~332270.15) | 1756.13(1554.01~1980.06) | 807959.01(717254.36~913626.8) | 2029.81(1800.47~2308.63) | 0.45(0.43 ~ 0.47) |
| Denmark | 229515.64(205354.56~253808.3) | 2941.77(2648.48~3252.83) | 310720.04(275454.26~344044.28) | 2732.96(2448.7~3009.8) | -0.37(-0.42 ~ -0.31) |
| Djibouti | 2359.25(2055.99~2663.75) | 1318.58(1146.93~1488.6) | 10503.92(9075.49~11843.85) | 1412.18(1230.01~1602.95) | 0.11(-0.03 ~ 0.26) |
| Dominica | 762.72(662.21~868.3) | 1282.53(1121.49~1462.43) | 1176.68(1004.95~1352.34) | 1451.22(1241.72~1668.9) | 0.4(0.39 ~ 0.42) |
| Dominican Republic | 53627.59(46965.79~60312.81) | 1346.75(1182.26~1524.59) | 169699.04(147743.97~193588.21) | 1680.84(1452.7~1916.87) | 0.77(0.75 ~ 0.8) |
| Ecuador | 87818.3(76335.18~100000.81) | 1588.31(1376.19~1817.78) | 281618.66(246354.96~320213.21) | 1737.48(1521.87~1977.78) | 0.23(0.19 ~ 0.27) |
| Egypt | 542941.51(480464.02~617189) | 1868.22(1644.89~2143.63) | 1555374.12(1364099.22~1775225.21) | 2472.66(2161.71~2806.85) | 0.86(0.83 ~ 0.88) |
| El Salvador | 51615.09(45142.29~58661.18) | 1670.1(1452.44~1901.53) | 120349.21(104289.62~137641.89) | 1855.16(1599.69~2125.36) | 0.27(0.24 ~ 0.31) |
| Equatorial Guinea | 3765.21(3307.03~4243.76) | 1835.02(1614.19~2079.1) | 10267.14(8986.2~11530.81) | 1732.08(1522.72~1979.46) | -0.2(-0.35 ~ -0.05) |
| Eritrea | 21923.08(19300.7~24424.46) | 1529.39(1341.13~1714.28) | 51716.32(45557.79~58726.04) | 1543.62(1344.22~1764.93) | -0.06(-0.13 ~ 0.02) |
| Estonia | 26717.26(23698.25~30153.71) | 1352.47(1200.44~1516.22) | 39751.07(35068.21~44930.31) | 1532.4(1367.82~1728.88) | 0.71(0.62 ~ 0.8) |
| Eswatini | 6262.89(5484.9~7063.38) | 1932.31(1697.17~2193.9) | 11741.73(10401.91~13112.68) | 1921.59(1707.84~2173.55) | -0.09(-0.16 ~ -0.02) |
| Ethiopia | 412945.4(367394.34~459903.03) | 1821(1601.44~2053.96) | 890979.26(793351.82~987246.84) | 1715.27(1509.18~1918.44) | -0.3(-0.36 ~ -0.23) |
| Fiji | 7946.16(7107.85~8861.44) | 2100.68(1843.83~2381.55) | 12013.69(10576.51~13794.98) | 1655.84(1446.42~1890.83) | -0.83(-0.87 ~ -0.79) |
| Finland | 145714.72(133320.51~160065) | 2125.14(1943.43~2326.77) | 256967.66(224690.91~292252.34) | 2133.03(1886.93~2393.42) | -0.14(-0.21 ~ -0.07) |
| France | 1824099.74(1609424.28~2051056.59) | 2255.01(2001.12~2522.03) | 2969242.85(2646069.68~3337983.2) | 2182.53(1950.2~2438.77) | -0.06(-0.12 ~ -0.01) |
| Gabon | 8187.05(7106.69~9259.33) | 1391.46(1210.2~1575.16) | 16523.85(14379.92~18762.24) | 1530.9(1330.61~1767.76) | 0.29(0.18 ~ 0.39) |
| Gambia | 7017.18(6214.16~7881.25) | 1629.89(1430.6~1848.06) | 20616.13(18392.7~22929.13) | 1778.31(1574.41~2005.21) | 0.3(0.25 ~ 0.36) |
| Georgia | 107115.15(93931.01~121949.91) | 1782.14(1561.27~2030.01) | 121326.48(105831.53~137326.08) | 2091.88(1844.96~2352.67) | 0.55(0.5 ~ 0.61) |
| Germany | 3540084.22(3171555.91~3962544.9) | 2873.29(2587.82~3205.01) | 5218529.28(4717214.53~5793017.89) | 2759.62(2508.82~3043.39) | -0.11(-0.21 ~ 0) |
| Ghana | 105794.38(93383.51~118883.54) | 1438.67(1260.06~1614.25) | 320571.81(285498.19~355226.69) | 1672.26(1471.4~1876.37) | 0.54(0.49 ~ 0.6) |
| Greece | 373197.38(326400.17~423494.42) | 2563.04(2260.52~2891.06) | 609189.7(546308.29~683100.14) | 2575.73(2325.26~2864.41) | 0(-0.03 ~ 0.02) |
| Greenland | 875.29(789.96~968.54) | 2728.23(2491.3~3005.96) | 1677.21(1516.25~1867.77) | 2542.79(2306.06~2822.86) | -0.24(-0.26 ~ -0.21) |
| Grenada | 912.59(790.7~1047.02) | 1241.39(1076.4~1414.06) | 1630.18(1424.57~1831.3) | 1482.2(1299.19~1658.69) | 0.53(0.48 ~ 0.58) |
| Guam | 1431.22(1253.24~1613.15) | 1821.17(1564.65~2098.26) | 3391.82(2875.42~3841.49) | 1623.56(1387.66~1836.26) | -0.39(-0.43 ~ -0.35) |
| Guatemala | 63394.89(55643.8~71733.64) | 1803.39(1584.05~2040.03) | 209544.52(186412.16~236785.77) | 1906.12(1694.12~2164.38) | 0.06(0 ~ 0.12) |
| Guinea | 57190.17(50478.53~64851.26) | 1618.35(1424.4~1837.1) | 119666.59(106166.63~132601.46) | 1842.2(1625.56~2067.62) | 0.42(0.37 ~ 0.48) |
| Guinea-Bissau | 7793.32(6933.11~8702.29) | 1666.21(1477.1~1877.54) | 16064.59(14307.97~17918.27) | 1757.8(1541.23~2001.48) | 0.18(0.1 ~ 0.27) |
| Guyana | 4788.24(4151.14~5429.9) | 1163.95(1006.46~1333.4) | 8911.75(7696.87~10104.92) | 1411.33(1215.33~1605.97) | 0.55(0.51 ~ 0.58) |
| Haiti | 59655.05(51816.61~67732.4) | 1777.03(1555.84~2034.88) | 142144.53(125213.22~159936.36) | 1909.4(1687.77~2174.25) | 0.15(0.1 ~ 0.2) |
| Honduras | 45002.91(39615.4~50566.49) | 2128.56(1864.34~2401.8) | 157513.4(139565.15~179516.68) | 2521.81(2240.28~2870.89) | 0.51(0.47 ~ 0.56) |
| Hungary | 361769.65(319999.81~408925.1) | 2561.16(2274.07~2884.03) | 548104.39(491546.81~598032.18) | 2933.74(2673.56~3185.9) | 0.49(0.46 ~ 0.51) |
| Iceland | 8823.24(7938.29~9710.93) | 3092.11(2792.54~3400.82) | 15732(14101.88~17342.04) | 2769.55(2492.45~3053.54) | -0.48(-0.61 ~ -0.34) |
| India | 13636911.06(12310070.17~14829187.59) | 2967.33(2676~3242.41) | 35819854.91(32396904.15~39076078.78) | 3067.86(2782.53~3347.91) | 0.13(0.11 ~ 0.15) |
| Indonesia | 2052840.09(1816777.26~2277611.73) | 1972.09(1735.91~2218.39) | 5080725.22(4434330.15~5713205.36) | 2204.76(1930.54~2481.68) | 0.32(0.3 ~ 0.33) |
| Iran (Islamic Republic of) | 443959.57(391419.27~499855.88) | 1611.84(1415.39~1817.15) | 1586139.27(1396410.6~1781229.38) | 2054.63(1798.37~2321.91) | 0.76(0.73 ~ 0.78) |
| Iraq | 150580.18(131954.84~171110.58) | 1675.15(1466.3~1924.39) | 494469.97(435909.92~559676.31) | 2003.35(1756.16~2283.54) | 0.6(0.55 ~ 0.64) |
| Ireland | 111292.98(99063.63~122397.91) | 2785.8(2495.32~3060.54) | 191319.43(172627.27~213047.63) | 2466.78(2233.2~2735.65) | -0.42(-0.44 ~ -0.41) |
| Israel | 122779.16(111012.83~136840.46) | 2592.27(2345.95~2884.18) | 283970.33(250022.58~317251.13) | 2340.1(2084.01~2602.78) | -0.34(-0.37 ~ -0.31) |
| Italy | 1933787.76(1682933.04~2200329.09) | 2255.74(1980.04~2537.61) | 3174977.89(2745407.6~3585532.42) | 2159.42(1891.01~2433.84) | -0.17(-0.21 ~ -0.13) |
| Jamaica | 27179.31(23733.42~30487.16) | 1481.86(1299.39~1657.33) | 52551.82(47134.44~58583.53) | 1653.88(1478.44~1848.05) | 0.33(0.27 ~ 0.4) |
| Japan | 2739502.26(2364839.26~3145669.39) | 1674.2(1455.45~1916.56) | 5114116.64(4309205.53~6010382.11) | 1285.53(1092.96~1492.23) | -0.75(-0.83 ~ -0.68) |
| Jordan | 30879.74(27434.78~34650.65) | 2023.15(1788.86~2299.83) | 165854.29(145994.23~186678.37) | 2153.03(1877.41~2442.68) | 0.17(0.15 ~ 0.19) |
| Kazakhstan | 317627.89(285522.78~357488.14) | 2494(2232.83~2826.06) | 474247.13(429214.18~532108.57) | 2778.16(2508.07~3109.01) | 0.47(0.35 ~ 0.59) |
| Kenya | 139126.52(121780.31~155390.84) | 1415.15(1233.59~1594.96) | 403346.96(352451.97~452161.1) | 1528.53(1330.32~1716.06) | 0.13(0.08 ~ 0.18) |
| Kiribati | 909.27(820.24~1013.5) | 2372.05(2093.17~2657.2) | 1668.5(1493.97~1876.6) | 2277.89(1999.04~2574.47) | -0.1(-0.12 ~ -0.08) |
| Kuwait | 13234.07(11531.47~15028.4) | 1648.36(1447.33~1890.08) | 66467.33(58682.88~74860.72) | 2006.85(1773.08~2282.36) | 0.64(0.62 ~ 0.66) |
| Kyrgyzstan | 89308.74(82238.41~97863.64) | 2970.96(2718.63~3276.68) | 125202.34(111803.3~140538.4) | 2653.88(2332.34~2976.49) | -0.5(-0.55 ~ -0.45) |
| Lao People's Democratic Republic | 52839.95(47228.16~58391.59) | 2487.21(2219.05~2778.5) | 113895.91(102552.49~127023.55) | 2401.41(2141.97~2705.24) | -0.2(-0.24 ~ -0.15) |
| Latvia | 62559.94(54669.4~71290.97) | 1813.04(1591.83~2056.14) | 67917.24(59522.12~76700.02) | 1801.32(1589.74~2021.76) | -0.05(-0.16 ~ 0.05) |
| Lebanon | 41434.24(36610.61~46883.74) | 1898.4(1677.37~2152.02) | 153048.2(132931.66~174115.17) | 2481.23(2159.62~2823.22) | 1.01(0.95 ~ 1.06) |
| Lesotho | 18890.7(16788.79~21344.32) | 2155.66(1913.98~2453.65) | 26389.76(23470.8~29591.7) | 2334.74(2076.67~2646.7) | 0.23(0.17 ~ 0.29) |
| Liberia | 18150.1(16021.26~20457.49) | 1425.92(1259.63~1604.91) | 43723.86(38564.58~49060.76) | 1718.18(1498.14~1946.85) | 0.52(0.48 ~ 0.57) |
| Libya | 37889.82(33708.53~42581.41) | 1790.05(1575.96~2024.96) | 121288.92(107865.79~136197.33) | 2224.56(1971.25~2518.3) | 0.76(0.74 ~ 0.79) |
| Lithuania | 94731.05(83519.15~106897.96) | 2137.81(1890.65~2408.07) | 103040.88(91249.89~116814.19) | 1875.04(1673.21~2112.08) | -0.54(-0.61 ~ -0.47) |
| Luxembourg | 14447.73(13074.03~15948.42) | 2726.23(2474.42~3004.15) | 26887.99(24038.89~29849.95) | 2570.61(2309.6~2854.1) | -0.23(-0.29 ~ -0.17) |
| Madagascar | 93791.06(82716.21~104591.99) | 1614.63(1408.48~1813.26) | 248160.41(221119.58~277578.19) | 1857.98(1648.41~2120.94) | 0.35(0.31 ~ 0.39) |
| Malawi | 58859.65(51852.57~65887.03) | 1299.1(1145.82~1461.97) | 136848.27(120542.72~154114.19) | 1530.87(1352.11~1728.65) | 0.48(0.43 ~ 0.52) |
| Malaysia | 171658.47(151357.48~193929.26) | 1737.55(1518.35~1965.95) | 499394.92(433574.26~562409.68) | 1784.36(1540.75~2022.61) | 0.05(0.03 ~ 0.07) |
| Maldives | 2304.78(2050.73~2555.67) | 2451.48(2175.41~2751.74) | 8257.03(7362.13~9221.5) | 2268.24(1999.51~2573.46) | -0.42(-0.47 ~ -0.37) |
| Mali | 75410.22(66886.05~84285.71) | 1656.03(1463.16~1876.02) | 194654.71(172830.39~214824.16) | 1845.71(1626.52~2078.3) | 0.34(0.28 ~ 0.39) |
| Malta | 9804.59(8737.92~10921.29) | 2365.89(2112.34~2631.29) | 19680.65(17070.5~22217.94) | 2133.04(1879.62~2381.1) | -0.43(-0.48 ~ -0.38) |
| Marshall Islands | 414.92(375.97~460.94) | 2301.73(2041.52~2598.64) | 733.33(661.35~814.42) | 2024.13(1797.43~2296.13) | -0.42(-0.45 ~ -0.4) |
| Mauritania | 15331.38(13557.86~17156.95) | 1370.61(1208.18~1541.27) | 35872.07(31656.02~40877.54) | 1500.83(1316.64~1725.33) | 0.23(0.15 ~ 0.31) |
| Mauritius | 12302.92(11002.16~13953.98) | 1631.22(1442.97~1860.08) | 29226.67(25865.58~33113.12) | 1687.13(1499.78~1901.07) | -0.06(-0.13 ~ 0.01) |
| Mexico | 880124.61(777620.06~994941.08) | 2018.35(1783.92~2302.59) | 2747439.16(2421692.74~3093515.19) | 2238.95(1970.83~2525.77) | 0.32(0.23 ~ 0.4) |
| Micronesia (Federated States of) | 1256.37(1133.44~1390.14) | 2470.02(2199.02~2765.16) | 1616.67(1462.14~1822.76) | 2210.98(1956.06~2493.15) | -0.4(-0.42 ~ -0.37) |
| Monaco | 1673.04(1455.48~1919.02) | 2518.13(2208.02~2861.42) | 2220.05(1929.09~2511.77) | 2386.91(2120.48~2666.93) | -0.21(-0.25 ~ -0.16) |
| Mongolia | 24409.18(21906.29~27476.76) | 2185.01(1930.39~2489.62) | 48865.48(43338.29~54857.35) | 2187.97(1928.74~2468.99) | 0(-0.03 ~ 0.03) |
| Montenegro | 11175(9790.36~12715.2) | 1813.51(1590.8~2058.49) | 18890.01(16209.13~21406.07) | 2053.55(1782.44~2305.68) | 0.52(0.48 ~ 0.56) |
| Morocco | 266127.11(235131.99~302174.8) | 1745.14(1534.32~1986.55) | 796614.09(701585.19~903040.63) | 2361.44(2081.75~2665.44) | 0.98(0.96 ~ 1) |
| Mozambique | 95499.11(83410.52~107363.79) | 1412.61(1233.71~1601.78) | 210038.26(184700.06~234148.98) | 1571.19(1375.56~1771.66) | 0.21(0.16 ~ 0.27) |
| Myanmar | 649669.86(587939.27~710526.76) | 2779.98(2508.16~3054.35) | 1295568.44(1176296.9~1449782.55) | 2719.81(2465.81~3044.72) | -0.14(-0.15 ~ -0.12) |
| Namibia | 13767.05(12134.38~15535.3) | 1994.23(1749.44~2267.97) | 29661.18(26106.51~33277) | 2047.18(1801.24~2327.96) | 0.08(0.02 ~ 0.15) |
| Nauru | 110.59(99.5~122.18) | 2273.51(2008.22~2555.3) | 130.48(117.04~145.81) | 2138.45(1864.22~2421) | -0.25(-0.28 ~ -0.22) |
| Nepal | 290859.86(266391.69~310881.78) | 3049.02(2802.37~3266.97) | 710534.53(659222.25~765341.11) | 3051.13(2835.24~3271.74) | 0.05(0 ~ 0.1) |
| Netherlands | 468044.33(427331.75~520375.86) | 2405.38(2200.87~2673.25) | 759256.66(682277.2~845740.34) | 2263.34(2048.89~2513.4) | 0.08(-0.08 ~ 0.23) |
| New Zealand | 76495.2(66254.82~88259.19) | 1955.25(1694.11~2252.48) | 141262.39(118993.61~166031.43) | 1678.32(1429.32~1958.56) | -0.52(-0.62 ~ -0.42) |
| Nicaragua | 29753.75(26053.26~33678.3) | 1853.52(1618.86~2114.79) | 106608.73(93933.97~118461.8) | 2223.48(1965.05~2482.65) | 0.59(0.55 ~ 0.62) |
| Niger | 56102.13(49520.07~63023.08) | 1658.35(1440.53~1881.3) | 187856.39(166784.92~210573.07) | 1945.97(1710.19~2209.98) | 0.6(0.54 ~ 0.67) |
| Nigeria | 774959.81(679751.17~863696.07) | 1567.56(1359.31~1762.6) | 1897949.28(1672847.11~2120293.1) | 1788.63(1562.86~2005.8) | 0.45(0.41 ~ 0.5) |
| Niue | 49.17(43.09~55.41) | 2195.79(1936.13~2479.02) | 39.45(34.34~45.34) | 1920.92(1678.86~2190.63) | -0.52(-0.55 ~ -0.49) |
| North Macedonia | 44497.76(38928.08~50462.86) | 2427.41(2130.3~2742.78) | 81263.02(70963.47~94240.92) | 2624.09(2302.9~3004.59) | 0.26(0.2 ~ 0.32) |
| Northern Mariana Islands | 426.15(383.12~476.2) | 2014.26(1749.2~2296.27) | 884.67(766.56~1006.27) | 1867(1609.47~2151.45) | -0.33(-0.37 ~ -0.29) |
| Norway | 163566.25(142614.76~184351.22) | 2480.03(2193.59~2787.31) | 271735.88(240590.4~307387.89) | 2768.83(2464.33~3111.06) | 0.36(0.24 ~ 0.49) |
| Oman | 17092.82(15272.15~19098.42) | 2082.83(1848.99~2356.59) | 53844.05(47442.87~61113) | 2314.58(2036.61~2644.12) | 0.29(0.25 ~ 0.33) |
| Pakistan | 1508289.04(1340918.4~1682962.66) | 2643.8(2329.53~2967.91) | 3199227.56(2839704.75~3571679.34) | 2606.29(2311.9~2925.99) | -0.03(-0.06 ~ 0.01) |
| Palau | 225.05(201.97~252.94) | 2210.65(1934.41~2501.28) | 411.63(361.76~464.09) | 1988.29(1735.17~2245.21) | -0.42(-0.44 ~ -0.39) |
| Palestine | 18280.87(16306.16~20476.95) | 1951.02(1723.89~2212.09) | 57385.19(51328.76~64186.67) | 2147.67(1912.25~2436.2) | 0.3(0.27 ~ 0.33) |
| Panama | 24878.38(21735.36~28148.15) | 1637.43(1432.17~1858.99) | 83479.59(75166.92~92863.52) | 1863.21(1677.28~2075.51) | 0.27(0.21 ~ 0.33) |
| Papua New Guinea | 53449.83(49016.91~58244.58) | 2846.9(2595~3118.55) | 146155.33(134848.96~158586.85) | 2711.59(2480.62~2980.45) | -0.16(-0.18 ~ -0.15) |
| Paraguay | 45072.18(39722.5~51505.44) | 1954.9(1709.12~2251.08) | 125830.14(110754.62~142204.95) | 2153.44(1889.7~2446.94) | 0.29(0.26 ~ 0.31) |
| Peru | 176590.4(153812.79~203313.52) | 1404.73(1224.87~1625.35) | 523015.69(451412.92~597482.97) | 1538.03(1327.25~1755.86) | 0.43(0.3 ~ 0.56) |
| Philippines | 747489.49(668301.21~827636.34) | 2422.93(2133.86~2712.38) | 1835887.89(1631451.22~2046786.63) | 2213.41(1960.93~2482.71) | -0.44(-0.49 ~ -0.39) |
| Poland | 972530.58(852725.03~1094556.41) | 2273.76(2003.6~2550.63) | 1561336.31(1357982.65~1758296.56) | 2262.26(1997.88~2539.22) | -0.06(-0.11 ~ -0.01) |
| Portugal | 358990.55(328483.38~392127.8) | 2706.54(2483.56~2951.6) | 557534.33(501098.26~625041.88) | 2318.03(2088.23~2583.42) | -0.58(-0.65 ~ -0.52) |
| Puerto Rico | 55083.42(48243.85~62894.47) | 1530.93(1343.7~1745.27) | 128481.4(111721.45~143979.47) | 1802.96(1581.24~2026.47) | 0.46(0.38 ~ 0.55) |
| Qatar | 3861.59(3453.94~4293.33) | 2115.89(1863.92~2403.14) | 32092.35(28315.43~36059) | 2384.48(2104.19~2686.75) | 0.34(0.32 ~ 0.37) |
| Republic of Korea | 618590.42(542697.07~695835.06) | 2149.18(1882.65~2433.37) | 2090259.56(1904590.58~2305737.55) | 2287.85(2091.34~2510.81) | 0.34(0.09 ~ 0.6) |
| Republic of Moldova | 105998.3(95084.43~117873.67) | 2472.46(2220.62~2751.17) | 117569.83(102730.12~134548.38) | 2054.31(1805.95~2331.16) | -0.77(-0.83 ~ -0.71) |
| Romania | 679163.98(599854.52~765616.99) | 2527.95(2232.71~2842.2) | 849850.37(757217.45~951582.57) | 2367.71(2125.87~2635.41) | -0.25(-0.28 ~ -0.21) |
| Russian Federation | 4030459.09(3545658.45~4554661.87) | 2328.53(2055.06~2624.68) | 4982424.29(4358539.7~5631745.75) | 2196.02(1945.53~2463.55) | -0.28(-0.32 ~ -0.24) |
| Rwanda | 56484.96(50418.49~62750.36) | 1717.67(1528.15~1944.38) | 131916.63(116357.76~146789.63) | 1830.31(1621.65~2058.32) | 0.08(0.01 ~ 0.15) |
| Saint Kitts and Nevis | 420.47(361.64~479.73) | 1138.63(989.37~1296.51) | 960.61(824.14~1111.01) | 1457.74(1255.85~1677.64) | 0.78(0.7 ~ 0.86) |
| Saint Lucia | 1361.94(1185.09~1522.35) | 1520.62(1324.23~1713.84) | 4138.28(3601.03~4647.2) | 1761.21(1536.13~1974.36) | 0.46(0.41 ~ 0.51) |
| Saint Vincent and the Grenadines | 801.74(696.07~907.27) | 1093.03(950.19~1243.07) | 1811.44(1582.84~2061) | 1314.21(1156.11~1497.43) | 0.63(0.61 ~ 0.66) |
| Samoa | 2237.76(2024.62~2488.04) | 2558.6(2284.63~2887.52) | 3302.83(2952.38~3714.28) | 2293.9(2022.62~2584.23) | -0.4(-0.43 ~ -0.38) |
| San Marino | 820.93(716.11~927.58) | 2393.14(2095.26~2701.08) | 1604.16(1389.86~1828.37) | 2154.35(1897.78~2430.94) | -0.37(-0.39 ~ -0.35) |
| Sao Tome and Principe | 1218.1(1088.54~1365.49) | 1771.14(1580.29~2002.84) | 2647.29(2401.77~2927.19) | 2084.48(1858.31~2339.77) | 0.5(0.46 ~ 0.54) |
| Saudi Arabia | 102477.31(90288.31~115356.11) | 1500.78(1312.36~1711.53) | 448933.39(391042.3~508906.17) | 2050.86(1796.44~2325.25) | 1.08(1.04 ~ 1.11) |
| Senegal | 57046.51(50889.11~63717.16) | 1545.82(1379.87~1731.84) | 146764.79(129857.53~165174.18) | 1680.25(1479.89~1901.31) | 0.3(0.23 ~ 0.36) |
| Serbia | 256760.07(228918.85~289739.01) | 2382.85(2122.66~2676.2) | 429424.64(384715.39~479782.11) | 2692.46(2420.28~2989.34) | 0.45(0.41 ~ 0.49) |
| Seychelles | 940.29(823.04~1075.61) | 1616.22(1413.06~1858.05) | 2072.12(1811.66~2356.39) | 1835.81(1598.8~2078.76) | 0.28(0.23 ~ 0.33) |
| Sierra Leone | 36985(32589.99~41123.44) | 1608.64(1403.39~1817.18) | 79279.2(70634.84~88256.43) | 1770.38(1550.59~1987.35) | 0.28(0.23 ~ 0.34) |
| Singapore | 31393.2(27524.65~35651.49) | 1459.45(1274.5~1672.62) | 77467.27(67346.39~89381.77) | 922.56(805.95~1060.47) | -1.63(-1.72 ~ -1.55) |
| Slovakia | 112117.66(98314.53~127634.26) | 1916.55(1683.25~2175.87) | 192936.57(169685.02~218388.72) | 2118.52(1878.43~2394.73) | 0.37(0.36 ~ 0.39) |
| Slovenia | 52251.71(46288.17~58831.45) | 2157.07(1915.16~2427.36) | 94845.66(83121.86~106777.14) | 2177.73(1923.82~2436.97) | 0.03(0.02 ~ 0.05) |
| Solomon Islands | 3814.11(3480.01~4189.09) | 2689.84(2424.83~2989.04) | 9027.23(8290~9978.01) | 2490.64(2242.06~2783.82) | -0.26(-0.27 ~ -0.25) |
| Somalia | 55421.67(48913.53~61665.54) | 1818.53(1585.9~2049.83) | 163129.7(146092.73~181015.91) | 2116.82(1858.64~2404.17) | 0.39(0.33 ~ 0.45) |
| South Africa | 472664.62(422818.34~523652.37) | 2165.91(1915.93~2430.8) | 1004761.53(886683.61~1119026.86) | 2170.53(1917.47~2437.23) | -0.06(-0.11 ~ -0.01) |
| South Sudan | 42563.61(37069.44~47815.59) | 1462.63(1276.5~1647.56) | 71608.43(63234.73~80531.09) | 1601(1404.86~1818.43) | 0.16(0.1 ~ 0.23) |
| Spain | 1476183.64(1341035.01~1643766.01) | 2803.96(2554.06~3127.78) | 2570070.73(2308173.71~2861069.55) | 2658.24(2409.07~2950.54) | -0.19(-0.26 ~ -0.12) |
| Sri Lanka | 199962.81(176778.76~223981) | 1797.97(1578.41~2033.2) | 495569.1(428210.91~568689.16) | 1923.09(1670.3~2193.97) | 0.14(0.12 ~ 0.16) |
| Sudan | 229770.09(203416.87~256754.66) | 2319.16(2042.52~2601.73) | 518223.11(462333.66~578783.86) | 2416.13(2114.44~2730.3) | 0.02(-0.03 ~ 0.06) |
| Suriname | 3679.64(3242.29~4215.64) | 1389.28(1215.73~1605.01) | 9822.21(8646.97~11101.15) | 1581.99(1391.61~1787.28) | 0.37(0.32 ~ 0.42) |
| Sweden | 466178.22(415940.7~521175.27) | 3209.08(2898.46~3557.98) | 656865.55(580693.46~731419.81) | 3097.64(2760.32~3448.67) | -0.1(-0.2 ~ -0.01) |
| Switzerland | 281458.05(252648.89~314828.4) | 2766.29(2489.87~3086.66) | 451983.3(408103.36~501311.76) | 2548.98(2306.28~2820.47) | -0.38(-0.49 ~ -0.28) |
| Syrian Arab Republic | 115350.85(103137.98~128780.82) | 1980.98(1754.23~2235.69) | 307654.03(271843.5~349197.82) | 2446.57(2166.79~2749.06) | 0.68(0.65 ~ 0.71) |
| Taiwan (Province of China) | 289636.65(256122.21~325068.74) | 1932.15(1713.87~2155.84) | 624948.86(546926.12~716439.67) | 1512.21(1331.26~1725.43) | -0.86(-0.93 ~ -0.79) |
| Tajikistan | 73313.94(66040.73~81548.4) | 2522.65(2239.46~2841.52) | 149563.12(134319.29~167205.84) | 2519.36(2214.07~2838.91) | -0.03(-0.05 ~ 0) |
| Thailand | 810805.16(735671.03~883824.28) | 2128.97(1914.28~2332.72) | 1781016.61(1544547.25~2028248.09) | 1711(1490.46~1934.21) | -0.7(-0.73 ~ -0.68) |
| Timor-Leste | 7467.85(6643.94~8327.13) | 2333.86(2068.8~2654.4) | 19723.11(17602.39~22257.87) | 2295.43(2041.89~2596.01) | -0.21(-0.26 ~ -0.16) |
| Togo | 24566(21747.11~27450.02) | 1590.26(1393.99~1796.69) | 77409.93(67732.92~86985.9) | 1725.8(1505.49~1960.26) | 0.27(0.21 ~ 0.32) |
| Tokelau | 28.44(25.31~32.03) | 2176.87(1933.55~2457.95) | 27.66(24.06~31.46) | 1887.77(1641.64~2144.29) | -0.56(-0.59 ~ -0.53) |
| Tonga | 1293.51(1154.07~1450.11) | 2330.29(2045.18~2631.68) | 1578(1379.99~1791.24) | 1951.31(1690.26~2217.16) | -0.64(-0.67 ~ -0.62) |
| Trinidad and Tobago | 11128.73(9642.81~12558.69) | 1285.24(1117.94~1448.19) | 26719(23035.9~30689.73) | 1434.75(1240.91~1640.26) | 0.33(0.31 ~ 0.34) |
| Tunisia | 118240.84(104167.41~133967.55) | 2307.32(2031.06~2627.26) | 362516.48(323629.14~405804.54) | 2808.2(2494.08~3138.15) | 0.7(0.67 ~ 0.74) |
| Turkey | 1013999.41(917188.43~1123496.93) | 2829.81(2507.77~3180.37) | 2885480.52(2627311.65~3185404.7) | 3146.68(2861.31~3477.82) | 0.32(0.24 ~ 0.39) |
| Turkmenistan | 42649.04(37997.62~48146.66) | 2117.2(1862.96~2407.51) | 66967.53(59373.45~75767.23) | 1680.97(1478.82~1909.51) | -0.94(-1.04 ~ -0.84) |
| Tuvalu | 155.17(138.28~174.1) | 2392.12(2110.52~2684.5) | 213.45(189.13~242.69) | 2094.63(1836.1~2383.5) | -0.52(-0.55 ~ -0.49) |
| Uganda | 123349.76(108858.79~138441.86) | 1651(1443.19~1871.59) | 297985.24(265625.65~332702.05) | 1663.08(1467.43~1879.94) | -0.1(-0.16 ~ -0.05) |
| Ukraine | 1795617.2(1562004.76~2051136.9) | 2636.78(2310.89~2998.17) | 1457113.63(1254134.46~1665203.79) | 1978.64(1720.66~2252.75) | -1.06(-1.1 ~ -1.02) |
| United Arab Emirates | 18195.74(16725.11~19704.39) | 2326.06(2060.22~2605.85) | 180019.79(161070.89~203394.62) | 2686.42(2401.97~3008.25) | 0.38(0.34 ~ 0.42) |
| United Kingdom | 2782153.74(2482150.52~3078152.21) | 3170.87(2852.34~3489.52) | 4157373.18(3745187.24~4554835.6) | 3270.26(2957.04~3570.38) | 0.17(0.12 ~ 0.21) |
| United Republic of Tanzania | 171823.94(150513.28~192747.88) | 1381(1206.33~1560.55) | 456371.55(401902.84~513570.63) | 1538.19(1356.18~1744.38) | 0.25(0.17 ~ 0.32) |
| United States of America | 10252104.09(9292380.69~11182310.48) | 3224.12(2925.81~3516.13) | 19969706.53(18951147.4~20892543.95) | 3445.29(3263.49~3602.42) | 0.32(0.21 ~ 0.43) |
| United States Virgin Islands | 989.83(861.27~1148.83) | 1183.32(1031.3~1379.44) | 2557.19(2201.79~2931.81) | 1478.42(1288.27~1679.19) | 0.69(0.66 ~ 0.72) |
| Uruguay | 58807.63(51102.52~66971.79) | 1539.43(1344.57~1739.48) | 84284.35(73376.3~95642.79) | 1553.79(1365.19~1751.77) | 0.09(0.01 ~ 0.17) |
| Uzbekistan | 270841.52(240423.73~301835.95) | 2209.57(1939.21~2492.48) | 478246.5(425669.45~536721.47) | 1880.94(1663.83~2124.79) | -0.54(-0.57 ~ -0.52) |
| Vanuatu | 1703.75(1542.12~1882.2) | 2578.44(2309.33~2883.67) | 4179.33(3771.12~4619.88) | 2322.9(2051.12~2603.16) | -0.36(-0.38 ~ -0.35) |
| Venezuela (Bolivarian Republic of) | 168896.56(150202.83~189447.39) | 1679.96(1471.73~1892.15) | 616813.53(554663.96~695928.12) | 2120.81(1898.04~2390.93) | 0.72(0.66 ~ 0.79) |
| Viet Nam | 824262.6(732006.62~933555.33) | 1990.22(1756.48~2275.43) | 2203220.56(1961863.92~2483948.25) | 2291.31(2035.09~2592.2) | 0.34(0.3 ~ 0.39) |
| Yemen | 131173.15(117977.04~145423.09) | 2474.97(2201.61~2791.86) | 380327.76(339852.74~423976.21) | 2478.52(2169.71~2808.67) | -0.02(-0.09 ~ 0.04) |
| Zambia | 48305.68(42268.79~54490.95) | 1413.24(1237.48~1606.52) | 132605.08(116576.45~148088.04) | 1547.52(1346.26~1774.17) | 0.17(0.11 ~ 0.24) |
| Zimbabwe | 75882.75(65516.53~85894.65) | 1683.08(1461.65~1912.08) | 135132.28(118220.28~153081.68) | 1798.65(1573.57~2076.37) | 0.2(0.1 ~ 0.3) |

Table S2 The case number and ASR of deaths of COPD in 1990 and 2021 for both sexes by 204 countries and territories, with EAPC from 1990 to 2021.

| Location | 1990 | | 2021 | | EAPC(95%CI)1990-2021 |
| --- | --- | --- | --- | --- | --- |
|  | Number(95%UI) | ASR(95%UI) | Number(95%UI) | ASR(95%UI) |  |
| Afghanistan | 3577.63(2214.27~4760.88) | 62.22(38.16~82.85) | 3804.55(2729.01~4947.8) | 50.6(36.13~65.6) | -0.8(-1.01 ~ -0.58) |
| Albania | 660.26(566.94~768.26) | 40.47(34.68~47.31) | 650.27(503.04~880.3) | 15.57(11.92~21.04) | -3.1(-3.52 ~ -2.68) |
| Algeria | 1829.43(1451.04~2312) | 25.6(20.81~31.74) | 5316.02(4148.06~6533.78) | 22.08(17.4~27.31) | -0.03(-0.18 ~ 0.12) |
| American Samoa | 11.87(10.41~13.52) | 78.17(68.92~89.67) | 17.95(15.33~21) | 48.75(41.55~57.11) | -1.7(-1.8 ~ -1.6) |
| Andorra | 16.31(11.99~22.05) | 32.37(24.01~43.1) | 30.12(21.57~39.02) | 17.72(12.5~23.07) | -1.78(-2.06 ~ -1.5) |
| Angola | 1359.98(970.34~1788.79) | 49.94(36.19~64.52) | 2333.41(1765.73~3063.76) | 29.26(22.6~38.2) | -2.03(-2.13 ~ -1.93) |
| Antigua and Barbuda | 3.23(2.98~3.49) | 5.49(5.03~5.91) | 7.16(6.65~7.82) | 7.71(7.11~8.53) | 0.99(0.78 ~ 1.19) |
| Argentina | 8126.01(7584.33~8658.87) | 27.2(25.31~29.03) | 14312.38(13094.54~15431.02) | 24.51(22.43~26.4) | -0.24(-0.56 ~ 0.08) |
| Armenia | 1012.16(954.06~1067.33) | 43.3(40.45~45.79) | 658.74(568.12~752.34) | 15.19(13.08~17.33) | -3.03(-3.66 ~ -2.39) |
| Australia | 5654.61(5285.95~5957.69) | 28.76(26.87~30.34) | 9482.63(8304.2~10312.09) | 18.33(16.16~19.88) | -1.54(-1.8 ~ -1.28) |
| Austria | 2046.94(1890.2~2140.96) | 16.16(14.95~16.89) | 3268.59(2870.19~3515.65) | 15.69(13.94~16.79) | 0.2(-0.02 ~ 0.42) |
| Azerbaijan | 1249.71(1048.86~1495.59) | 29.65(24.75~35.61) | 1217.73(957~1659.4) | 15.34(12.08~19.77) | -2.36(-2.68 ~ -2.03) |
| Bahamas | 11.96(10.84~13.12) | 8.73(7.91~9.56) | 36.29(29.59~43.75) | 10.34(8.5~12.42) | 0.31(0.17 ~ 0.45) |
| Bahrain | 61.68(55.25~69.29) | 63.88(56.85~71.22) | 144.7(121.1~168.93) | 35.37(30.06~40.52) | -2.45(-2.89 ~ -2) |
| Bangladesh | 41548.89(31735.9~50629.89) | 101.94(77.88~125.49) | 71034.02(54260.07~92451.09) | 59.65(46.23~77.22) | -1.97(-2.31 ~ -1.63) |
| Barbados | 21.37(19.47~23.4) | 6.72(6.14~7.33) | 39.48(31.3~47.6) | 7.52(5.97~9.05) | 0.16(-0.06 ~ 0.39) |
| Belarus | 6249.34(5870.12~6626.49) | 50.32(47.12~53.37) | 1260.92(1045.45~1471.28) | 7.72(6.39~9.03) | -7.33(-7.99 ~ -6.66) |
| Belgium | 5521.33(5092.19~5848.24) | 34.3(31.64~36.26) | 5897.83(5006.38~6383.71) | 21.42(18.54~22.99) | -1.68(-1.83 ~ -1.53) |
| Belize | 11.88(10.85~12.91) | 13.05(11.9~14.18) | 53.23(45.77~60.47) | 20.61(17.68~23.5) | 1.09(0.47 ~ 1.71) |
| Benin | 634.23(478.95~772.21) | 36.54(27.58~44.63) | 981.49(741.88~1308.02) | 24.12(18.28~31.85) | -1.12(-1.34 ~ -0.89) |
| Bermuda | 6.71(6.16~7.28) | 11.85(10.92~12.81) | 13.42(11.42~16) | 8.67(7.39~10.36) | -1.22(-1.45 ~ -0.99) |
| Bhutan | 193.2(130.41~259.44) | 112.71(77.38~152.83) | 464.74(354.1~614.44) | 87.61(67.09~116.37) | -0.98(-1.09 ~ -0.86) |
| Bolivia (Plurinational State of) | 856.62(678.63~1038.94) | 35.81(28.63~42.98) | 1807.3(1381.89~2333.49) | 26.26(20.41~33.45) | -0.78(-0.84 ~ -0.72) |
| Bosnia and Herzegovina | 1046.32(932.94~1162.15) | 30.92(27.41~34.53) | 1057.2(837.8~1279.72) | 16.32(12.95~19.8) | -2.42(-2.67 ~ -2.17) |
| Botswana | 271.11(200.49~345.24) | 65.19(47.59~82.39) | 387.7(309~509.34) | 35.18(28.01~47.73) | -2.03(-2.17 ~ -1.88) |
| Brazil | 32343.85(30420.48~33652.74) | 45.48(41.87~47.78) | 63673.98(56870.93~67660.39) | 26.49(23.59~28.19) | -2.15(-2.4 ~ -1.9) |
| Brunei Darussalam | 48.07(40.92~56.53) | 63.76(53.82~75.88) | 68.8(59.06~81) | 33.64(28.55~39.83) | -1.59(-1.84 ~ -1.34) |
| Bulgaria | 3140.5(2825.51~3496.61) | 31.35(28.09~34.98) | 2240.73(1907.53~2605.18) | 15.24(13.01~17.69) | -2.44(-2.62 ~ -2.26) |
| Burkina Faso | 764.17(616.61~932.51) | 22.96(18.59~28.61) | 1340.71(1056.73~1678.09) | 18.51(14.97~23.72) | -0.6(-0.67 ~ -0.52) |
| Burundi | 1142.07(807.34~1411.15) | 58.84(41.78~73.08) | 1343.2(921.02~1739.88) | 37.72(26.31~48.99) | -1.87(-2.09 ~ -1.66) |
| Cabo Verde | 89.82(71.69~105.81) | 37.48(30.01~44.06) | 66.2(49.79~81.09) | 16.21(12.25~19.86) | -2.62(-3.38 ~ -1.86) |
| Cambodia | 1731.69(1305.43~2094.12) | 52.73(39.6~63.63) | 4071.36(3184.32~5023.1) | 46.08(36.74~55.7) | -0.47(-0.51 ~ -0.42) |
| Cameroon | 1307.51(1022.39~1591.91) | 38.6(29.89~47.21) | 2384.78(1842.26~3129.12) | 24.78(19.62~32.22) | -1.25(-1.39 ~ -1.12) |
| Canada | 8057.66(7399.67~8485.61) | 24.54(22.47~25.86) | 15147.24(13099.19~16369.27) | 18.46(16.12~19.95) | -1.08(-1.21 ~ -0.94) |
| Central African Republic | 547.48(355.11~723.39) | 66.96(41.72~88.9) | 844.76(525.26~1222.66) | 55.87(32.76~82.29) | -0.62(-0.67 ~ -0.58) |
| Chad | 826.72(577.37~1039.16) | 33.77(23.53~42.38) | 1389.02(976.85~1793.69) | 30.78(21.93~39.36) | -0.12(-0.22 ~ -0.02) |
| Chile | 2187.94(2069.69~2300.29) | 24.93(23.38~26.27) | 4234.86(3683.07~4567.1) | 15.97(13.91~17.2) | -0.83(-1.22 ~ -0.45) |
| China | 1237950.41(1065524.89~1384796.65) | 231.78(198.98~257.42) | 1285433.17(1044727.8~1539819.91) | 73.23(59.73~86.85) | -4.25(-4.47 ~ -4.03) |
| Colombia | 5481.23(5181.9~5705.74) | 37.37(34.87~39.08) | 17399.13(14352.79~20325.24) | 30.38(25.16~35.39) | -1.14(-1.33 ~ -0.95) |
| Comoros | 63.28(43.5~85.63) | 42.69(29.03~57.27) | 103.11(69.98~136.74) | 26.32(18.25~34.84) | -1.77(-2 ~ -1.55) |
| Congo | 465.87(303.24~654.62) | 59.34(38.4~83.54) | 674.31(510.7~886.06) | 36.18(28.3~47.33) | -1.83(-1.94 ~ -1.73) |
| Cook Islands | 5.07(4.12~5.93) | 53.54(43.81~62.64) | 6.12(4.94~7.55) | 24.84(20.09~30.67) | -2.76(-2.94 ~ -2.59) |
| Costa Rica | 405.84(369.26~433.88) | 25.62(23.2~27.42) | 1120.83(936.14~1258.99) | 19.73(16.61~22.08) | -1.19(-1.71 ~ -0.67) |
| Coted'Ivoire | 1021.32(820.87~1261.62) | 36.85(29.86~44.76) | 2003.76(1563.32~2568.04) | 24.43(19.57~30.76) | -1.16(-1.3 ~ -1.02) |
| Croatia | 1025.14(933.89~1112.59) | 18.99(17.35~20.63) | 1947.13(1708.15~2177.53) | 19.05(16.72~21.23) | 0.16(0.03 ~ 0.29) |
| Cuba | 1553.61(1464.7~1637.14) | 15.94(14.97~16.8) | 4669.73(4087.46~5257.03) | 22.57(19.78~25.41) | 1.17(0.99 ~ 1.35) |
| Cyprus | 299.21(211.86~365.46) | 60.33(42.38~73.65) | 460.18(382.78~539.92) | 27.02(22.71~31.62) | -2.69(-2.98 ~ -2.41) |
| Czechia | 2691.92(2474.13~2982.32) | 19.6(17.99~21.68) | 3903.45(3355.38~4406.64) | 16.6(14.24~18.8) | 1.04(0.41 ~ 1.68) |
| Democratic People's Republic of Korea | 17131.48(12286.65~23103.38) | 158.04(111.71~221.04) | 30211.24(22332.48~42248.3) | 107.68(80.67~156.7) | -1.23(-1.34 ~ -1.12) |
| Democratic Republic of the Congo | 5539.73(3778.55~7807.95) | 51.79(36.1~74.79) | 12256.81(7545.31~19734.58) | 47.53(29.56~79.56) | -0.3(-0.35 ~ -0.26) |
| Denmark | 2974.95(2794.85~3141.25) | 34.41(32.4~36.25) | 4821.98(4240.63~5196.95) | 35.43(31.37~38.12) | -0.2(-0.46 ~ 0.05) |
| Djibouti | 27.62(18.99~36.97) | 31.44(22~41.79) | 87.71(55.22~127.18) | 20.48(13.05~29.2) | -1.52(-1.67 ~ -1.36) |
| Dominica | 10.52(9.24~11.91) | 17.89(15.79~20.24) | 13.91(11.92~16.46) | 17.85(15.3~20.95) | -0.02(-0.05 ~ 0.01) |
| Dominican Republic | 415.13(350.06~491.35) | 14.91(12.56~17.47) | 1253.51(911.13~1920.48) | 13.11(9.55~20.04) | 0.16(-0.1 ~ 0.43) |
| Ecuador | 1141.15(1057.87~1206.47) | 26.68(24.77~28.18) | 2425.05(2005.52~2893.93) | 17.43(14.6~20.63) | -0.68(-0.94 ~ -0.41) |
| Egypt | 9230.85(7651.75~10545.02) | 48.3(40~55.61) | 10701.81(8682.52~13028.31) | 25.87(21.51~31.06) | -2.12(-2.31 ~ -1.93) |
| El Salvador | 663.93(582.51~766.14) | 23.95(20.99~27.55) | 1228.35(962.12~1565.47) | 17.68(13.73~22.56) | -0.98(-1.14 ~ -0.83) |
| Equatorial Guinea | 83.71(56.94~114.22) | 57.01(39.01~76.69) | 91.08(61.34~133.52) | 25.39(17.97~36.81) | -2.94(-3.2 ~ -2.67) |
| Eritrea | 398.8(277.78~509.15) | 51.09(32.89~66.81) | 707.04(535.24~913.85) | 36.51(28.01~46.83) | -1.1(-1.18 ~ -1.02) |
| Estonia | 193.71(180.07~208.22) | 9.64(8.98~10.33) | 192.6(168.09~216.82) | 6.13(5.33~6.91) | -1.49(-1.69 ~ -1.3) |
| Eswatini | 152.16(102.46~196.57) | 68.8(46.08~89.86) | 237.48(173.39~314.42) | 52.37(38.51~68.74) | -0.4(-0.83 ~ 0.03) |
| Ethiopia | 7037.1(4930.47~8525.93) | 44.91(31.46~54.86) | 8817.41(6883.1~10509.46) | 25.4(20.27~30.05) | -2.18(-2.31 ~ -2.06) |
| Fiji | 148.04(122.57~176.33) | 61.09(51.15~72.5) | 200.7(149.17~250.67) | 38.5(29.31~47.65) | -2.1(-2.41 ~ -1.8) |
| Finland | 916.76(853.92~975.76) | 12.4(11.52~13.23) | 1503.71(1311.34~1640.96) | 9.9(8.74~10.74) | -0.73(-0.9 ~ -0.57) |
| France | 16723.82(15234.77~17755.31) | 18.4(16.77~19.5) | 16593.37(14087.92~18170.9) | 8.9(7.7~9.66) | -2.76(-3.19 ~ -2.32) |
| Gabon | 216.11(170.15~273.56) | 45.09(35.44~57.11) | 204.17(146.77~292.27) | 26.25(19.14~37.88) | -1.85(-1.92 ~ -1.78) |
| Gambia | 97.63(72.85~124.87) | 35.83(26.74~45.23) | 244.02(168.49~330.75) | 30.68(21.08~41.46) | -0.31(-0.57 ~ -0.05) |
| Georgia | 557.52(483.14~639.12) | 9.83(8.49~11.24) | 700.55(597.21~812.8) | 10.97(9.36~12.74) | 2.84(2.13 ~ 3.56) |
| Germany | 29100.27(26561.14~30795.29) | 21.49(19.6~22.75) | 40495.89(35692.27~43610.14) | 18.28(16.51~19.46) | -0.23(-0.52 ~ 0.06) |
| Ghana | 692.95(551.15~850.95) | 14.94(12.03~18.21) | 1781.76(1402.27~2230.55) | 13.85(10.97~17.07) | 0.2(-0.1 ~ 0.49) |
| Greece | 2084.92(1915.67~2209.49) | 14.55(13.27~15.42) | 4996.07(4291.53~5422.84) | 14.98(13.08~16.14) | 0.7(-0.06 ~ 1.47) |
| Greenland | 20.45(17.05~23.25) | 85.37(69.16~97.02) | 23.8(19.69~28.59) | 42.98(34.48~52.82) | -2.14(-2.27 ~ -2.02) |
| Grenada | 9.08(8.21~10.01) | 11.27(10.25~12.41) | 13.88(12.06~15.5) | 13.62(11.91~15.08) | 0.21(-0.36 ~ 0.79) |
| Guam | 20.4(18.31~22.72) | 44.85(39.55~49.83) | 29.81(24.93~34.2) | 13.64(11.57~15.55) | -3.44(-3.78 ~ -3.11) |
| Guatemala | 611.07(552.17~662.27) | 28.1(25.61~30.19) | 1601.05(1383.44~1813.27) | 17.78(15.57~20.01) | -1.42(-1.68 ~ -1.15) |
| Guinea | 976.03(666.97~1251.14) | 34.81(23.81~44.76) | 1335.91(985.39~1759.21) | 28.71(21.16~37.24) | -0.26(-0.43 ~ -0.09) |
| Guinea-Bissau | 164.97(118.21~211.33) | 51.26(37.48~64.95) | 194.99(133.56~249.47) | 36.12(24.36~45.93) | -0.78(-1 ~ -0.55) |
| Guyana | 35.4(31.84~39.25) | 11.25(10.15~12.42) | 78.19(61.03~99.32) | 14.2(11.19~17.72) | 0.75(0.47 ~ 1.02) |
| Haiti | 970.53(428.24~1420.32) | 39.14(17.45~56.79) | 1889.33(834.96~2863.99) | 35.24(16.08~53.06) | -0.2(-0.27 ~ -0.13) |
| Honduras | 725.94(600.8~902.61) | 45.09(37.38~56.31) | 2767.08(2171.51~3486.73) | 56.63(44.76~70.3) | 0.96(0.73 ~ 1.19) |
| Hungary | 4855.6(4559.37~5161.5) | 34.89(32.75~37.07) | 5990.33(5208.42~6792.94) | 29.25(25.55~33.28) | -0.15(-0.62 ~ 0.32) |
| Iceland | 59.16(52.72~63.63) | 19.31(17.29~20.73) | 113.87(93.62~127.42) | 16.87(14.11~18.77) | -0.29(-0.46 ~ -0.12) |
| India | 407876.73(312072.19~489782.53) | 119.72(92.9~144.99) | 1066181.25(939670.33~1202912.45) | 108.39(94.73~122.39) | -0.1(-0.29 ~ 0.1) |
| Indonesia | 39061.94(28488.19~45421.14) | 53(37.97~61.85) | 85259.45(69822.72~101680.42) | 49.53(40.95~58.23) | -0.19(-0.31 ~ -0.06) |
| Iran (Islamic Republic of) | 3800.26(3087.39~4359.52) | 21.01(17.12~24.08) | 11440.37(9949.49~12568.57) | 17.44(15.06~19.2) | -0.32(-0.44 ~ -0.2) |
| Iraq | 887.53(678.16~1132.82) | 12.14(9.33~15.58) | 2006.34(1504.79~2512.34) | 11.44(8.67~14.41) | -0.74(-0.95 ~ -0.54) |
| Ireland | 2103.02(1977.12~2206.56) | 51.56(48.4~54.2) | 2003.62(1720.21~2215.53) | 23.26(20.03~25.66) | -2.68(-2.97 ~ -2.39) |
| Israel | 1039.24(954.7~1103.01) | 22.97(20.96~24.39) | 1665.67(1408.03~1821.47) | 12.08(10.31~13.15) | -1.84(-2.07 ~ -1.62) |
| Italy | 20138.83(18311.14~21102.48) | 22.31(20.15~23.44) | 28602.39(23503.69~31322.26) | 14.18(11.89~15.43) | -1.31(-1.49 ~ -1.14) |
| Jamaica | 226.19(207.9~241.82) | 12.16(11.17~12.99) | 473.55(369.69~592.77) | 14.7(11.51~18.47) | 0.36(-0.2 ~ 0.92) |
| Japan | 18862.16(17074.35~19703.26) | 12.24(10.95~12.87) | 32776.95(26625.89~35933.5) | 5.84(4.93~6.33) | -2.46(-2.75 ~ -2.18) |
| Jordan | 208.45(170.06~255.01) | 21.23(17.18~26.03) | 562.17(443.08~696.24) | 10.73(8.55~13.2) | -2.74(-3.04 ~ -2.44) |
| Kazakhstan | 5084.28(4545.25~5614.17) | 45.23(40.35~50.01) | 7144.87(6148.22~8309.05) | 46.68(40.28~54.1) | -0.26(-0.91 ~ 0.4) |
| Kenya | 2094.24(1220.99~3320.74) | 32.57(18.94~52.77) | 5823.53(3524.08~10425.88) | 34.58(20.66~64.8) | 0.4(0.27 ~ 0.52) |
| Kiribati | 28.07(17.3~42.22) | 105.25(66.66~167.66) | 39.56(28.68~62.42) | 83.25(62.48~133.85) | -0.88(-0.96 ~ -0.81) |
| Kuwait | 24.7(22.17~27.13) | 5.95(5.22~6.55) | 56.58(46.4~67.73) | 2.74(2.24~3.28) | -2.49(-3.01 ~ -1.96) |
| Kyrgyzstan | 2524.38(2322.62~2727.03) | 95.46(87.99~103.05) | 1066.54(896.54~1237.63) | 28.25(23.88~32.67) | -4.52(-5.06 ~ -3.99) |
| Lao People's Democratic Republic | 1390.74(898.03~1790.8) | 86.67(56.3~111.02) | 1945.69(1402.39~2564.42) | 56.4(41.34~72.81) | -1.64(-1.73 ~ -1.54) |
| Latvia | 449.16(410.79~490.05) | 12.58(11.52~13.69) | 299.4(254.74~348.52) | 6.94(5.9~8.11) | -1.83(-2.21 ~ -1.44) |
| Lebanon | 553.51(369.54~732.57) | 31.37(20.45~41.7) | 1309.43(1074.8~1565.03) | 19.65(16.16~23.51) | -1.19(-1.35 ~ -1.02) |
| Lesotho | 441.52(322.3~594.83) | 60.46(43.77~83.11) | 618.2(431.49~818.17) | 68.03(48.45~89.97) | 1.01(0.67 ~ 1.36) |
| Liberia | 325.8(251.9~396.15) | 34.79(26.5~41.49) | 420.66(310.99~576.82) | 26.55(19.69~35.93) | -0.79(-0.98 ~ -0.59) |
| Libya | 323.91(235.09~423.88) | 19.62(14.31~25.67) | 851.02(649.38~1144.31) | 19.96(15.43~26.6) | 0.61(0.39 ~ 0.83) |
| Lithuania | 1293.08(1201.31~1370.77) | 28.73(26.69~30.5) | 569.92(505.94~629.37) | 8.9(7.88~9.82) | -3.94(-4.11 ~ -3.77) |
| Luxembourg | 142.21(133.64~149.87) | 25.93(24.27~27.25) | 222.04(194.69~248.85) | 18.59(16.43~20.77) | -1.01(-1.14 ~ -0.88) |
| Madagascar | 2359.46(1880.06~2844.33) | 60.48(48.42~72.17) | 3940.52(2948.34~5196.79) | 54(40.77~70.83) | -0.54(-0.62 ~ -0.46) |
| Malawi | 951.39(750.44~1166.27) | 32.87(26.27~40.98) | 1683.99(1366.71~2085.8) | 29.2(23.86~35.62) | -0.6(-0.78 ~ -0.43) |
| Malaysia | 3284.74(2818.43~3779.23) | 41.73(35.85~48.11) | 6779.63(5913.15~7635.36) | 28.85(24.9~32.9) | -1.84(-2.16 ~ -1.52) |
| Maldives | 55.24(35.05~67.41) | 91.85(62.27~111.24) | 89.59(73.59~108.47) | 35.01(29.07~42.49) | -3.53(-3.65 ~ -3.4) |
| Mali | 1595.05(1308.65~1916.83) | 50.99(41.93~61.22) | 3091.48(2369.81~4071.23) | 44.4(35.43~57.08) | -0.18(-0.3 ~ -0.06) |
| Malta | 86.61(79.62~93.02) | 21.27(19.62~22.83) | 110.26(94.94~122.97) | 9.74(8.48~10.87) | -2.67(-2.9 ~ -2.44) |
| Marshall Islands | 12.86(9.52~15.37) | 103.12(78.69~122.01) | 15.69(11.66~20.67) | 71.32(53.51~90.51) | -1.1(-1.17 ~ -1.02) |
| Mauritania | 278.08(210.92~341.47) | 33.33(25.36~40.9) | 337.38(258.25~430.64) | 19.31(14.83~24.42) | -1.63(-2 ~ -1.26) |
| Mauritius | 184.72(173.27~195.04) | 33.48(31.24~35.45) | 276.62(252.65~293.91) | 16.88(15.32~18.02) | -2.35(-2.62 ~ -2.08) |
| Mexico | 13649.46(13026.8~13966.12) | 43.92(41.61~45.07) | 30707.02(27403.72~33861.86) | 27.87(24.9~30.7) | -1.46(-1.59 ~ -1.34) |
| Micronesia (Federated States of) | 44.56(31.78~55.73) | 115.41(85.11~149.45) | 33.9(26.27~44.37) | 67.13(53.27~86.18) | -1.89(-1.99 ~ -1.78) |
| Monaco | 11.35(8.77~13.8) | 13.67(10.63~16.48) | 14.67(11.3~18.16) | 12.09(9.45~14.93) | -0.29(-0.38 ~ -0.21) |
| Mongolia | 356.91(282.97~441.95) | 40.46(31.83~50.11) | 336.95(268.1~406.93) | 20.59(16.43~24.89) | -2.95(-3.21 ~ -2.69) |
| Montenegro | 33.37(28.04~40.15) | 5.71(4.78~6.88) | 52.88(41.69~64.2) | 5.87(4.63~7.15) | 0.17(-0.12 ~ 0.45) |
| Morocco | 2628.96(1726.69~3401.3) | 21.45(14.33~27.92) | 6708.77(5244.66~8061.88) | 23.52(18.52~28.25) | 0.5(0.38 ~ 0.61) |
| Mozambique | 1252.04(950.83~1554.5) | 28.34(21.53~35.33) | 2337.67(1776.18~2918.64) | 27.78(21.01~35.02) | 0.25(0.1 ~ 0.39) |
| Myanmar | 23723.21(17574.3~30698.86) | 135.53(99.61~173.15) | 40096.14(31826.48~48951.32) | 104.53(82.46~126.42) | -1.11(-1.22 ~ -1) |
| Namibia | 322.7(237.19~408.4) | 65.56(49.3~85.47) | 559.9(439.92~731.42) | 51.42(40.79~68.23) | -0.89(-1.09 ~ -0.68) |
| Nauru | 3.32(2.49~4.91) | 108.74(84.73~163.25) | 3.13(2.18~4.92) | 79.33(53.83~140.78) | -1.14(-1.27 ~ -1.02) |
| Nepal | 12363.71(8174.02~15780.64) | 176.12(121.67~222.55) | 27536.39(21511.83~34735.79) | 146.13(116.66~182.46) | -0.47(-0.69 ~ -0.24) |
| Netherlands | 6725.38(6174.11~7077.63) | 32.1(29.46~33.82) | 10731.24(9299.26~11612.43) | 26.93(23.51~29.07) | -0.82(-0.97 ~ -0.67) |
| New Zealand | 1303.22(1213.96~1362.76) | 32.67(30.31~34.2) | 2032.34(1765.71~2202.43) | 21.97(19.19~23.76) | -1.65(-1.85 ~ -1.46) |
| Nicaragua | 222.33(194.43~250.27) | 17.75(15.36~20.07) | 749.01(618.53~889.09) | 18.66(15.18~22.05) | 0.77(0.39 ~ 1.16) |
| Niger | 764.23(510~989.39) | 37.3(24.68~48.42) | 1700.65(1163.36~2280.18) | 28.5(18.98~37.47) | -0.53(-0.75 ~ -0.3) |
| Nigeria | 8692.06(6591.98~10978.34) | 24.21(18.34~30.04) | 12570.54(10544.56~15344.33) | 18.62(15.79~22.12) | -0.79(-0.87 ~ -0.72) |
| Niue | 1.81(1.52~2.14) | 74.44(62.89~88.19) | 0.97(0.78~1.19) | 48.45(39.22~59.64) | -1.63(-1.72 ~ -1.55) |
| North Macedonia | 444.05(386.53~524.17) | 27.61(23.87~32.95) | 559.08(415.95~785.32) | 21(16.05~28.34) | -1.15(-1.39 ~ -0.9) |
| Northern Mariana Islands | 6.83(5.57~8.48) | 68.86(57.65~82.74) | 16.28(14.32~18.71) | 47.04(40.84~53.5) | -1.29(-1.41 ~ -1.16) |
| Norway | 937.45(872.74~979.73) | 12.28(11.45~12.82) | 2843.89(2475.01~3042.39) | 24.65(21.65~26.3) | 2.22(1.77 ~ 2.68) |
| Oman | 100.93(73.57~130.14) | 18.62(13.38~23.96) | 182.84(147.54~236.7) | 14.24(11.62~17.63) | -0.26(-0.61 ~ 0.1) |
| Pakistan | 38904.91(30028.45~45411.87) | 83.97(64.44~98.28) | 64393.12(51864.17~81021.53) | 73.8(60.04~94.55) | -0.66(-0.91 ~ -0.42) |
| Palau | 6.03(4.95~7.38) | 82.99(68.59~101.4) | 9.91(8.01~12.09) | 70.54(57.96~84.93) | -0.37(-0.46 ~ -0.29) |
| Palestine | 194.93(145.56~241.05) | 27.88(21.05~34.67) | 287.51(243.08~348.24) | 16.34(13.91~19.89) | -1.95(-2.25 ~ -1.65) |
| Panama | 236.71(214.71~254.13) | 17.72(16.02~19.05) | 717.13(557.09~856.25) | 15.53(12.08~18.57) | -0.82(-1.18 ~ -0.46) |
| Papua New Guinea | 2243.24(1637.95~2941.43) | 188.53(140.8~243.16) | 5186.4(4049.01~6549.73) | 156.82(123.55~197.43) | -0.58(-0.62 ~ -0.54) |
| Paraguay | 334.79(288.44~392.33) | 17.04(14.65~19.96) | 988.05(787.48~1242.96) | 18.7(14.91~23.53) | 0.65(0.49 ~ 0.81) |
| Peru | 1373.74(1037.26~1697.84) | 12.98(9.86~16.05) | 3158.08(2190.03~4277.52) | 9.44(6.51~12.85) | -0.86(-1.03 ~ -0.69) |
| Philippines | 9040.74(8170.05~9975.96) | 46.58(41.87~51.28) | 21865.71(18313.68~25798.62) | 32.81(27.69~38.24) | -0.92(-1.01 ~ -0.84) |
| Poland | 10688.57(10275.71~10987.83) | 25.16(24.09~25.9) | 9126.21(8251.25~9904.58) | 11.8(10.69~12.81) | -2.19(-2.34 ~ -2.04) |
| Portugal | 3611.57(3385.24~3829.19) | 28.32(26.42~30.05) | 5702.86(4769.37~6270.62) | 17.55(14.93~19.18) | -1.73(-2.04 ~ -1.43) |
| Puerto Rico | 754.84(706.66~800) | 22.58(21.01~23.97) | 1553.56(1258.09~1821.58) | 16.98(14.01~19.81) | -1.76(-2.11 ~ -1.4) |
| Qatar | 15.49(12.57~18.56) | 29.4(24.12~35.54) | 50.9(39.46~65.07) | 12.79(9.99~15.94) | -3.26(-3.91 ~ -2.61) |
| Republic of Korea | 5319.9(4390.47~6194.54) | 28.73(22.89~34.09) | 10916.72(8858.9~13478.57) | 12.03(9.74~14.78) | -3.08(-3.34 ~ -2.82) |
| Republic of Moldova | 1726.94(1613.52~1849.07) | 45.41(42.69~48.18) | 695(615.5~780.1) | 11.54(10.25~12.95) | -4.99(-5.51 ~ -4.48) |
| Romania | 13043.23(12293.42~13825.82) | 54.76(51.27~57.77) | 6865.35(6056.65~7638.59) | 17.41(15.34~19.38) | -3.69(-4 ~ -3.38) |
| Russian Federation | 49321.59(47599.38~50204.13) | 29.81(28.54~30.43) | 31664.23(29115.3~34367.89) | 13.04(12~14.13) | -3.23(-3.58 ~ -2.88) |
| Rwanda | 1398.9(984.88~1697.3) | 64.53(46.29~78.59) | 1602.66(1091.73~2165.77) | 34.47(23.96~46.31) | -2.9(-3.24 ~ -2.56) |
| Saint Kitts and Nevis | 5.4(4.94~5.83) | 14.33(13.15~15.4) | 8.98(7.58~10.31) | 16.55(14.25~18.64) | 0.52(0.33 ~ 0.72) |
| Saint Lucia | 19.32(18.12~20.89) | 25.29(23.78~27.15) | 54.26(44.24~64.22) | 23.53(19.22~27.78) | -0.9(-1.22 ~ -0.58) |
| Saint Vincent and the Grenadines | 4.35(3.97~4.74) | 6.5(5.96~7.08) | 14.53(12.84~16.33) | 11.15(9.89~12.49) | 1.41(1.08 ~ 1.74) |
| Samoa | 66.79(49.32~86.11) | 101.24(75.55~135.45) | 80.68(63.62~99.48) | 69.57(54.86~85.98) | -1.26(-1.35 ~ -1.18) |
| San Marino | 4.85(4.04~5.74) | 12.78(10.64~15.07) | 6.18(4.33~8.44) | 6.05(4.27~8.3) | -1.35(-1.73 ~ -0.97) |
| Sao Tome and Principe | 36.87(30.84~43.73) | 64.61(53.85~75.99) | 50.7(38.3~63.9) | 58.43(44.21~72.16) | -0.15(-0.28 ~ -0.02) |
| Saudi Arabia | 1499.13(1094.89~1927.36) | 33.75(24.89~42.77) | 2803.87(2266.24~3415.31) | 22.69(18.55~27.31) | -1.45(-1.52 ~ -1.37) |
| Senegal | 891.03(725.67~1086.77) | 33.86(27.28~41.41) | 1612.79(1235.86~2068.23) | 25.94(20.06~33.05) | -0.54(-0.93 ~ -0.14) |
| Serbia | 2902.86(2478.78~3471.21) | 33.2(28.49~40.21) | 3456.41(2903.89~4065.45) | 19.76(16.6~23.28) | -1.84(-1.95 ~ -1.72) |
| Seychelles | 19.86(17~22.38) | 35.3(30.23~39.7) | 23.15(19.9~26.69) | 23.68(20.4~27.46) | -1.31(-1.45 ~ -1.17) |
| Sierra Leone | 594.99(467.69~727.14) | 34.13(26.77~41.62) | 774.58(549.91~1057) | 25.64(18.37~34.62) | -0.59(-0.77 ~ -0.42) |
| Singapore | 715.62(676.29~749.19) | 40.79(38.24~42.73) | 488.41(427.77~526.35) | 5.93(5.19~6.4) | -6.29(-6.41 ~ -6.18) |
| Slovakia | 781.56(692.89~876.95) | 13.33(11.79~14.92) | 934.22(784.41~1119.91) | 9.62(8.09~11.53) | -0.69(-0.84 ~ -0.54) |
| Slovenia | 646.5(605.39~683.84) | 26.2(24.47~27.77) | 608.81(522.39~671.55) | 11.49(9.92~12.67) | -3.34(-3.67 ~ -3.01) |
| Solomon Islands | 77.27(58.72~97.6) | 87.65(70.35~106.08) | 155.98(125.95~193.99) | 66.46(54.6~80.63) | -0.93(-0.99 ~ -0.87) |
| Somalia | 949.99(584.08~1276.02) | 58.26(35.7~77.92) | 1848.67(1097.12~2603.03) | 41.35(23.88~57.88) | -1.08(-1.14 ~ -1.01) |
| South Africa | 6391.78(5646.75~7787.6) | 36.18(31.67~44.58) | 13322.01(12247.33~14499.68) | 34.01(31.2~36.95) | -0.48(-0.88 ~ -0.08) |
| South Sudan | 1015.07(671.57~1353.32) | 47.35(31.45~62.62) | 1069.3(712.78~1493.36) | 37.74(25.3~52.04) | -0.93(-1.15 ~ -0.72) |
| Spain | 19235.79(17700.64~20330.1) | 35.29(32.23~37.39) | 29647.46(24925.77~32607.93) | 21.98(19~23.95) | -1.64(-1.81 ~ -1.47) |
| Sri Lanka | 6144.65(5288.53~6873.47) | 76.91(65.98~85.8) | 10679.06(7286.32~13972.08) | 46.21(31.82~60.72) | -1.16(-1.41 ~ -0.9) |
| Sudan | 2668.82(1136.65~4266.99) | 34.62(14.42~57.01) | 4013.24(2540.89~5759.74) | 25.51(16.28~36.36) | -1.08(-1.13 ~ -1.04) |
| Suriname | 44.15(39.2~49.29) | 19.56(17.31~21.84) | 90.7(69.88~115.62) | 15.32(11.86~19.65) | -0.57(-0.77 ~ -0.37) |
| Sweden | 1737.52(1614.47~1844.18) | 10.24(9.52~10.86) | 3601.06(3104.81~3987.76) | 13.51(11.69~14.92) | 0.95(0.63 ~ 1.27) |
| Switzerland | 2136.04(1981.38~2278.14) | 18.85(17.5~20.08) | 2496.43(2092.36~2760.71) | 11.31(9.69~12.43) | -1.49(-1.58 ~ -1.39) |
| Syrian Arab Republic | 1113.33(823.21~1421.82) | 25.62(18.9~32.53) | 2485.48(1897.51~3161.21) | 25.82(20.35~31.73) | -0.21(-0.34 ~ -0.08) |
| Taiwan (Province of China) | 3076.24(2875.93~3262.36) | 28.93(26.59~30.62) | 7796.58(6743.73~8582.28) | 17.06(14.87~18.72) | -2.05(-2.29 ~ -1.81) |
| Tajikistan | 1189.53(972.38~1466.62) | 48.45(39.12~59.98) | 1225.5(889.13~1656.75) | 29.1(20.8~38.89) | -2(-2.28 ~ -1.71) |
| Thailand | 15600.32(12919.45~18213.57) | 55.13(45.52~64.14) | 21391.88(16484.02~27003.5) | 19.8(15.22~24.97) | -4.05(-4.34 ~ -3.76) |
| Timor-Leste | 111.3(70.63~145.35) | 61.07(40.32~78.21) | 353.89(262.43~451.61) | 51.35(38.52~65.43) | -0.51(-0.61 ~ -0.4) |
| Togo | 330.71(254.06~395.25) | 35.09(26.98~42.06) | 793.45(584.19~1074.32) | 28.22(20.94~38.12) | -0.49(-0.66 ~ -0.33) |
| Tokelau | 0.82(0.57~1.11) | 68.84(48.23~91.92) | 0.67(0.49~0.98) | 45.39(33.12~66.2) | -1.52(-1.58 ~ -1.45) |
| Tonga | 28.49(23.12~33.42) | 65.16(52.91~76.25) | 36.64(29.76~44.11) | 48.96(39.77~58.69) | -0.87(-1 ~ -0.74) |
| Trinidad and Tobago | 103.46(97.36~109.34) | 14.63(13.77~15.44) | 202.95(156.9~251.14) | 10.78(8.34~13.35) | -1.1(-1.27 ~ -0.93) |
| Tunisia | 708.74(585.93~855.94) | 18.76(15.24~22.6) | 2021.51(1407.82~2900.02) | 17.61(12.17~25.98) | -0.34(-0.43 ~ -0.25) |
| Turkey | 16229.55(12848.83~18924.69) | 57.54(45.02~67.31) | 34044.47(28218.89~40618.51) | 40.77(33.86~48.52) | -0.72(-1.09 ~ -0.35) |
| Turkmenistan | 610.94(551.79~668.62) | 38.45(34.98~41.82) | 385.47(301.24~486.52) | 11.05(8.76~13.74) | -4.91(-5.63 ~ -4.19) |
| Tuvalu | 4.79(3.38~5.89) | 98.16(69.99~119.81) | 4.47(3.36~5.79) | 54.85(41.47~70.2) | -1.97(-2.05 ~ -1.9) |
| Uganda | 2259.13(1513.79~3035.73) | 44.84(30.24~59.36) | 3277.77(2197.48~4445.55) | 28.93(19.57~39.04) | -1.87(-2.02 ~ -1.71) |
| Ukraine | 33062.23(31211.9~34680.08) | 49.08(46.19~51.35) | 7471.14(5654.15~9388.64) | 9.39(7.1~11.8) | -6.78(-7.29 ~ -6.27) |
| United Arab Emirates | 126.09(94.83~170.09) | 38.06(28.41~49.81) | 428.24(338.64~532.75) | 26.26(18.3~33.46) | 0.6(-0.06 ~ 1.27) |
| United Kingdom | 32890.3(31221.83~33719.19) | 33.57(31.83~34.43) | 39383.25(34801.29~41582.49) | 26.22(23.42~27.57) | -0.61(-0.73 ~ -0.48) |
| United Republic of Tanzania | 2499.78(1940.06~3051.9) | 29.56(23.48~35.93) | 4389.73(3347.63~5593.3) | 21.64(16.78~27.41) | -1.24(-1.32 ~ -1.16) |
| United States of America | 89782.12(82909.7~93287.71) | 26.51(24.52~27.53) | 198036.53(172625.06~210192.16) | 31.32(27.47~33.15) | 0.48(0.27 ~ 0.69) |
| United States Virgin Islands | 8.27(6.95~9.79) | 12.79(10.85~14.92) | 13.66(10.45~18.16) | 7.66(5.88~10.2) | -1.53(-1.75 ~ -1.32) |
| Uruguay | 1190.69(1124.58~1246.74) | 30.26(28.57~31.65) | 2070.56(1890.97~2208.15) | 32.36(29.74~34.48) | 0.1(-0.07 ~ 0.27) |
| Uzbekistan | 2629.33(2259.68~3107.85) | 25.07(21.61~29.76) | 1735.94(1475.67~2052.92) | 8.75(7.46~10.28) | -4.44(-5.27 ~ -3.61) |
| Vanuatu | 48.87(30.84~65.18) | 115.53(74.14~152.7) | 100.89(63.2~133.23) | 83.39(54.05~112.14) | -1.2(-1.26 ~ -1.15) |
| Venezuela (Bolivarian Republic of) | 1533.99(1416.78~1618.49) | 18.86(17.26~19.98) | 6091.31(4713.68~7724.67) | 22.12(17.16~27.82) | 0.35(0.02 ~ 0.69) |
| Viet Nam | 18809.94(13992.11~23549) | 54.29(39.97~67.85) | 35336.52(27905.02~43547.67) | 43.96(35.11~54.17) | -0.66(-0.76 ~ -0.55) |
| Yemen | 1437.72(761.61~1989.1) | 38.05(20.06~53.44) | 3373.71(2433.7~4461.99) | 31.26(22.66~41.27) | -0.65(-0.74 ~ -0.57) |
| Zambia | 744.88(553.35~936.65) | 34.32(25.83~42.74) | 1535.09(1185.14~1939.56) | 29.44(23.12~36.83) | -0.69(-0.83 ~ -0.54) |
| Zimbabwe | 818.13(658.87~994.35) | 27.4(22.18~33.61) | 1453.82(1125.64~1826.89) | 28.99(22.37~35.79) | 0.59(0.31 ~ 0.88) |

Table S3 The case number and ASR of DALYs of COPD in 1990 and 2021 for both sexes by 204 countries and territories, with EAPC from 1990 to 2021.

| Location | 1990 | | 2021 | | EAPC(95%CI)1990-2021 | |
| --- | --- | --- | --- | --- | --- | --- |
|  | Number(95%UI) | ASR(95%UI) | Number(95%UI) | ASR(95%UI) |  |  |
| Afghanistan | 95309.77(64472.7~122651.44) | 1426.64(960.32~1838.69) | 113688.65(86374.09~144932.17) | 1155.17(875.13~1480.07) | -0.84(-1.05 ~ -0.63) | |
| Albania | 14007.1(12267.45~15862.04) | 759.54(662.9~864.71) | 14700.41(11935.5~18668.11) | 342.39(280.66~430.36) | -2.55(-2.88 ~ -2.23) | |
| Algeria | 56534.41(47029.66~67471.43) | 530.38(447.94~630.53) | 154501.17(131187.84~180952.82) | 488.58(415.38~573.16) | -0.09(-0.16 ~ -0.02) | |
| American Samoa | 317.7(283.06~357.83) | 1544.45(1371.26~1739.36) | 430.47(370.97~500.51) | 993.97(860.8~1150.4) | -1.6(-1.7 ~ -1.5) | |
| Andorra | 360.93(280.94~466.15) | 654.03(509.97~838.39) | 618.2(481.42~766.19) | 390.98(303.12~486.99) | -1.54(-1.76 ~ -1.31) | |
| Angola | 41093.22(30911.37~52004.64) | 1133.66(856.24~1425.55) | 75876.13(60578.58~93582.23) | 692.53(547.4~863.01) | -1.87(-1.95 ~ -1.79) | |
| Antigua and Barbuda | 74.2(68.01~80.38) | 132.15(121.23~143.46) | 181.83(167.49~196.94) | 179.35(165.54~194.02) | 0.84(0.7 ~ 0.98) | |
| Argentina | 179439.37(168594.73~190964.72) | 568.44(534.79~605.11) | 276792.75(257234.05~294660.1) | 487.71(454.18~519.7) | -0.43(-0.67 ~ -0.18) | |
| Armenia | 23943.26(22806.41~25251.53) | 918.66(869.63~967.9) | 14601.17(13080.16~16353.25) | 339.94(305.43~379.96) | -3.16(-3.59 ~ -2.72) | |
| Australia | 119322.35(112716.9~125692.66) | 598.16(564.83~629.03) | 174320.88(157434.8~186600.77) | 365.77(332.29~390.19) | -1.69(-1.92 ~ -1.47) | |
| Austria | 45933.94(43084.4~48995.57) | 380.49(357.14~406.3) | 70997.95(64998.03~76794.7) | 381.09(350.49~411.09) | 0.24(0.1 ~ 0.38) | |
| Azerbaijan | 32281.75(27941.18~37285.61) | 663.27(573.56~768.07) | 35021.69(28521~47582.76) | 374.49(309.17~479.57) | -2.29(-2.56 ~ -2.02) | |
| Bahamas | 320.58(293.62~350.6) | 210.29(191.81~229.66) | 931.93(776.01~1094.21) | 239.25(201.04~280.35) | 0.25(0.14 ~ 0.36) | |
| Bahrain | 1738.8(1581.99~1923.9) | 1203.52(1091.35~1332.11) | 4624.6(3987.56~5251.8) | 681.46(591.17~765.26) | -2.41(-2.76 ~ -2.06) | |
| Bangladesh | 1033465.94(813864.86~1227198.65) | 2281.06(1801.47~2722.49) | 1713712.75(1376728.77~2133832.83) | 1301.68(1050.53~1612.22) | -1.91(-2.13 ~ -1.69) | |
| Barbados | 466.93(430.36~506.66) | 153.44(141.23~166.25) | 885.27(731.51~1039.46) | 171.75(142.62~201.16) | 0.25(0.1 ~ 0.4) | |
| Belarus | 130976.82(123274.73~139224.98) | 1022.26(963.46~1084.37) | 38503.44(33454.47~44553.71) | 245.19(212.95~282.99) | -5.74(-6.26 ~ -5.21) | |
| Belgium | 108529.92(102049.92~114450.57) | 691.93(649.94~728.78) | 115161.65(103933.99~123893.68) | 477.58(437.24~512.88) | -1.35(-1.46 ~ -1.25) | |
| Belize | 279.77(258.06~303.68) | 294.92(271.92~318.85) | 1286.07(1136.05~1457.45) | 444.57(391.58~501.54) | 1.01(0.48 ~ 1.54) | |
| Benin | 16933.47(13464.77~20007.5) | 841.84(663.23~993.94) | 31985.46(26021.06~39965.07) | 604.11(487.65~760.4) | -0.89(-1.09 ~ -0.69) | |
| Bermuda | 152.11(139.14~165.4) | 251.73(230.95~273.39) | 278.32(240.64~322.83) | 198.74(171.34~230.34) | -0.95(-1.11 ~ -0.79) | |
| Bhutan | 5139.89(3614.64~6645.81) | 2374.53(1683.6~3107.79) | 9571.4(7479.21~12335.71) | 1678.89(1317.32~2166) | -1.3(-1.41 ~ -1.18) | |
| Bolivia (Plurinational State of) | 19476.63(15776.46~23052.73) | 667.98(546.14~789.81) | 37740.33(30140.7~47632.14) | 470.77(376.96~590.85) | -1.01(-1.04 ~ -0.97) | |
| Bosnia and Herzegovina | 26247.98(23742.18~28808.19) | 686.75(620.88~752.25) | 25257.6(21127.24~29835.25) | 403.71(338.66~474.43) | -2.01(-2.2 ~ -1.82) | |
| Botswana | 7340.97(5481.48~9202.72) | 1412.78(1065.18~1757.2) | 11491.82(9596.66~14337.45) | 840.65(700.98~1049.95) | -1.78(-1.91 ~ -1.65) | |
| Brazil | 742918.49(713094.38~774156.88) | 895.31(848.17~935.19) | 1380988.99(1280462.39~1456315.1) | 558.69(517.05~588.99) | -1.96(-2.2 ~ -1.72) | |
| Brunei Darussalam | 1004.95(875.43~1147.21) | 1140.97(985.18~1313.3) | 1588.86(1395.68~1833.67) | 595.82(519.54~686.73) | -1.85(-2.02 ~ -1.69) | |
| Bulgaria | 77854.38(70634.33~85530.15) | 672.25(608.95~740.13) | 54940.8(47895.68~62497.55) | 394.48(344.83~447.94) | -1.82(-1.95 ~ -1.68) | |
| Burkina Faso | 23922.06(20316.18~27869.9) | 568.38(478.52~665.91) | 46286.21(39032.65~55062.03) | 491.7(416.02~586.83) | -0.4(-0.46 ~ -0.33) | |
| Burundi | 30040.2(21892.4~36624.44) | 1309.37(961.52~1595.74) | 41563.16(30723.08~51254.7) | 877.53(646.02~1091.66) | -1.71(-1.91 ~ -1.51) | |
| Cabo Verde | 1896.57(1573.55~2204.39) | 802.09(669.17~926.73) | 1713.77(1397.49~2049.25) | 388.2(317.27~462.46) | -2.21(-2.81 ~ -1.62) | |
| Cambodia | 46326.26(36175.41~55184.62) | 1118.74(869.93~1325.37) | 102748.71(83618.21~124864.99) | 935.16(771.66~1124.75) | -0.63(-0.67 ~ -0.6) | |
| Cameroon | 37268.54(30624.14~43769.78) | 863.65(704.13~1027.1) | 80328.01(65856.81~99654.78) | 620.09(506.5~770.63) | -0.94(-1.05 ~ -0.83) | |
| Canada | 169205.25(158351.73~179809.26) | 511.66(478.41~544.39) | 299697.42(274496.96~323214.33) | 393.18(360.94~423.66) | -0.97(-1.09 ~ -0.86) | |
| Central African Republic | 16191.09(11140.48~20679.82) | 1514.36(1022.39~1953.54) | 27118.88(18600.06~37062.61) | 1302.89(863.86~1797.34) | -0.55(-0.59 ~ -0.5) | |
| Chad | 22745.63(17034.83~27655.61) | 803.11(598.74~972.2) | 45369.09(34999.83~55823.32) | 763.57(583.64~942.36) | -0.02(-0.1 ~ 0.07) | |
| Chile | 46553.19(44352.24~48841.02) | 484.47(462.17~508.31) | 73317.53(66461.85~78458.21) | 282.02(256.06~301.72) | -1.29(-1.57 ~ -1.01) | |
| China | 26097667.67(22790559.43~29216352.03) | 3852.57(3349.97~4279.01) | 23640320.96(19998658.36~27921931.16) | 1227.66(1048.45~1442.54) | -4.19(-4.37 ~ -4) | |
| Colombia | 126365.46(120251.38~131807.37) | 763.19(724.42~796.84) | 312592.99(266418.63~357444.81) | 559.45(477.79~640.18) | -1.52(-1.71 ~ -1.32) | |
| Comoros | 1767.09(1284.94~2341.2) | 943.55(681.95~1232.71) | 2883.65(2133.01~3608.71) | 615.46(455.41~774.81) | -1.59(-1.8 ~ -1.37) | |
| Congo | 12915.07(8984.26~17563.74) | 1293.88(892.44~1768.41) | 20598.91(16342.06~26280.82) | 825.18(673.44~1037.16) | -1.7(-1.81 ~ -1.59) | |
| Cook Islands | 122.07(102.34~141.12) | 1066.61(895.4~1222.27) | 133.52(112.41~157.9) | 534.64(449.7~630.68) | -2.44(-2.59 ~ -2.3) | |
| Costa Rica | 8225.04(7621.36~8738.92) | 487.63(451.54~518.91) | 20986.03(18547.6~23194.02) | 378.84(336.42~418.37) | -1.21(-1.63 ~ -0.79) | |
| Coted'Ivoire | 33393.65(28085.45~39734.14) | 842.6(704.68~1004.37) | 70462.55(57967.06~86542.61) | 617.34(510.65~756.38) | -0.89(-1.01 ~ -0.77) | |
| Croatia | 25938.27(23987.1~27958.25) | 444.88(412.36~478.73) | 38588.4(34658.96~42884.58) | 410.22(367.21~456.28) | -0.11(-0.2 ~ -0.02) | |
| Cuba | 36395.48(34225.44~38611.7) | 356.98(335.91~378.45) | 98637.54(87754.12~109681.85) | 501.11(445.8~556.48) | 1.07(0.89 ~ 1.26) | |
| Cyprus | 5754.65(4407.93~6787.63) | 921.04(690.89~1093.42) | 8663.62(7456.21~10028.55) | 455.12(393.6~521.74) | -2.37(-2.56 ~ -2.17) | |
| Czechia | 66073.17(60498.22~72876.92) | 484.17(443.76~533.14) | 90888.69(81256.94~101422.94) | 414.71(371.23~462.55) | 0.65(0.21 ~ 1.1) | |
| Democratic People's Republic of Korea | 369502.95(274052.12~475891.02) | 2799.28(2073.73~3681.84) | 602745.68(459884.79~787221.04) | 1968.2(1500.48~2612.25) | -1.13(-1.24 ~ -1.03) | |
| Democratic Republic of the Congo | 160137.29(114835.73~215086.01) | 1125.72(811.56~1536.86) | 363380.85(246025.18~522318.11) | 1073.41(720.28~1615.37) | -0.17(-0.21 ~ -0.13) | |
| Denmark | 64903.33(61263.13~68409.39) | 798.05(757.87~841.24) | 85639.25(78029.01~91949.74) | 682.66(625.85~729.99) | -0.84(-1.07 ~ -0.62) | |
| Djibouti | 901.65(665.37~1156.53) | 704.2(516.7~903.3) | 3067.49(2235.36~4106.62) | 506.44(362.23~680.9) | -1.21(-1.35 ~ -1.07) | |
| Dominica | 216.26(191.02~241.17) | 359.73(319.34~400.09) | 305.68(265.32~359.75) | 373.21(326.91~438.9) | 0.14(0.1 ~ 0.18) | |
| Dominican Republic | 9823.86(8538.1~11397.7) | 288.02(250.75~333.46) | 29187.99(22808.2~40487.7) | 294.88(229.69~409.07) | 0.47(0.32 ~ 0.63) | |
| Ecuador | 22371.19(21095.47~23591.69) | 454.07(427~478.81) | 45176.7(38805.05~52748.14) | 296.86(256.18~345.1) | -0.9(-1.11 ~ -0.68) | |
| Egypt | 256832.9(217213.57~288388.88) | 1015.46(857.09~1138.64) | 340087.26(288662.45~397678.49) | 602.17(511.84~700.27) | -1.92(-2.12 ~ -1.71) | |
| El Salvador | 13778.25(12242.09~15732.08) | 472.52(419.31~539.06) | 23560.17(19181.61~28573.61) | 361.12(294.3~437.05) | -0.93(-1.07 ~ -0.79) | |
| Equatorial Guinea | 2358.26(1662.46~3091.03) | 1292.92(929.68~1703.85) | 3021.16(2260.61~4048.43) | 613.98(458.71~833.3) | -2.72(-3.01 ~ -2.43) | |
| Eritrea | 13358.88(9949.49~16409.45) | 1202.89(861.07~1497.37) | 22655.8(18134.25~28098.96) | 844.47(676.17~1047.84) | -1.18(-1.26 ~ -1.1) | |
| Estonia | 5122.61(4743.47~5540.21) | 251.93(233.44~271.78) | 4676.34(4161.74~5224.79) | 170.09(151.14~190.77) | -1.26(-1.42 ~ -1.11) | |
| Eswatini | 4094.85(2917.72~5113.12) | 1513.96(1073.79~1893.29) | 7013.86(5354.04~9104.71) | 1264.8(979.92~1626.08) | -0.21(-0.65 ~ 0.23) | |
| Ethiopia | 215296.3(158828.23~252294.89) | 1093.38(817.17~1295.81) | 270087.37(220925.22~310041.64) | 619.18(510.02~708.79) | -2.18(-2.31 ~ -2.05) | |
| Fiji | 4098.74(3456.52~4798.96) | 1253.86(1066.77~1462.49) | 5203.44(4084.03~6385.25) | 778.59(613.56~940.77) | -2.01(-2.22 ~ -1.79) | |
| Finland | 23141.69(21586.58~24902.42) | 322.94(301.51~347.53) | 34286.16(31242.55~37492.78) | 259.79(237.73~283.43) | -0.77(-0.89 ~ -0.65) | |
| France | 298147.42(274952.4~317919.24) | 344.81(319.26~368.38) | 316491.39(282301.15~347195.19) | 208.64(188.85~228.62) | -1.87(-2.21 ~ -1.53) | |
| Gabon | 5295.71(4306.57~6564.62) | 970.41(788.18~1196.51) | 6009.93(4669.7~7929.73) | 616.69(481.4~822.05) | -1.54(-1.62 ~ -1.47) | |
| Gambia | 2977.56(2373.86~3644.8) | 839.39(665.37~1026.54) | 7467.01(5740.2~9533.42) | 744.46(564.13~962.59) | -0.26(-0.48 ~ -0.03) | |
| Georgia | 15306.51(13706.54~17112.33) | 253.78(227.99~281.96) | 17653.9(15566.54~19938.19) | 295.55(261.43~333.88) | 2(1.56 ~ 2.45) | |
| Germany | 633176.55(588740.17~674475.66) | 489.59(455.52~521.56) | 866758.29(790703.65~929152.32) | 442.86(409.16~475.53) | -0.11(-0.34 ~ 0.12) | |
| Ghana | 26214.62(22184~30788.03) | 410.5(348.32~481.99) | 71052.36(59466.21~83837.69) | 411.82(344.86~486) | 0.4(0.16 ~ 0.65) | |
| Greece | 53611.4(48683.97~58826.03) | 363.4(330.46~398.37) | 101175.18(90603.64~112074.91) | 380.35(340.27~424.19) | 0.61(0.13 ~ 1.09) | |
| Greenland | 489.41(420.81~545.11) | 1724.4(1446.21~1936.51) | 589.97(509.27~685.27) | 907.57(779.86~1067.9) | -2.02(-2.13 ~ -1.91) | |
| Grenada | 193.94(176.96~212.08) | 260.65(237.21~286.14) | 343.63(303.55~383.28) | 310.32(274.6~343.63) | 0.28(-0.1 ~ 0.67) | |
| Guam | 556.5(509.49~610.82) | 862.24(786.16~943.49) | 847.15(749.83~943.36) | 404.98(359.51~450.33) | -2.25(-2.48 ~ -2.02) | |
| Guatemala | 14997.11(13682.17~16209.06) | 512.58(471~552.44) | 35918.19(31597.92~40508.65) | 348.63(307.48~392.08) | -1.33(-1.61 ~ -1.05) | |
| Guinea | 26104.76(18926.42~31958.55) | 802.39(581.56~985.96) | 40917.5(32166~51029.82) | 713.48(558.98~891.5) | -0.07(-0.22 ~ 0.07) | |
| Guinea-Bissau | 4917.51(3658.77~6087.72) | 1216.2(911.72~1492.77) | 6749.07(5046.33~8375.64) | 887.63(660.38~1092.06) | -0.71(-0.92 ~ -0.51) | |
| Guyana | 901.07(813.26~988.11) | 249.26(226.01~272.73) | 2116.02(1706.98~2641.74) | 336.02(271.46~415.63) | 1(0.79 ~ 1.2) | |
| Haiti | 24742.62(11724.15~35648.55) | 819.75(392.57~1165.65) | 48350.73(23389.99~72580.24) | 728.06(356.16~1071.15) | -0.25(-0.31 ~ -0.2) | |
| Honduras | 17360.35(14702.34~20857.27) | 887.48(746.75~1076.22) | 60342.94(48626.42~74461.23) | 1049.64(846.1~1295.33) | 0.77(0.59 ~ 0.94) | |
| Hungary | 110952.3(104887.05~118203.93) | 776.75(734.04~826.31) | 140484.48(124620.13~157381.12) | 744.21(658.45~834.29) | 0.17(-0.19 ~ 0.53) | |
| Iceland | 1398.3(1291.15~1512.82) | 477.9(440.4~517.3) | 2409.57(2131.96~2661.81) | 397.5(353.63~437.69) | -0.57(-0.74 ~ -0.4) | |
| India | 10371214.8(8092449.81~12230914.78) | 2508.84(1975.51~2965.31) | 24018380.31(21633268.17~26798623.96) | 2171.16(1953.69~2422.39) | -0.36(-0.46 ~ -0.26) | |
| Indonesia | 1048854.24(810963.13~1203858.75) | 1140.65(870.7~1312.2) | 2235813.86(1869853.33~2637091.6) | 1040.41(874.72~1216) | -0.25(-0.34 ~ -0.16) | |
| Iran (Islamic Republic of) | 117495.42(99324.95~132902.21) | 478.53(405.07~540.13) | 314576.61(285133.04~342208.92) | 424.08(383.16~462.24) | -0.17(-0.27 ~ -0.08) | |
| Iraq | 28493.99(23263.44~33682.38) | 341.87(280.61~408.72) | 73090.57(60324.22~86600.25) | 320.73(262.89~381.13) | -0.53(-0.64 ~ -0.41) | |
| Ireland | 40658.81(38456.08~42654.41) | 975.14(917.99~1023.41) | 37059.92(33220.9~40422.08) | 451.69(406.89~492.35) | -2.62(-2.87 ~ -2.38) | |
| Israel | 21989.98(20274.01~23550.86) | 461.47(423.92~494.02) | 37085.7(33148.79~40842.32) | 292.37(263.81~320.7) | -1.29(-1.45 ~ -1.13) | |
| Italy | 400530.47(375611.1~422782.15) | 444.09(415.7~469.61) | 481343.52(420946.78~524958.28) | 285.38(256.09~309.66) | -1.33(-1.48 ~ -1.19) | |
| Jamaica | 5142.02(4737.58~5487) | 281.79(259.39~300.73) | 10489.15(8496.03~12940.67) | 335.42(271.26~413.45) | 0.31(-0.19 ~ 0.81) | |
| Japan | 446865.31(411745.77~483690.53) | 274.06(251.9~296.49) | 678591.72(590707.73~756041.37) | 155.76(137.62~174.26) | -1.78(-1.93 ~ -1.64) | |
| Jordan | 6714.21(5731.98~7856.61) | 511.14(436.06~598.7) | 22173.88(19008.79~25637.79) | 311.01(266.39~360.78) | -2.03(-2.25 ~ -1.81) | |
| Kazakhstan | 125012.58(111726.1~137793.85) | 1010.63(908.23~1113.48) | 176122.35(153267.75~202154.27) | 1023.13(889.04~1171.56) | -0.43(-0.99 ~ 0.14) | |
| Kenya | 57185.02(38939.77~81583.58) | 715.37(471.36~1054.73) | 166807.76(115815.95~257071.4) | 766.86(514.09~1250.53) | 0.42(0.27 ~ 0.56) | |
| Kiribati | 772.77(490.12~1104.07) | 2197.67(1410.58~3256.15) | 1102.82(817.32~1676.57) | 1684.31(1265.06~2595.52) | -0.97(-1.03 ~ -0.9) | |
| Kuwait | 1383.21(1235.93~1553.7) | 202.99(182.25~226.32) | 5039.7(4286.75~5886.41) | 160.46(138.49~185.65) | -0.77(-0.96 ~ -0.57) | |
| Kyrgyzstan | 57815.2(53532.32~62386.13) | 1977.09(1839.47~2129.62) | 28170.66(24378.1~32270.68) | 627.15(542.74~714.78) | -4.48(-5.02 ~ -3.94) | |
| Lao People's Democratic Republic | 36324.3(24221.93~46367.46) | 1862.13(1252.31~2347.83) | 49655.06(37453.62~63797.75) | 1176.83(892.84~1502.07) | -1.71(-1.8 ~ -1.63) | |
| Latvia | 11281.76(10244.5~12358.77) | 316.05(287.48~345.6) | 8287.59(7252.24~9468.67) | 214.69(185.89~243.6) | -1.24(-1.54 ~ -0.93) | |
| Lebanon | 14374.82(10572.54~18051.77) | 700.15(509.11~888.11) | 30023(26252.86~34370.41) | 477.84(416.89~545.89) | -0.94(-1.08 ~ -0.81) | |
| Lesotho | 11066.49(8559.06~14087.14) | 1355.16(1050.28~1752.63) | 17137.39(12499.11~22262.12) | 1616.31(1182.32~2071.61) | 1.1(0.77 ~ 1.43) | |
| Liberia | 8775.32(7096.39~10483.73) | 772.17(618.68~918.39) | 14129.41(11201.58~18062.36) | 652.08(513.7~830.56) | -0.48(-0.65 ~ -0.32) | |
| Libya | 9180.77(7244.95~11421.63) | 473.66(369.66~595.71) | 26120.49(21181.6~32666.35) | 504.59(410.08~628.23) | 0.53(0.38 ~ 0.67) | |
| Lithuania | 29072.34(27340.62~30906.14) | 644.22(605.75~684.04) | 14095.09(12524.64~15466.19) | 249.92(222.79~275.46) | -3.26(-3.42 ~ -3.09) | |
| Luxembourg | 3048.13(2863.47~3234.85) | 555.2(521.72~590.12) | 4397.48(3974.31~4849.88) | 399.59(360.41~442.07) | -1.08(-1.17 ~ -0.99) | |
| Madagascar | 61434.28(49954.44~72917.21) | 1263.53(1025.66~1497.36) | 120101.62(92583.3~153445.58) | 1147.8(892.85~1466.97) | -0.47(-0.54 ~ -0.4) | |
| Malawi | 27565.78(22338.88~32623.59) | 744.64(606.95~885.9) | 52175.42(43677.18~62489.71) | 704.6(592.39~839.1) | -0.41(-0.58 ~ -0.23) | |
| Malaysia | 78171.07(69568.66~87446.93) | 895.86(789.18~1003.19) | 167980.37(151924.5~184807.48) | 628.09(565.4~692.06) | -1.67(-1.9 ~ -1.44) | |
| Maldives | 1532.22(985.65~1874.03) | 1882.94(1269.67~2256.62) | 2125.86(1828.09~2479.23) | 696.35(597.42~814.59) | -3.66(-3.8 ~ -3.51) | |
| Mali | 46300.37(38712.15~54185.09) | 1174.28(980.64~1375.82) | 94056.55(75273.83~120846.27) | 1042.23(837.23~1324.98) | -0.17(-0.29 ~ -0.06) | |
| Malta | 1900.48(1768.46~2042.71) | 450.93(419.92~483.79) | 2474.95(2214.19~2771.39) | 244.56(218.62~274.13) | -2.14(-2.31 ~ -1.96) | |
| Marshall Islands | 348.85(263.62~415.24) | 2150.42(1637.24~2544.26) | 465.72(359.14~605.35) | 1467.49(1126.82~1862.39) | -1.14(-1.21 ~ -1.06) | |
| Mauritania | 7508.6(5974.1~8920.66) | 757.41(599.92~899.98) | 10337.01(8481.69~12262.52) | 482.86(395.79~574.92) | -1.35(-1.66 ~ -1.03) | |
| Mauritius | 4523.16(4265.34~4796.13) | 683.52(645.06~723.59) | 6730.16(6244.76~7258.29) | 388.62(361.01~420.1) | -1.96(-2.15 ~ -1.77) | |
| Mexico | 274428.18(264078.76~283443.69) | 739.18(708.08~763.46) | 615402.85(555650.05~675742.29) | 519.67(469.83~570.36) | -1.18(-1.28 ~ -1.07) | |
| Micronesia (Federated States of) | 1114.39(799.53~1378.31) | 2353.05(1717.15~2906.41) | 935.2(743.76~1205.61) | 1396.63(1121.89~1761.24) | -1.8(-1.91 ~ -1.69) | |
| Monaco | 242.62(202.28~282.42) | 333.93(281.51~385.14) | 299.33(249.28~351.2) | 292.09(246.1~339.51) | -0.38(-0.46 ~ -0.3) | |
| Mongolia | 8713.99(7090.15~10489.85) | 849.27(694.29~1029.74) | 9333.9(7792.55~10937.98) | 453.68(376.58~532.25) | -2.71(-2.94 ~ -2.49) | |
| Montenegro | 1139.01(990.09~1308.28) | 185.35(161.06~212.82) | 1760.15(1508.46~2053.29) | 187.53(161.34~217.71) | 0.1(0.03 ~ 0.18) | |
| Morocco | 71937.12(51247.24~89698.02) | 505.94(361.23~631.38) | 181710.71(149072~210580.28) | 557.91(461.15~645.66) | 0.45(0.37 ~ 0.53) | |
| Mozambique | 37308.3(30038.38~44794.99) | 656.87(524.85~788.92) | 76327.73(61074.42~91790.39) | 683.39(549.98~823.52) | 0.4(0.27 ~ 0.53) | |
| Myanmar | 574863.64(433591.74~729862.71) | 2710.62(2042.45~3435.53) | 860214.53(707432.67~1037228.73) | 1958.92(1604.09~2354) | -1.32(-1.43 ~ -1.21) | |
| Namibia | 8646.78(6568.57~10625.95) | 1431.13(1088.79~1767.53) | 15260.16(12270.42~18867.61) | 1171.77(951.35~1457.16) | -0.77(-0.97 ~ -0.57) | |
| Nauru | 95.82(73.91~130.94) | 2233.05(1739.46~3176.44) | 86.79(64.11~116.63) | 1614.56(1176.72~2426.49) | -1.18(-1.35 ~ -1.02) | |
| Nepal | 304773.04(206195.06~387547.96) | 3611.26(2493~4552.9) | 610592.94(484943.13~752714.44) | 2836.01(2275.31~3485.04) | -0.65(-0.86 ~ -0.44) | |
| Netherlands | 134725.03(126124.11~141586.61) | 658.81(616.49~692.81) | 197388.82(179645.47~212040.88) | 537.79(493.18~576.92) | -0.72(-0.85 ~ -0.6) | |
| New Zealand | 26895.1(25575.71~28189.26) | 671.68(638.18~703.55) | 37935.6(34308.49~40625.61) | 431.82(393.22~462.12) | -1.73(-1.88 ~ -1.57) | |
| Nicaragua | 5484.42(4958.17~6042.31) | 374.25(336.46~413.67) | 17250.74(14907.44~19802.55) | 381.7(327.58~437.44) | 0.44(0.16 ~ 0.73) | |
| Niger | 24112.49(17835.18~29715.04) | 876.25(635.55~1086.55) | 57550.23(43963.25~71564.15) | 702.13(524.46~871.29) | -0.41(-0.62 ~ -0.2) | |
| Nigeria | 256919.92(208905.65~308006.96) | 590.98(473.38~708.27) | 444788.06(382136.52~518374.1) | 490.54(424.15~569.06) | -0.59(-0.64 ~ -0.55) | |
| Niue | 34.29(29.07~40.33) | 1496.95(1271.72~1761.46) | 20.38(16.72~24.68) | 985.74(811.57~1191.85) | -1.63(-1.72 ~ -1.54) | |
| North Macedonia | 10523.06(9365.22~12015.88) | 599.24(533.68~688.26) | 13878.34(10924.74~18496.81) | 454.42(362.35~594.99) | -1.08(-1.22 ~ -0.95) | |
| Northern Mariana Islands | 219.18(177.62~272.44) | 1350.94(1137.98~1621.71) | 419.6(369.71~479.8) | 940.6(840.4~1055.79) | -1.17(-1.23 ~ -1.1) | |
| Norway | 22220.7(20841.14~23712.72) | 316.34(296.03~338.19) | 52691.26(48028.51~56308.12) | 497.3(456.34~532.24) | 1.46(1.08 ~ 1.84) | |
| Oman | 3444.42(2741.51~4230.55) | 488.48(388.97~597.76) | 7342.67(6306.72~8827.19) | 380.91(326.15~458.22) | -0.48(-0.65 ~ -0.31) | |
| Pakistan | 942115.38(754151.9~1077570.66) | 1783.98(1421.94~2048.46) | 1657341.7(1374667.51~1999430.95) | 1541.67(1287.52~1855.86) | -0.68(-0.9 ~ -0.46) | |
| Palau | 150.76(123.86~183.13) | 1639.14(1348.08~1990.14) | 247.74(202.84~295.83) | 1338.13(1120.27~1589.76) | -0.56(-0.62 ~ -0.5) | |
| Palestine | 4887.65(3905.13~5933.95) | 588.43(467.45~712.02) | 9335.17(8315~10596.72) | 397.17(353.23~454.73) | -1.43(-1.62 ~ -1.24) | |
| Panama | 5202.15(4852~5593.3) | 361.93(336.03~389.55) | 14074.36(11594.61~16339.78) | 313.24(258.1~363.23) | -0.86(-1.13 ~ -0.6) | |
| Papua New Guinea | 59576.22(43380.5~78495.4) | 3724.74(2788.16~4811.98) | 131563.96(102978.84~164213.6) | 3004.36(2404.29~3732.82) | -0.69(-0.73 ~ -0.64) | |
| Paraguay | 7441.02(6534.41~8573.27) | 347.01(304.21~401.38) | 21601.88(17738.49~26397.45) | 385.23(315.58~470.01) | 0.54(0.43 ~ 0.65) | |
| Peru | 32194.35(25575.42~38598.79) | 271(214.59~328.03) | 67067.86(51968.28~85453.23) | 200.04(154.29~255.74) | -0.86(-1 ~ -0.72) | |
| Philippines | 251066.6(229074.02~276505.18) | 961.37(882.15~1056.81) | 632847.53(553583.65~728840.2) | 807.05(707.12~923.78) | -0.45(-0.53 ~ -0.38) | |
| Poland | 260848.68(249832.11~273151.31) | 597.58(571.96~626.59) | 237264.77(216345.67~259512.32) | 327.79(298.61~358.48) | -1.75(-1.87 ~ -1.63) |  |
| Portugal | 77255.8(72857.53~82687.99) | 573.56(539.5~611.6) | 95187.06(85034.21~103420.94) | 344.54(312.27~373.1) | -1.83(-2.04 ~ -1.61) | |
| Puerto Rico | 14947.65(14097.49~15868.21) | 422.95(399~448.54) | 27122.97(23309.6~31270.67) | 354.55(305.58~406.86) | -1.29(-1.61 ~ -0.97) | |
| Qatar | 618.45(529.83~710.82) | 605.95(516.58~706.51) | 3225.91(2703.08~3773.31) | 336.21(287.54~395.65) | -2.19(-2.61 ~ -1.77) | |
| Republic of Korea | 130667.05(115578.35~147165.73) | 545.98(472.24~617.55) | 266056.06(230704.14~309042.98) | 289.13(251.75~335.7) | -2.09(-2.27 ~ -1.9) | |
| Republic of Moldova | 42720.13(39986.12~45605.57) | 1001.85(941.95~1066) | 18449.73(16631.28~20505.4) | 314.75(283.83~348.95) | -4.33(-4.71 ~ -3.94) | |
| Romania | 283407.62(267574.91~300106.22) | 1072.31(1010.94~1131.17) | 165985.4(150232.73~181410.83) | 459.19(415.1~503.41) | -2.7(-2.99 ~ -2.42) | |
| Russian Federation | 1199989.92(1161248.11~1238210.95) | 677.94(654.8~700.48) | 828949.94(770974.48~896492.56) | 352.01(328.27~379.88) | -2.74(-3.05 ~ -2.43) | |
| Rwanda | 39391.8(29175.08~47144.27) | 1430.48(1063.03~1702.37) | 48502.91(36946.07~61939.8) | 800.01(611.66~1028.73) | -2.74(-3.08 ~ -2.41) | |
| Saint Kitts and Nevis | 115.15(105.76~124.65) | 297.98(274.17~321.8) | 220.2(188.1~251.97) | 345.5(297.19~389.88) | 0.51(0.36 ~ 0.65) | |
| Saint Lucia | 411.43(386.2~442.92) | 487.08(458.95~523.15) | 1151.43(956.25~1360.8) | 487.49(404.87~574.54) | -0.42(-0.64 ~ -0.2) | |
| Saint Vincent and the Grenadines | 107.34(97.85~116.83) | 150.03(137.04~163.29) | 331.83(298.82~370.36) | 240.14(216.88~267.42) | 1.24(0.95 ~ 1.53) | |
| Samoa | 1587.33(1200.54~2030.44) | 1995.61(1503.27~2539.75) | 1884.73(1526.06~2351.12) | 1396.39(1123.6~1732.57) | -1.19(-1.28 ~ -1.1) | |
| San Marino | 107.75(93.34~122.48) | 297.48(259.03~337.09) | 148.42(118.97~181.07) | 183.81(148.11~222.92) | -1(-1.2 ~ -0.8) | |
| Sao Tome and Principe | 862.45(733.16~1003.69) | 1359.56(1157.63~1583.01) | 1366.83(1089.97~1689.46) | 1269.66(1005.65~1563.85) | -0.18(-0.33 ~ -0.04) | |
| Saudi Arabia | 39329.32(29518.08~49268.69) | 695.74(529.3~865.83) | 105295.17(87333.97~125544.82) | 533.08(452.57~618.8) | -0.92(-0.97 ~ -0.88) | |
| Senegal | 25240.23(21100.3~30124.72) | 784.23(657.4~937.47) | 48849.43(39869.59~58522.43) | 632.07(513.66~758.44) | -0.4(-0.73 ~ -0.07) | |
| Serbia | 68758.99(60033.93~79012.83) | 679.95(593.41~787.76) | 79310.95(67838.9~91575.57) | 475.15(406.91~548.67) | -1.28(-1.38 ~ -1.19) | |
| Seychelles | 442.12(384.73~490.48) | 772.86(673.87~857.06) | 586.31(520.17~655.93) | 538.59(475.57~600.26) | -1.21(-1.32 ~ -1.09) | |
| Sierra Leone | 16030.45(13166.43~18985.23) | 785.46(645.47~927.38) | 25126.53(19799.86~32414.88) | 646.88(505.84~831.93) | -0.34(-0.49 ~ -0.18) | |
| Singapore | 15179.72(14350.1~15920.82) | 770.14(729.76~806.94) | 12295.81(11019.29~13602.4) | 146.48(131.42~161.67) | -5.47(-5.59 ~ -5.36) | |
| Slovakia | 20595.51(18420.35~22509.59) | 346.5(309.9~378.7) | 25679.33(22461.25~29827.12) | 272.01(238.55~314.65) | -0.52(-0.63 ~ -0.4) |  |
| Slovenia | 14329.11(13506.51~15214.45) | 580.81(547.9~617.28) | 12947.44(11556.89~14366.26) | 277.59(245.36~308.42) | -2.85(-3.08 ~ -2.61) | |
| Solomon Islands | 2162.99(1662.6~2753.4) | 1791.8(1431.4~2207.36) | 4272.6(3482.75~5280.66) | 1368.16(1142.77~1653.18) | -0.89(-0.97 ~ -0.82) | |
| Somalia | 29614.57(20140.23~38697.48) | 1313.57(868.25~1712.3) | 64554.17(44390.96~85201.84) | 1040.23(683.92~1374.61) | -0.78(-0.84 ~ -0.72) | |
| South Africa | 182446.92(165137.6~209088.46) | 886.2(796.06~1027.79) | 382073.36(354297.22~418560.81) | 853.41(789.28~935.98) | -0.33(-0.65 ~ 0) | |
| South Sudan | 25953.8(18656.17~33634.53) | 1032.44(731.85~1333.2) | 31764.72(22898.62~42331.97) | 863.67(616.81~1149.37) | -0.8(-1.03 ~ -0.57) | |
| Spain | 364780.69(343482.64~387410.56) | 662.52(622.96~704.42) | 464294.78(411417.53~504946.94) | 410.16(370.27~444.09) | -1.68(-1.81 ~ -1.54) | |
| Sri Lanka | 137371.1(120714.58~153272.11) | 1428.46(1248.27~1590.9) | 219257.46(158404.22~280074.74) | 864.19(631.53~1103.11) | -1.4(-1.61 ~ -1.19) | |
| Sudan | 77076.28(40306.46~115598.16) | 823.9(425.43~1245.79) | 124034.06(87671.12~168666.28) | 623.17(442.16~841.65) | -1.01(-1.05 ~ -0.97) | |
| Suriname | 1029.46(932.54~1141.96) | 418.99(379.12~465.36) | 2212.57(1777.93~2703.1) | 355.3(284.8~434.64) | -0.4(-0.58 ~ -0.21) | |
| Sweden | 48165.69(44295.21~52855.98) | 314.04(287.47~343.6) | 78587.41(69696.62~86779.53) | 339.36(304.58~374.57) | 0.27(0.02 ~ 0.53) | |
| Switzerland | 47269.29(43851.23~50744.51) | 444.04(412.42~477.44) | 54985.02(48849.93~60797.14) | 289.27(259.16~319.86) | -1.3(-1.36 ~ -1.24) | |
| Syrian Arab Republic | 32688.02(25357.28~40253.74) | 605.31(469.6~746.18) | 73016(58644.53~89317.53) | 605.91(498.55~740.44) | -0.21(-0.31 ~ -0.1) | |
| Taiwan (Province of China) | 76410.55(71396.67~81955.77) | 577.34(539.63~617.83) | 148677.51(134659.01~163582.95) | 343.42(311.47~377.54) | -1.94(-2.11 ~ -1.77) | |
| Tajikistan | 29064.92(24844.22~34575.28) | 1063.76(900.02~1271.71) | 34175.52(26488.69~44558.64) | 637.12(492.25~826.72) | -2.04(-2.27 ~ -1.82) | |
| Thailand | 394009.04(340637.01~452099.64) | 1179.1(1010.81~1352.99) | 487665.25(401670.66~595684.92) | 461.07(381.24~559.88) | -3.7(-3.95 ~ -3.44) | |
| Timor-Leste | 3110.13(2079.62~3988.09) | 1262.75(875.76~1586.04) | 8459.53(6572.16~10395.08) | 1047.94(814.39~1288.81) | -0.58(-0.7 ~ -0.47) | |
| Togo | 10199.72(8402.4~11894.92) | 816.2(657.06~956.45) | 27088.28(21352.48~34667.65) | 705.07(558.67~907.89) | -0.28(-0.42 ~ -0.14) | |
| Tokelau | 18(12.67~24) | 1385.25(982.56~1830.78) | 13.64(10.38~18.29) | 921.21(704.89~1227.57) | -1.52(-1.59 ~ -1.46) | |
| Tonga | 684.41(567.86~795.33) | 1330.02(1114.81~1535.57) | 776.14(640~914.77) | 988.89(814.24~1161.4) | -0.91(-1.02 ~ -0.81) | |
| Trinidad and Tobago | 2368.08(2220.29~2529.5) | 295.55(277.46~314.49) | 4914.08(4008.45~5932.02) | 256.19(210.35~308.6) | -0.62(-0.76 ~ -0.47) | |
| Tunisia | 21987.07(18942.02~25578.41) | 463.75(400.76~538.26) | 58175.82(46751.59~74000.97) | 460.89(370.65~585.52) | -0.1(-0.15 ~ -0.04) | |
| Turkey | 391223.2(324510.76~450785.98) | 1199.96(985.75~1388.47) | 761035.41(651582.72~881753.18) | 850.19(727.99~983.51) | -0.9(-1.15 ~ -0.64) | |
| Turkmenistan | 15551.31(13952.16~17003.32) | 833.48(757.54~907.96) | 12562.38(10325.25~15261.61) | 307.79(254.46~369.5) | -3.97(-4.54 ~ -3.4) | |
| Tuvalu | 127.67(90.85~157.01) | 2060.07(1495.59~2496.17) | 110.33(85.61~139.32) | 1138.27(890.67~1437.52) | -2.01(-2.1 ~ -1.92) | |
| Uganda | 60525.38(43843.86~77417.56) | 979.47(704.19~1264.74) | 101881.68(75092.65~128956.84) | 693.4(506.33~880.76) | -1.59(-1.75 ~ -1.43) | |
| Ukraine | 741405.08(700220.53~780597.56) | 1055.36(999.59~1107.94) | 207495.33(169403.81~253607.89) | 274.39(225.05~334.21) | -5.68(-6.13 ~ -5.22) | |
| United Arab Emirates | 4904.78(3891.92~6303) | 897.36(701.58~1161.39) | 24096.07(20567.65~27878.77) | 594.58(488.75~713.85) | -0.24(-0.68 ~ 0.21) | |
| United Kingdom | 681686.82(653331.89~705879.76) | 728.64(699.01~754.24) | 774611.97(716224.9~816863.81) | 571.45(532.64~601.96) | -0.65(-0.73 ~ -0.57) | |
| United Republic of Tanzania | 72104.46(59089.29~84178.81) | 683.23(554.36~796.05) | 138364.36(113030.63~167939.79) | 543.49(445.23~659.63) | -0.95(-1.01 ~ -0.88) | |
| United States of America | 2347763.85(2209611.62~2482199.64) | 723.91(683.54~765.5) | 4646118.12(4302650.44~4904223.76) | 777.93(725.17~819.94) | 0.22(0.06 ~ 0.38) | |
| United States Virgin Islands | 205.49(175.82~236.92) | 266.67(230.13~308.34) | 324.35(264.21~398.89) | 182.91(149.95~224.07) | -1.14(-1.3 ~ -0.99) | |
| Uruguay | 25067.08(23916.85~26218.43) | 641.69(611.97~670.73) | 36505.19(33925.34~38640.38) | 642.28(600.48~679.05) | -0.07(-0.2 ~ 0.05) | |
| Uzbekistan | 66813.13(58893.44~75832.63) | 583.73(513.5~662.35) | 57699.91(49662.94~65971.16) | 238.39(206.48~271.83) | -3.86(-4.5 ~ -3.21) | |
| Vanuatu | 1364.21(883.82~1838.42) | 2338.24(1539.15~3050.72) | 2767.2(1799.96~3606.61) | 1709.11(1132.62~2218.1) | -1.2(-1.27 ~ -1.12) | |
| Venezuela (Bolivarian Republic of) | 37449.47(34958.44~39716.03) | 401.81(373.82~426.23) | 133543.28(106608.74~166502.8) | 462.77(370.7~574.17) | 0.23(-0.06 ~ 0.52) | |
| Viet Nam | 403156.32(308859.03~494535.79) | 1054.72(810.9~1299.01) | 776752.44(626580.15~929720.03) | 863.32(702.77~1024.54) | -0.6(-0.67 ~ -0.52) | |
| Yemen | 43515.26(27488.74~58384.96) | 905.15(560.94~1202.82) | 101184.24(79276.69~127402.3) | 730.21(567.09~920.09) | -0.73(-0.81 ~ -0.66) | |
| Zambia | 21553.65(16742.44~26022.22) | 782.84(607.9~948.22) | 48749.15(39118.55~59593.62) | 705.51(572.04~851.09) | -0.59(-0.73 ~ -0.46) | |
| Zimbabwe | 24488.64(20330.19~28522.98) | 644.41(540.4~755.08) | 47044.42(38407.65~56970.44) | 721.09(593.96~856.23) | 0.63(0.38 ~ 0.89) | |

Table S4 RRs of LRIs incidence for male and female due to age, period, and birth cohort effects.

| Factor | MaleRR(95%CI) | *P* | FemaleRR(95%CI) | *p* |  |
| --- | --- | --- | --- | --- | --- |
|  |  |  |  |  |  |
| age | | | | |  |
| 0 to 4 | 2.5269(2.5261~2.5278) | <0.001 | 3.1809(3.1799~3.182) | <0.001 |  |
| 5 to 9 | 0.8028(0.8026~0.8031) | <0.001 | 0.9843(0.9839~0.9846) | <0.001 |  |
| 10 to 14 | 0.4164(0.4163~0.4166) | <0.001 | 0.4774(0.4772~0.4775) | <0.001 |  |
| 15 to 19 | 0.3338(0.3337~0.3339) | <0.001 | 0.3583(0.3581~0.3584) | <0.001 |  |
| 20 to 24 | 0.3142(0.3141~0.3143) | <0.001 | 0.3246(0.3245~0.3248) | <0.001 |  |
| 25 to 29 | 0.3401(0.34~0.3402) | <0.001 | 0.3479(0.3478~0.348) | <0.001 |  |
| 30 to 34 | 0.3629(0.3627~0.363) | <0.001 | 0.3648(0.3646~0.3649) | <0.001 |  |
| 35 to 39 | 0.3814(0.3812~0.3815) | <0.001 | 0.3774(0.3772~0.3775) | <0.001 |  |
| 40 to 44 | 0.4653(0.4651~0.4654) | <0.001 | 0.447(0.4468~0.4472) | <0.001 |  |
| 45 to 49 | 0.5961(0.596~0.5963) | <0.001 | 0.5612(0.561~0.5614) | <0.001 |  |
| 50 to 54 | 0.6946(0.6944~0.6948) | <0.001 | 0.6491(0.6489~0.6494) | <0.001 |  |
| 55 to 59 | 0.7795(0.7793~0.7798) | <0.001 | 0.7303(0.73~0.7305) | <0.001 |  |
| 60 to 64 | 1.0658(1.0655~1.0661) | <0.001 | 1.0615(1.0611~1.0618) | <0.001 |  |
| 65 to 69 | 1.4805(1.48~1.4809) | <0.001 | 1.5317(1.5312~1.5321) | <0.001 |  |
| 70 to 74 | 1.7874(1.7868~1.7879) | <0.001 | 1.8497(1.8492~1.8503) | <0.001 |  |
| 75 to 79 | 2.0049(2.0043~2.0056) | <0.001 | 2.0347(2.034~2.0354) | <0.001 |  |
| 80 to 84 | 2.8218(2.8207~2.8228) | <0.001 | 2.6203(2.6194~2.6213) | <0.001 |  |
| 85 to 89 | 3.9753(3.9735~3.977) | <0.001 | 3.4699(3.4685~3.4713) | <0.001 |  |
| 90 to 94 | 4.7799(4.777~4.7829) | <0.001 | 4.1009(4.0988~4.1031) | <0.001 |  |
| 95 to 99 | 5.2667(5.2611~5.2723) | <0.001 | 4.428(4.4241~4.4318) | <0.001 |  |
| period | | | | |  |
| 1992 to 1996 | 0.9449(0.9447~0.945) | <0.001 | 0.9616(0.9615~0.9618) | <0.001 |  |
| 1997 to 2001 | 0.954(0.9539~0.9541) | <0.001 | 0.9677(0.9676~0.9679) | <0.001 |  |
| 2002 to 2006 | 0.9729(0.9727~0.973) | <0.001 | 0.9731(0.9729~0.9732) | <0.001 |  |
| 2007 to 2011 | 1.0123(1.0121~1.0124) | <0.001 | 1.0051(1.005~1.0053) | <0.001 |  |
| 2012 to 2016 | 1.0506(1.0504~1.0507) | <0.001 | 1.0308(1.0306~1.0309) | <0.001 |  |
| 2017 to 2021 | 1.0723(1.0721~1.0724) | <0.001 | 1.0658(1.0657~1.066) | <0.001 |  |
| cohort | | | | |  |
| 1897 to 1901 | 2.0696(2.0622~2.0769) | <0.001 | 1.6328(1.628~1.6377) | <0.001 |  |
| 1902 to 1906 | 1.9075(1.9045~1.9105) | <0.001 | 1.5719(1.5698~1.5741) | <0.001 |  |
| 1907 to 1911 | 1.7708(1.7692~1.7725) | <0.001 | 1.5621(1.5608~1.5634) | <0.001 |  |
| 1912 to 1916 | 1.6344(1.6332~1.6356) | <0.001 | 1.5181(1.5171~1.5191) | <0.001 |  |
| 1917 to 1921 | 1.594(1.5931~1.595) | <0.001 | 1.5616(1.5607~1.5624) | <0.001 |  |
| 1922 to 1926 | 1.4548(1.454~1.4555) | <0.001 | 1.4353(1.4346~1.436) | <0.001 |  |
| 1927 to 1931 | 1.3606(1.3599~1.3612) | <0.001 | 1.3672(1.3666~1.3677) | <0.001 |  |
| 1932 to 1936 | 1.3106(1.3101~1.3112) | <0.001 | 1.3645(1.3639~1.365) | <0.001 |  |
| 1937 to 1941 | 1.2438(1.2433~1.2443) | <0.001 | 1.3114(1.3109~1.3119) | <0.001 |  |
| 1942 to 1946 | 1.1924(1.192~1.1929) | <0.001 | 1.2434(1.2429~1.2439) | <0.001 |  |
| 1947 to 1951 | 1.1012(1.1008~1.1016) | <0.001 | 1.1384(1.138~1.1389) | <0.001 |  |
| 1952 to 1956 | 1.0264(1.026~1.0268) | <0.001 | 1.0578(1.0573~1.0582) | <0.001 |  |
| 1957 to 1961 | 0.9781(0.9777~0.9785) | <0.001 | 1.0173(1.0169~1.0177) | <0.001 |  |
| 1962 to 1966 | 0.9294(0.929~0.9297) | <0.001 | 0.9813(0.9809~0.9817) | <0.001 |  |
| 1967 to 1971 | 0.878(0.8777~0.8783) | <0.001 | 0.9427(0.9423~0.9431) | <0.001 |  |
| 1972 to 1976 | 0.8575(0.8572~0.8578) | <0.001 | 0.9335(0.9332~0.9339) | <0.001 |  |
| 1977 to 1981 | 0.8539(0.8536~0.8542) | <0.001 | 0.9313(0.931~0.9317) | <0.001 |  |
| 1982 to 1986 | 0.8351(0.8348~0.8353) | <0.001 | 0.8981(0.8978~0.8984) | <0.001 |  |
| 1987 to 1991 | 0.8053(0.8051~0.8055) | <0.001 | 0.8427(0.8424~0.843) | <0.001 |  |
| 1992 to 1996 | 0.7841(0.7839~0.7843) | <0.001 | 0.7938(0.7936~0.794) | <0.001 |  |
| 1997 to 2001 | 0.7459(0.7458~0.7461) | <0.001 | 0.7475(0.7473~0.7477) | <0.001 |  |
| 2002 to 2006 | 0.6798(0.6796~0.68) | <0.001 | 0.6826(0.6824~0.6828) | <0.001 |  |
| 2007 to 2011 | 0.5681(0.5679~0.5683) | <0.001 | 0.5691(0.5689~0.5693) | <0.001 |  |
| 2012 to 2016 | 0.445(0.4449~0.4452) | <0.001 | 0.4451(0.445~0.4453) | <0.001 |  |
| 2017 to 2021 | 0.3195(0.3193~0.3196) | <0.001 | 0.315(0.3148~0.3152) | <0.001 |  |

Table S5 RRs of LRIs mortality for male and female due to age, period, and birth cohort effects.

| Factor | MaleRR(95%CI) | *P* | FemaleRR(95%CI) | *P* |  |
| --- | --- | --- | --- | --- | --- |
|  |  |  |  |  |  |
| age | | | | |  |
| 0 to 4 | 13.0024(12.9464~13.0587) | <0.001 | 17.1266(17.0496~17.2039) | <0.001 |  |
| 5 to 9 | 0.4375(0.4349~0.4401) | <0.001 | 0.5765(0.5729~0.5801) | <0.001 |  |
| 10 to 14 | 0.1798(0.1785~0.1812) | <0.001 | 0.2474(0.2455~0.2494) | <0.001 |  |
| 15 to 19 | 0.1306(0.1295~0.1317) | <0.001 | 0.1694(0.168~0.1709) | <0.001 |  |
| 20 to 24 | 0.1221(0.1211~0.1231) | <0.001 | 0.1576(0.1562~0.1589) | <0.001 |  |
| 25 to 29 | 0.1397(0.1386~0.1408) | <0.001 | 0.1569(0.1555~0.1582) | <0.001 |  |
| 30 to 34 | 0.1687(0.1675~0.17) | <0.001 | 0.1475(0.1461~0.1488) | <0.001 |  |
| 35 to 39 | 0.2107(0.2093~0.2122) | <0.001 | 0.1697(0.1683~0.1712) | <0.001 |  |
| 40 to 44 | 0.271(0.2693~0.2727) | <0.001 | 0.2016(0.2~0.2033) | <0.001 |  |
| 45 to 49 | 0.3563(0.3543~0.3584) | <0.001 | 0.2418(0.2399~0.2438) | <0.001 |  |
| 50 to 54 | 0.4646(0.4621~0.4671) | <0.001 | 0.3661(0.3636~0.3687) | <0.001 |  |
| 55 to 59 | 0.6292(0.6262~0.6323) | <0.001 | 0.5168(0.5136~0.52) | <0.001 |  |
| 60 to 64 | 0.9659(0.9619~0.97) | <0.001 | 0.8715(0.867~0.8761) | <0.001 |  |
| 65 to 69 | 1.3695(1.3643~1.3746) | <0.001 | 1.3609(1.3549~1.3669) | <0.001 |  |
| 70 to 74 | 2.2413(2.2338~2.2488) | <0.001 | 2.2931(2.2844~2.3018) | <0.001 |  |
| 75 to 79 | 3.6759(3.6643~3.6875) | <0.001 | 3.6145(3.6021~3.6269) | <0.001 |  |
| 80 to 84 | 6.5224(6.5019~6.5431) | <0.001 | 6.2973(6.2768~6.3179) | <0.001 |  |
| 85 to 89 | 11.8349(11.7938~11.8762) | <0.001 | 10.8703(10.8326~10.9082) | <0.001 |  |
| 90 to 94 | 19.8761(19.7906~19.9619) | <0.001 | 19.6058(19.5264~19.6855) | <0.001 |  |
| 95 to 99 | 26.1589(25.9896~26.3292) | <0.001 | 32.0811(31.9112~32.2519) | <0.001 |  |
| period | | | | |  |
| 1992 to 1996 | 0.9439(0.9416~0.9462) | <0.001 | 0.9632(0.9606~0.9659) | <0.001 |  |
| 1997 to 2001 | 0.963(0.9609~0.9651) | <0.001 | 0.9753(0.973~0.9777) | <0.001 |  |
| 2002 to 2006 | 0.9779(0.9759~0.9799) | <0.001 | 0.9719(0.9698~0.9741) | <0.001 |  |
| 2007 to 2011 | 1.007(1.005~1.009) | <0.001 | 0.9909(0.9887~0.993) | <0.001 |  |
| 2012 to 2016 | 1.0567(1.0546~1.0589) | <0.001 | 1.048(1.0457~1.0504) | <0.001 |  |
| 2017 to 2021 | 1.0572(1.0548~1.0596) | <0.001 | 1.0546(1.0519~1.0574) | <0.001 |  |
| cohort | | | | |  |
| 1897 to 1901 | 2.7371(2.682~2.7934) | <0.001 | 2.4878(2.453~2.5232) | <0.001 |  |
| 1902 to 1906 | 2.5812(2.5559~2.6067) | <0.001 | 2.4037(2.3845~2.4231) | <0.001 |  |
| 1907 to 1911 | 2.459(2.4431~2.475) | <0.001 | 2.2805(2.2675~2.2936) | <0.001 |  |
| 1912 to 1916 | 2.3605(2.3486~2.3725) | <0.001 | 2.1166(2.1069~2.1263) | <0.001 |  |
| 1917 to 1921 | 2.2202(2.2107~2.2298) | <0.001 | 1.9976(1.9898~2.0054) | <0.001 |  |
| 1922 to 1926 | 1.9787(1.9713~1.9862) | <0.001 | 1.78(1.774~1.786) | <0.001 |  |
| 1927 to 1931 | 1.7891(1.7828~1.7954) | <0.001 | 1.6243(1.6189~1.6298) | <0.001 |  |
| 1932 to 1936 | 1.6491(1.6432~1.6549) | <0.001 | 1.555(1.5495~1.5606) | <0.001 |  |
| 1937 to 1941 | 1.5032(1.4975~1.509) | <0.001 | 1.4643(1.4582~1.4703) | <0.001 |  |
| 1942 to 1946 | 1.4016(1.3956~1.4076) | <0.001 | 1.3963(1.3896~1.4031) | <0.001 |  |
| 1947 to 1951 | 1.2649(1.2589~1.2709) | <0.001 | 1.2591(1.252~1.2662) | <0.001 |  |
| 1952 to 1956 | 1.1754(1.1693~1.1815) | <0.001 | 1.1576(1.1502~1.1651) | <0.001 |  |
| 1957 to 1961 | 1.1024(1.0961~1.1087) | <0.001 | 1.0973(1.0894~1.1053) | <0.001 |  |
| 1962 to 1966 | 0.9868(0.9806~0.993) | <0.001 | 1.0405(1.0323~1.0489) | <0.001 |  |
| 1967 to 1971 | 0.854(0.8481~0.8598) | <0.001 | 0.9256(0.9177~0.9335) | <0.001 |  |
| 1972 to 1976 | 0.8128(0.8069~0.8188) | <0.001 | 0.883(0.8752~0.8909) | <0.001 |  |
| 1977 to 1981 | 0.7976(0.7915~0.8037) | <0.001 | 0.8393(0.8318~0.8468) | <0.001 |  |
| 1982 to 1986 | 0.7231(0.7176~0.7287) | <0.001 | 0.7537(0.7472~0.7603) | <0.001 |  |
| 1987 to 1991 | 0.6467(0.6423~0.6512) | <0.001 | 0.6822(0.6772~0.6873) | <0.001 |  |
| 1992 to 1996 | 0.6076(0.6049~0.6103) | <0.001 | 0.6431(0.6401~0.6461) | <0.001 |  |
| 1997 to 2001 | 0.5127(0.5105~0.515) | <0.001 | 0.5462(0.5437~0.5488) | <0.001 |  |
| 2002 to 2006 | 0.4202(0.4184~0.422) | <0.001 | 0.4575(0.4554~0.4596) | <0.001 |  |
| 2007 to 2011 | 0.3291(0.3277~0.3305) | <0.001 | 0.3617(0.36~0.3633) | <0.001 |  |
| 2012 to 2016 | 0.2596(0.2585~0.2607) | <0.001 | 0.28(0.2787~0.2813) | <0.001 |  |
| 2017 to 2021 | 0.1907(0.1898~0.1916) | <0.001 | 0.1975(0.1965~0.1985) | <0.001 |  |

Table S6 Changes in DALYs of LRIs according to aging, population growth and epidemiological change from 1990 to 2021 at SDI quintiles and by GBD regions by sexes.

| Location | Sex | Overll difference | Aging（%） | Population（%） | Epidemiological change（%） |
| --- | --- | --- | --- | --- | --- |
| Global | Male | -61418683.29 | -13286999.83(21.63%) | 31270972.92(-50.91%) | -79402656.37(129.28%) |
|  | Female | -60220532.96 | -13955843.19(23.17%) | 28254075.36(-46.92%) | -74518765.13(123.74%) |
|  | Both | -121639216.3 | -27471135.78(22.58%) | 59542337.59(-48.95%) | -153710418.06(126.37%) |
| High SDI | Male | -275093.37 | 1342473(-488.01%) | 652141.71(-237.06%) | -2269708.09(825.07%) |
|  | Female | -430714.52 | 867754.06(-201.47%) | 447607.94(-103.92%) | -1746076.52(405.39%) |
|  | Both | -705807.89 | 2034099.32(-288.19%) | 1083478.45(-153.51%) | -3823385.66(541.7%) |
| High-middle SDI | Male | -4566055.59 | -234752.72(5.14%) | 1096729.75(-24.02%) | -5428032.62(118.88%) |
|  | Female | -4080517.13 | -408873.57(10.02%) | 780022.36(-19.12%) | -4451665.92(109.1%) |
|  | Both | -8646572.73 | -711245.91(8.23%) | 1867113.67(-21.59%) | -9802440.48(113.37%) |
| Middle SDI | Male | -20713341.85 | -3972171.97(19.18%) | 6777836.03(-32.72%) | -23519005.9(113.55%) |
|  | Female | -17521286.45 | -3580021.11(20.43%) | 5907396.77(-33.72%) | -19848662.11(113.28%) |
|  | Both | -38234628.3 | -7575308.73(19.81%) | 12721112.83(-33.27%) | -43380432.4(113.46%) |
| Low-middle SDI | Male | -22554811.19 | -7112178.52(31.53%) | 13547038.32(-60.06%) | -28989670.99(128.53%) |
|  | Female | -23117822.96 | -7476645.04(32.34%) | 13630841.68(-58.96%) | -29272019.6(126.62%) |
|  | Both | -45672634.15 | -14561370.38(31.88%) | 27185359.17(-59.52%) | -58296622.94(127.64%) |
| Low SDI | Male | -13278764.12 | -4314486.45(32.49%) | 22188268.54(-167.1%) | -31152546.21(234.6%) |
|  | Female | -15041204.2 | -4344932.75(28.89%) | 21003495.66(-139.64%) | -31699767.11(210.75%) |
|  | Both | -28319968.32 | -8652810.97(30.55%) | 43204183.32(-152.56%) | -62871340.67(222%) |

Table S7 The case number and ASRs of incidence, mortality, and DALYs of LRIs predicted until 2045.

| Year | Incidence | | Mortality | | DALYs | |
| --- | --- | --- | --- | --- | --- | --- |
|  | ASR | case | ASR | case | ASR | case |
| 2022 | 4452.842754 | 362889444 | 31.541485 | 2436375 | 1325.934297 | 96015562 |
| 2023 | 4407.943592 | 365508156 | 31.150506 | 2451026 | 1309.5118 | 95195853 |
| 2024 | 4363.187235 | 368802877 | 30.761841 | 2474908 | 1293.121478 | 94684794 |
| 2025 | 4339.104137 | 373737093 | 30.508455 | 2513092 | 1282.889211 | 94808491 |
| 2026 | 4315.027726 | 378843889 | 30.255355 | 2555501 | 1272.660311 | 95123355 |
| 2027 | 4290.954376 | 384199086 | 30.002269 | 2601490 | 1262.43266 | 95530356 |
| 2028 | 4266.872637 | 389856030 | 29.74894 | 2649745 | 1252.202757 | 95976415 |
| 2029 | 4242.747099 | 395575875 | 29.494757 | 2698204 | 1241.963594 | 96424258 |
| 2030 | 4231.373512 | 402491640 | 29.368669 | 2759480 | 1236.033528 | 97224806 |
| 2031 | 4220.007139 | 409512606 | 29.242576 | 2823784 | 1230.103311 | 98048514 |
| 2032 | 4208.658533 | 416726603 | 29.116503 | 2892873 | 1224.174778 | 98918596 |
| 2033 | 4197.296767 | 424181334 | 28.98986 | 2965837 | 1218.24199 | 99838640 |
| 2034 | 4185.900344 | 431676223 | 28.86226 | 3039091 | 1212.299539 | 100759233 |
| 2035 | 4185.239074 | 440328237 | 28.851235 | 3124949 | 1210.000459 | 101990503 |
| 2036 | 4184.598346 | 448966159 | 28.840606 | 3213252 | 1207.7044 | 103231994 |
| 2037 | 4184.001623 | 457657008 | 28.830639 | 3307254 | 1205.415409 | 104512901 |
| 2038 | 4183.418996 | 466439208 | 28.8203 | 3406214 | 1203.12495 | 105843257 |
| 2039 | 4182.82012 | 475130448 | 28.80941 | 3504441 | 1200.828295 | 107163308 |
| 2040 | 4181.078699 | 483771143 | 28.795476 | 3601609 | 1198.257826 | 108439092 |
| 2041 | 4179.355639 | 492312109 | 28.781937 | 3698593 | 1195.688794 | 109697104 |
| 2042 | 4177.689405 | 500944679 | 28.769501 | 3800175 | 1193.129116 | 110972941 |
| 2043 | 4176.061608 | 509785033 | 28.757568 | 3907672 | 1190.574706 | 112277983 |
| 2044 | 4174.410625 | 518532936 | 28.745201 | 4012846 | 1188.01397 | 113544908 |
| 2045 | 4172.738791 | 527174530 | 28.732551 | 4114596 | 1185.44835 | 114762037 |
